# Large-scale brain network modulation by auricular vagus neuromodulation in major depressive disorder: a systematic review of neuroimaging evidence

**DOI:** 10.3389/fpsyt.2026.1972200

**Published:** 2026-09-30

**Authors:** Ying Su, Yuanyuan Liu, Jun Chen, Xiaodan Li, Tianzhu Liu, Wenbin Fu

**Affiliations:** 1 The Second Clinical College of Guangzhou University of Chinese Medicine, Guangzhou, China; 2 Department of Traditional Therapy, Guangdong Provincial Hospital of Chinese Medicine, Zhuhai Hospital, Zhuhai, China; 3 Department of Radiology, Guangdong Provincial Hospital of Chinese Medicine, Zhuhai Hospital, Zhuhai, China; 4 Department of Gynecology, Guangdong Provincial Hospital of Chinese Medicine, Zhuhai Hospital, Zhuhai, China; 5 Faculty of Medicine, Faculty of Chinese Medicine, and State Key Laboratory of Mechanism and Quality of Chinese Medicine, Macau University of Science and Technology, Macau, China

**Keywords:** functional connectivity, functional neuroimaging, resting-state fMRI, transcutaneous auricular vagus nerve stimulation, treatment response

## Abstract

**Background:**

Auricular vagus neuromodulation, encompassing transcutaneous auricular vagus nerve stimulation (taVNS) and transcutaneous electrical cranial–auricular acupoint stimulation (TECAS), is an emerging noninvasive intervention for major depressive disorder (MDD). Neuroimaging studies of its central effects have expanded rapidly, yet findings remain dispersed across heterogeneous designs and have not been integrated at the cohort level.

**Methods:**

We followed PRISMA 2020 guidelines and searched five databases (PubMed/MEDLINE, Embase, Cochrane CENTRAL, Web of Science Core Collection, and PsycINFO) from inception to June 30, 2026; only English-language reports were included. Two reviewers independently screened records, extracted effect-level data, and appraised methodological quality using the 2024 JBI tool for quasi-experimental studies. Reports from the same cohort were identified via trial registrations, and denominators were counted once per cohort. Findings were stratified by five imaging paradigms and synthesized via cohort-level vote-counting, with null effects reported alongside positive findings.

**Results:**

Seventeen neuroimaging reports arose from nine independent cohorts (321 enrolled patients with MDD, 293 in imaging analyses, 144 healthy controls). The most reproducible finding was bidirectional normalization in striatal–cortical reward-circuit regions across four cohorts, qualified by null nucleus accumbens and putamen seed findings in one cohort. Default-mode-network regions were implicated in seven of nine cohorts, with heterogeneous directions. The only anterior insula effect surviving between-group correction was confined to an acute stimulation-state paradigm, and stimulation-state connectivity changes were frequency-band-specific. Twenty-seven of the coded effects were explicitly null. The sole machine-learning model (AUC = 0.856) was evaluated only by internal cross-validation. Quality appraisal rated two reports high, fourteen moderate, and one low.

**Conclusion:**

The evidence remains strictly hypothesis-generating: auricular vagus neuromodulation is accompanied by distributed treatment-associated brain changes, but establishes neither causal pathways nor clinically actionable imaging markers. Confirmatory multi-center, sham-controlled trials with preregistered imaging endpoints, harmonized stimulation parameters, explicit null-result reporting, and independent external validation are needed.

**Systematic review registration:**

https://www.crd.york.ac.uk/PROSPERO/view/CRD420261421762, identifier CRD420261421762.

## 1 Introduction

Major depressive disorder (MDD) affects more than 280 million people worldwide and is a leading cause of disability (1). Its core features—persistent low mood, anhedonia, cognitive impairment, and functional decline—contribute to excess mortality, reduced quality of life, and substantial socioeconomic costs (1). Available treatments include antidepressant medications (2), psychotherapy, electroconvulsive therapy (ECT) (3), and repetitive transcranial magnetic stimulation (rTMS) (4). Nevertheless, many patients respond inadequately to initial treatment, others develop treatment-related adverse effects, and some fail to maintain remission after initial improvement (5). Safe, effective, biologically grounded treatment strategies remain urgently needed.

Conventional transcutaneous auricular vagus nerve stimulation (taVNS) and transcutaneous electrical cranial–auricular acupoint stimulation (TECAS) are noninvasive auricular vagus neuromodulation approaches under investigation for MDD (6, 7). Both stimulate the auricular branch of the vagus nerve but differ in technique. Conventional taVNS targets the cymba conchae or tragus, where vagal afferent fibers are dense, and typically uses low-frequency stimulation (20 Hz or alternating 4/20 Hz). TECAS, rooted in traditional Chinese medicine, stimulates defined auricular acupoints, sometimes combined with scalp acupoints such as Baihui (GV20) and Yintang (EX-HN3), and uses different parameters (e.g., the 4/20 Hz dense-dispersed wave). These differences in montage, frequency, and concurrent acupoint engagement may recruit partially distinct afferent pathways and central circuits. Neither approach requires surgical implantation; both engage central autonomic and limbic pathways, carry fewer procedural risks than invasive VNS—whose implantation can cause infection, hoarseness, and voice alteration (8)—and are convenient for repeated, long-term use (9). Clinical evidence suggests that auricular vagus neuromodulation can improve depressive symptoms with favorable safety and tolerability (10), yet its neurobiological basis remains poorly understood, limiting protocol refinement and patient selection.

Neuroimaging offers a way to examine the central effects of neuromodulation (11). Contemporary models frame MDD as disruption of distributed large-scale networks rather than of discrete regions; these networks support cognitive control, salience detection, self-referential processing, and reward processing (12). Studies have therefore used resting-state functional magnetic resonance imaging (rs-fMRI), structural MRI, diffusion tensor imaging (DTI), and other techniques to characterize the neural response to auricular vagus neuromodulation. Findings implicate several depression-relevant networks, notably the default mode network (DMN) (13), salience network (SN) (14), frontoparietal/cognitive control network (FPN/CCN), and limbic–corticostriatal reward circuit (15). These networks interact rather than operate independently: the DMN and SN show anticorrelated dynamics, with engagement of one typically accompanied by suppression of the other; the FPN couples flexibly with the SN during cognitively demanding tasks; and the reward circuit shares nodes with the DMN, including the ventromedial prefrontal and posterior cingulate cortices (14, 16–18). How auricular vagus neuromodulation reshapes these between-network interactions may be central to its antidepressant effects.

Previous reviews and meta-analyses have summarized the clinical efficacy of taVNS for depression (7) and its mechanisms, parameters, and applications across neurological and psychiatric conditions, but none has focused on neuroimaging evidence in MDD or integrated findings across analytical approaches—functional connectivity, independent component analysis (ICA), ALFF, ReHo, FCD, and machine learning—into a network-level framework. This literature is fragmented: stimulation protocols, imaging modalities, analyses, treatment duration, and outcome measures vary widely across studies (19). Direct comparison is further complicated by the use of distinct imaging metrics, including functional connectivity (FC), amplitude of low-frequency fluctuations (ALFF) (20), regional homogeneity (ReHo) (21), functional connectivity density (FCD) (22), and machine-learning models (23). Individual studies thus report numerous imaging alterations, but convergent changes across large-scale networks have not been systematically integrated.

To address these gaps, we systematically reviewed neuroimaging studies of auricular vagus neuromodulation in MDD, with four objectives: (1) to consolidate the available neuroimaging evidence; (2) to identify the large-scale networks and principal brain regions repeatedly modulated by treatment; (3) to integrate findings linked to clinical improvement and candidate imaging predictors of treatment response; and (4) to formulate a network-level account of the central effects underlying antidepressant action.

## 2 Methods

### 2.1 Search strategy

The review followed the Preferred Reporting Items for Systematic Reviews and Meta-Analyses (PRISMA 2020) statement (24). Five databases—PubMed/MEDLINE, Embase (Elsevier), the Cochrane Central Register of Controlled Trials (CENTRAL), Web of Science Core Collection, and PsycINFO (Ovid)—were searched from inception through June 30, 2026. Search strategies combined controlled vocabulary (Medical Subject Headings, Emtree, and APA Thesaurus terms) with free-text terms in three conceptual blocks: (1) major depression; (2) auricular vagus neuromodulation, including transcutaneous auricular vagus nerve stimulation (taVNS) and transcutaneous electrical cranial–auricular acupoint stimulation (TECAS); and (3) neuroimaging, encompassing functional and structural MRI, functional connectivity, network/connectome analyses, and electroencephalography (EEG) terminology, the last included to maximize retrieval sensitivity. No language or publication-type restrictions were applied at the search stage; reports using EEG as the sole neuroimaging modality and reports in languages other than English were excluded at screening under the pre-specified eligibility criteria. Full database-specific strategies, including the Embase strategy rebuilt with Emtree explosion terms and the complete auricular-vagus/TECAS synonym set, are provided in **Supplementary Table S1**. Reference lists of eligible articles and relevant reviews were hand-searched for additional publications. The protocol was registered with PROSPERO (CRD420261421762). Three deviations from the registered protocol were made: expansion from three to five databases, addition of a linked-report identification procedure (§2.3), and replacement of the 2020 JBI critical appraisal tool by the 2024 revised version (§2.5). Reports using EEG as the sole modality were excluded because, unlike MRI, EEG cannot localize treatment-related changes to the large-scale distributed networks and MNI-coordinate-based regions that frame this synthesis, and source-localized EEG findings cannot be integrated with the MRI evidence base within a common anatomical framework.

### 2.2 Eligibility criteria

Eligibility followed the PICOS framework. Population: adults (≥18 years) with major depressive disorder diagnosed by DSM or ICD criteria, including first-episode drug-naïve, recurrent, and treatment-resistant presentations; studies enrolling minors or participants without a primary MDD diagnosis were excluded. Intervention: auricular vagus neuromodulation—taVNS (including transauricular VNS) or TECAS targeting the auricular branch of the vagus nerve; invasive VNS, manual acupuncture, and non-auricular body electro-acupuncture were excluded. Comparator: sham stimulation, active pharmacological comparators (e.g., escitalopram), active within-subject stimulation-condition comparators (e.g., a second active stimulation parameter setting), healthy comparison participants, or within-subject pre-treatment baselines; no comparator was required, and single-arm pre–post studies were eligible. Outcomes: treatment-related magnetic resonance neuroimaging outcomes, including resting-state fMRI indices (ALFF/fALFF, ReHo, seed-based and whole-brain functional connectivity, independent component analysis/functional network connectivity), task-based fMRI activation, graph-theoretical/connectome topology and network-based statistics, machine-learning neuroimaging features, and structural or diffusion MRI measures. Study design: original peer-reviewed pre–post, controlled (sham- or active-controlled, randomized or non-randomized), or single-arm neuroimaging studies, including acute single-session and longitudinal (4–8-week) paradigms; cross-sectional studies without a treatment-related imaging endpoint, reviews, meta-analyses, editorials, letters, conference abstracts, protocols without results, case reports, and animal or *in vitro* studies were excluded. Eligible reports were in English and reported neuroimaging findings adequate for qualitative synthesis.

### 2.3 Study selection

Retrieved citations were imported into EndNote X9.1 (Clarivate, Philadelphia, PA, USA), and duplicates were removed before screening. Two reviewers independently screened titles and abstracts and then assessed full texts against the pre-specified eligibility criteria; disagreements were resolved by discussion, with unresolved decisions referred to a third reviewer.

Multiple reports from the same parent trial were identified through trial registration numbers, ethics approvals, author groups, and participant overlap. Following PRISMA 2020 guidance on companion reports, linked reports were treated as a single study unit: each report contributed its imaging metric or stimulation timepoint, whereas participant denominators were counted once at the cohort level.

Database searches yielded 1,131 records across five databases; after removal of 293 duplicates, 838 unique database records remained, and together with 4 reports identified by hand-searching reference lists, 842 records underwent title and abstract screening. Full texts were sought for 39 reports; 22 did not meet the criteria, and 17 neuroimaging reports from 9 independent participant cohorts entered the qualitative synthesis. Reports excluded after full-text assessment are listed with their reasons in **Supplementary Table S2**, and the complete selection pathway is presented in **Figure 1**. Inter-reviewer agreement supported reliable study selection at both screening stages; percentage agreement at each stage is reported in the **Supplementary Methods**.

**Figure 1:**
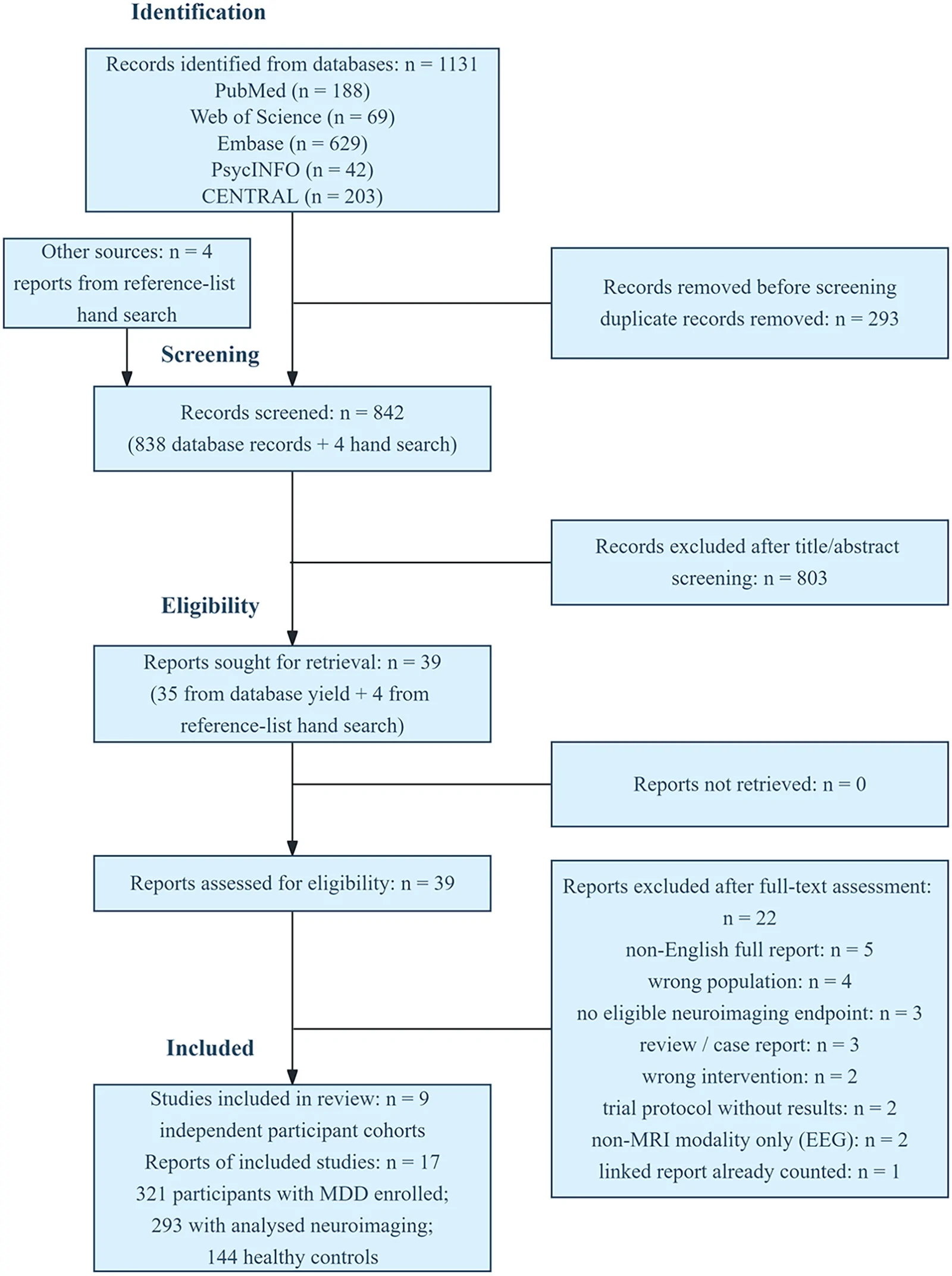
PRISMA 2020 flow diagram of the study selection process flowchart showing literature identification, screening, eligibility assessment, and inclusion of studies investigating neuroimaging changes after auricular vagus neuromodulation in major depressive disorder.

### 2.4 Data extraction

Two reviewers independently extracted data using a standardized form. Recorded variables included study design and participant characteristics, diagnostic criteria, stimulation protocols, neuroimaging modalities, analytical methods, affected brain regions and functional networks, clinical outcome measures, and associations between imaging findings and treatment response. Resting-state fMRI measures were recorded as reported: amplitude of low-frequency fluctuations (ALFF/fALFF) indexes the intensity of local spontaneous BOLD signal; regional homogeneity (ReHo), the local synchrony of neighboring voxels; functional connectivity density (FCD), the number of functional connections of each voxel at whole-brain level; seed-based functional connectivity (FC), the correlation of a seed region’s timecourse with all other voxels; independent component analysis (ICA) and functional network connectivity (FNC), spatiotemporal networks and their between-network coupling; graph-theoretical metrics and network-based statistics (NBS), connectome topology and connected subcomponents; and machine-learning analyses, multifeature predictive models. ALFF, ReHo, and FCD describe local activity, whereas seed FC, ICA/FNC, and NBS describe connectivity; the two classes can change in different directions and are not interchangeable. Task-fMRI BOLD activation and during-scan on/off stimulation-state FC were classified separately. Extraction differences were resolved by discussion and consensus.

Reports drawing on the same participant cohort were linked as described in §2.3, with participant denominators counted once per cohort. Inter-reviewer agreement for data extraction was high; the percentage agreement is reported in the **Supplementary Methods**. The complete effect-level coding matrix—recording for each effect its region, network attribution, direction relative to the stated comparator, comparator type, seed or whole-brain scope, multiplicity correction, and reported statistics—is provided as **Supplementary Table S3** (126 coded effects).

### 2.5 Quality assessment

Methodological quality (risk of bias) was appraised independently by two reviewers using the revised Joanna Briggs Institute (JBI) Critical Appraisal Tool for Quasi-Experimental Studies (non-randomized experimental studies) (25). No included study allocated participants to study groups at random: the eligible reports comprised sham-controlled sequential cohorts, non-randomized active-comparator trials, single-arm pre–post studies, and acute within-session designs, as well as one within-subject cross-over study in which only the order of the two active stimulation conditions was randomized. Because that randomization concerned condition order rather than participant allocation, the cross-over study does not constitute a randomized controlled trial; the authors themselves stated that their study was “not designed as a clinical trial.” The JBI randomized-controlled-trial checklist presupposes random allocation, and the prevalence and cross-sectional checklists are intended for non-interventional designs, so the quasi-experimental tool was the appropriate instrument. The revised tool comprises nine signaling questions spanning internal validity (temporal precedence; selection and allocation; confounding; intervention administration; outcome assessment, detection, and measurement; and participant retention) and statistical conclusion validity. Each item was answered “yes,” “no,” “unclear,” or “not applicable,” and appraisal was performed at the study level for each report’s primary neuroimaging outcome(s). “Not applicable” was used only where an item was logically inapplicable, such as differential co-interventions in single-group pre–post designs; the reliable-outcome-measurement item applies to every design, including single-arm studies, because standardized acquisition and processing are required regardless of whether a comparison group exists. For single-group pre–post comparisons, the participant-similarity item was answered “yes” per the JBI manual, as participants serve as their own controls; an independent control group was credited only where a separate group (sham arm, active-comparator arm, or healthy controls undergoing identical pre- and post-intervention measurements) was available, and not where healthy controls were measured at baseline only. Disagreements were resolved by consensus; inter-rater agreement for the item-by-item quality judgments is reported in the **Supplementary Methods**. No study was excluded based on quality; appraisal findings were used to weight and interpret the evidence. Item-level judgments and justifications for all 17 reports are provided in **Supplementary Table S4**.

The JBI prescribes no numerical cut-off scores for its appraisal tools and cautions against relying on aggregate scores alone; we therefore defined our grading rule *a priori* and report it transparently. For each report, the proportion of “yes” judgments among applicable items (excluding “not applicable”) was calculated, following the convention used in published JBI-based reviews in which ≥70% “yes” denotes high, 50–69% moderate, and <50% low methodological quality. This proportion was interpreted alongside five items judged critical for causal inference in this evidence base—control group (Q2), participant comparability/confounding (Q3), reliable outcome measurement (Q7), complete follow-up/retention (Q8), and appropriate statistical analysis including multiplicity control (Q9): reports with ≥70% “yes” and no “no” or “unclear” judgment on any critical item were graded high quality (low risk of bias); reports with 50–69% “yes,” or ≥70% “yes” with any “no”/”unclear” critical-item judgment, were graded moderate quality, with single-arm/within-subject studies lacking an independent control group (Q2 = “no” by design) capped at moderate; and reports with <50% “yes,” a “no” judgment on statistical conclusion validity (Q9), or “no” judgments on two or more critical items were graded low quality (high risk of bias).

### 2.6 Data synthesis

Given heterogeneity across studies in stimulation protocols, imaging modalities, analytical methods, and outcome measures, quantitative meta-analysis was not feasible and findings were synthesized qualitatively. Synthesis was performed by cohort and stratified across five non-interchangeable imaging paradigms, kept separate throughout: (1) longitudinal resting-state fMRI before and after 4–8 weeks of treatment; (2) acute single-session resting-state fMRI immediately before and after one stimulation session; (3) block-design stimulation fMRI during the first session, with stimulation-on blocks modeled against implicit baseline; (4) concurrent stimulation-state connectivity with on/off blocks during scanning; and (5) acute single-session task-based fMRI with stimulation delivered around a stress task (one cross-over respiratory-gated RAVANS study, in which both compared conditions were active parameter settings and all findings were acute/state-dependent). Key synthesis decisions were made *a priori* as follows.

#### 2.6.1 Network classification

Network attribution followed two rules, recorded separately for every effect. First, labels assigned by the original authors were retained and explicitly reported as the authors’ interpretation, introduced with the phrase “the authors interpreted.” Second, networks were attributed anatomically from peak coordinates using fixed *a priori* definitions: the default mode network comprised the precuneus/posterior cingulate cortex, medial prefrontal cortex, angular gyrus, middle temporal gyrus, and hippocampus; the salience network, the anterior insula and anterior/mid-cingulate cortex; the striatal–cortical reward circuit, the nucleus accumbens, caudate, putamen, globus pallidus, and ventral striatum with their medial, orbitofrontal, and ventrolateral prefrontal projections; and the frontoparietal/control network, the dorsolateral prefrontal cortex, middle frontal gyrus, and inferior parietal lobule. Regional signal change was never equated with network-level psychological function. Effects outside these four labels—including sensorimotor and visual networks, the hypothalamus, and the cerebellum—were retained under their original anatomical system rather than reassigned. Regional membership of the cortical network labels followed the Automated Anatomical Labeling atlas (AAL3) (26), chosen because it is the parcellation most frequently used by the included studies for region definition and seed placement, which maximizes correspondence with the primary reports; subcortical and brainstem labels (ventral striatum, nucleus accumbens, hypothalamus, and brainstem relay nuclei) followed the Harvard–Oxford subcortical structural atlas (27), which provides probabilistic anatomical boundaries in standard MNI space. All coordinates were expressed in MNI space before attribution, allowing consistent atlas-based assignment regardless of the parcellation used in the primary report.

#### 2.6.2 Directionality

Null findings (seeds, group contrasts, and *post-hoc* tests without significant treatment-related change) were recorded and are reported in parallel with positive findings. Because studies differed in seed regions, baseline comparisons, and statistical thresholds, the direction of change relative to the stated comparator was recorded for every effect; effects in opposite directions were never combined into a single “convergent” count, vote counts were direction-specific, and conflicts within or across reports are reported as inconsistencies with candidate methodological explanations. “Convergent” is used only for same-direction, multiplicity-corrected effects across independent cohorts.

#### 2.6.3 Acute versus long-term effects

Acute single-session and longitudinal studies were analyzed separately to distinguish immediate stimulation effects from sustained treatment-related changes; when a study reported both, each timepoint was described independently.

Overlapping cohorts. Linked reports from the same parent trial (§2.3) were treated as a single study unit: participant denominators and cohort-level vote counts were counted once per independent cohort, and overlapping results were not double-counted.

#### 2.6.4 Multiple imaging outcomes and analysis scope

All reported findings were included without selective extraction; when one study contributed results to more than one network category, each was recorded under the corresponding network, as some regional effects may reflect overlapping neural systems rather than independent observations. Studies using *a priori* seed regions or small-volume correction were recorded as ROI-restricted: their findings cannot speak to regions outside the search space, and vote-counting was performed only within the anatomical systems each study actually examined.

## 3 Results

### 3.1 Evidence base: linked reports, independent cohorts, and imaging paradigms

Seventeen neuroimaging reports met the eligibility criteria (**Figure 1**; **Table 1**). Audit of trial registrations, ethics approvals, and participant overlap showed that these reports arose from nine independent participant cohorts rather than seventeen independent samples. Three cohorts were organized under registered parent trials and each contributed linked analyses applying different metrics or stimulation timepoints to overlapping participants: Cohort C1 (parent trial ChiCTR-TRC-11001201; the parent clinical report, Rong et al., 2016 (6), enrolled 160 MDD patients, of whom the imaging subcohort enrolled 49), reported across five linked analyses (Fang et al., 2016 (28); Liu et al., 2016 (29); Fang et al., 2017 (30); Tu et al., 2018 (31); Wang et al., 2018 (32)), a single-blind, non-randomized sequential cohort with sham stimulation at the non-vagal ear margin, no healthy controls, analyzed subsets N = 34–38; Cohort C2 (ChiCTR-1800014277; ethics 2017-021-SQ), reported across three linked analyses (Ma et al., 2022 (33); Sun et al., 2023 (34); Sun et al., 2024 (35)), maximum 86 MDD and 54 controls; and Cohort C6 (ChiCTR2000029109; TECAS; ethics 2019-250-KY/YJKY2019052), reported across three linked analyses (Liao et al., 2023 (36); Ma et al., 2023 (37); Ma et al., 2024 (38)), maximum 51 MDD and 51 controls. The remaining cohorts were single-report studies: C3 (Chen et al., 2022 (39); 30 enrolled, 22 analyzed), C4 (He et al., 2023 (40); 22 MDD, 23 controls), C5 (Zhang et al., 2022 (41); NCT03607331; 15 MDD, 16 controls), C7 (Yi et al., 2022 (42); 20 analyzed), and C8 (Guo et al., 2024 (43); 19 analyzed), and C9 (Garcia et al., 2021 (44); 20 enrolled, 18 analyzed). Counting each cohort once, the independent evidence base comprised 321 enrolled MDD participants, 293 with analyzed imaging, and 144 healthy controls.

**Table 1:** Characteristics of the 17 included neuroimaging reports arising from nine independent participant cohorts (C1–C9).

Report	Cohort	Parent trial/registration	N analyzed (MDD/HC)	Intervention & parameters	Design & control	Imaging paradigm	Imaging metrics & analysis scope	Multiplicity correction (as reported)
Fang et al., 2016 (28), Biol Psychiatry	C1	ChiCTR-TRC-11001201 (parent clinical trial Rong 2016, n=160; imaging subcohort enrolled 49)	34 MDD (real 18/sham 16); 0 HC	taVNS 20 Hz, 4–6 mA, 30 min bid × 4 wk (auricular concha)	Single-blind, non-randomized sequential cohort; sham-controlled (non-vagal ear margin); no HC; not an RCT	Tier 1: longitudinal rs-fMRI (0/4 wk), 1.5T	ICA + dual regression (DMN); WB network-level	Z>2.6 + cluster p<.05 (>20 voxels); brain–symptom correlations uncorrected
Liu et al., 2016 (29), J Affect Disord	C1	ChiCTR-TRC-11001201	34 MDD (real 18/sham 16); 0 HC	Same as Fang 2016 (28)	Same C1 design; sham-controlled; no HC	Tier 1: longitudinal rs-fMRI (0/4 wk)	Bilateral amygdala (AAL) seed-based whole-brain FC; ROI seed + WB targets	voxel p<.005 uncorrected + cluster-level FWE p<.05 (>50 voxels)
Fang et al., 2017 (30), NeuroImage Clin	C1	ChiCTR-TRC-11001201	38 MDD (real 17/sham 21); 0 HC	taVNS 20 Hz; stimulation delivered during first session	Same C1 design; sham-controlled; no HC	Tier 3: block-design stimulation fMRI during first session (2 sessions × 3 × 30-s on blocks vs implicit baseline; GLM) — not a pre/post resting-state design	Task-block BOLD activation/deactivation; ROI-SVC over bilateral anterior insula/precuneus/thalamus/hippocampus; ROI + WB	One-sample voxel p<.001 + cluster FWE; between-group FWE small-volume correction
Tu et al., 2018 (31), Brain Stimul	C1	ChiCTR-TRC-11001201	Baseline 41 (real 20/sham 21); analyzed ≈37 [F(1,35)]; 0 HC	taVNS 20 Hz; 6-min continuous stimulation during scanning (on/off blocks, GSR)	Same C1 design; sham-controlled; no HC	Tier 4: concurrent stimulation-state FC (on/off blocks during scanning) — not pre/post-treatment resting-state	Medial & lateral hypothalamus seed whole-brain FC; 4 preset non-hypothalamic control seeds; ROI seed + WB	voxel p<.001 + cluster FDR p<.05
Wang et al., 2018 (32), J Psychiatr Res	C1	ChiCTR-TRC-11001201	37 MDD (real 17/sham 20); 0 HC	taVNS 20 Hz; continuous stimulation during scanning; frequency bands: typical 0.008–0.09/slow-5 0.01–0.027/slow-4 0.027–0.073 Hz	Same C1 design; sham-controlled; no HC	Tier 4: concurrent stimulation-state FC, frequency-stratified (on/off blocks)	Left/right nucleus accumbens (NAc) seed whole-brain FC; ROI seed + WB	voxel p<.005 + cluster FWE p<.05 (>50 voxels)
Ma et al., 2022 (33), Front Neurol	C2	ChiCTR-1800014277 (ethics 2017-021-SQ)	40 TRD + 40 HC	taVNS 4/20 Hz dense-dispersed wave (SDZ-IIB), single 30-min session	Acute controlled pre–post; HC received identical pre/post scans; no sham	Tier 2: acute single-session rs-fMRI	ReHo + right mOFC seed whole-brain FC; WB voxelwise (ReHo) + ROI seed FC	GRF voxel P<.01 + cluster P<.05 (two-sided); *post-hoc* Bonferroni P<.0125; covariates sex/age/education/FD
Sun et al., 2023 (34), J Affect Disord	C2	ChiCTR-1800014277 (ethics 2017-021-SQ)	55 MDD + 54 HC analyzed (58 MDD reported enrolled)	taVNS 20 Hz, 4–6 mA, single 30-min session (scan 3 min post-stimulation)	Acute single-arm pre/post + baseline-only HC; no sham	Tier 2: acute single-session rs-fMRI	ALFF + right precuneus seed (r=6 mm) whole-brain FC; WB voxelwise + ROI seed	GRF voxel P<.005 + cluster P<.05; clusters <60 voxels discarded
Sun et al., 2024 (35), Asian J Psychiatr	C2	ChiCTR-1800014277 (ethics 2017-021-SQ)	86 MDD enrolled; 77 modeled (9 motion exclusions); 57 with paired scans (42 responders/15 non-responders); 0 HC	taVNS 4/20 Hz dense-dispersed wave (SDZ-IIB), 30 min bid × 8 weeks	Single-arm longitudinal predictive modeling; no sham, no HC	Tier 1: longitudinal rs-fMRI (0/8 wk; baseline FC modeling)	Whole-brain AAL116 → 6,670 FC; elastic-net + nested LOOCV; 11/32 key features reported; WB data-driven FC	Feature selection P<.01; permutation testing 5,000; internal cross-validation framework
Chen et al., 2022 (39), J Tradit Chin Med	C3	No registration reported	30 enrolled → 22 analyzed MDD (recurrent MDD); 0 HC	taVNS 20 Hz × 8 weeks	Single-arm, uncontrolled; no HC, no sham	Tier 1: longitudinal rs-fMRI (0/8 wk)	Eight basal-ganglia seeds (bilateral NAc/globus pallidus/caudate/putamen) whole-brain FC; ΔFC–symptom correlations; ROI seeds + WB	GRF voxel p<.01 + cluster p<.05; clusters <15 voxels discarded
He et al., 2023 (40), Braz J Psychiatry	C4	No registration reported (grant CI2021A03405)	22 MDD (paired scans) + 23 HC (retest controls)	taVNS 4/20 Hz dense-dispersed wave (pulse width <1 ms), 30 min bid × 8 weeks	Single-arm longitudinal + HC test-retest control (ANCOVA); no sham	Tier 1: longitudinal rs-fMRI (0/8 wk)	ALFF/ReHo/FCD voxel-based (primary); VSi–precuneus & VSi–mOFC ROI-cluster FC; WB voxelwise + ROI	Two-way repeated-measures ANCOVA + GRF voxel p<.001 + cluster p<.05 (two-sided); HC retest mask (uncorrected p<.05)
Zhang et al., 2022 (41), Brain Sci 12:1730	C5	NCT03607331 (ClinicalTrials.gov, registered 2018-07-31)	16 enrolled → 15 MDD analyzed + 16 HC	taVNS 4/20 Hz dense-dispersed wave (SDZ-IIB; pulse 0.2 ms ±30%), 30 min bid, 5 d/wk × 8 weeks	Prospective exploratory single-arm + HC (HC scanned twice, no intervention); no sham	Tier 1: longitudinal rs-fMRI (0/8 wk)	Six bilateral striatal subregion seeds (Brainnetome: vCa/dCa/GP/NAc/dlPu/vmPu) whole-brain FC; ROI seeds + WB	Two-way mixed ANCOVA + GRF interaction voxel p<.001 + cluster p<.05 (two-sided); covariates sex/age/FD
Liao et al., 2023 (36), Brain Sci 13:274	C6	ChiCTR2000029109 (ethics YJ-KY2019052; TECAS program)	50 MDD (29 TECAS/21 escitalopram) + 49 HC; 5 dropouts	TECAS 4/20 Hz dense-dispersed wave (4 Hz 5 s/20 Hz 10 s alternating; SDZ-IIB), auricular + GV20 (Baihui) + EX-HN3 (Yintang), 30 min bid × 8 wk; comparator: escitalopram	Prospective, single-blind, non-randomized, active-comparator (escitalopram) + HC	Tier 1: longitudinal rs-fMRI (0/8 wk), 3.0T	Group-ICA: within-network connectivity & between-network FNC; WB ICA network-level	Within-network MDD-vs-HC: two-sample FWE p<.05 (mask one-sample FDR p<.05, >50 voxels); between-network FNC: uncorrected p<.05 only (figure legends explicit)
Ma et al., 2023 (37), Neurosci Lett 814:137414	C6	ChiCTR2000029109 (ethics 2019-250-KY)	51 MDD (34 TECAS/17 escitalopram) + 51 HC	TECAS 4/20 Hz × 8 wk vs escitalopram (parameters per Liao 2023 (36) protocol)	Non-randomized active-comparator + HC	Tier 1: longitudinal rs-fMRI (0/8 wk)	ALFF/ReHo + right medial superior frontal gyrus (mSFG) seed whole-brain FC; WB voxelwise + ROI seed	Repeated-measures 2×2 ANCOVA + GRF voxel P<.005 + cluster P<.05; covariates sex/age/education/FD
Ma et al., 2024 (38), Psychiatry Res Neuroimaging 339:111787	C6	Same TECAS program (ethics 2019-250-KY; ChiCTR2000029109); 34 TECAS patients = the 34 TECAS cases of Ma 2023 (37) (author-confirmed)	34 MDD (TECAS arm) + 34 matched HC	TECAS 4/20 Hz × 8 wk (single-arm report; no drug arm)	Single-arm pre/post + cross-sectional HC; no sham	Tier 1: longitudinal rs-fMRI (0/8 wk)	ALFF + left insula seed whole-brain FC; WB voxelwise + ROI seed	GRF voxel P<.005 + cluster P<.05
Yi et al., 2022 (42), Front Neurosci 16:1018387	C7	No registration reported (Second Xiangya Hospital, Central South University)	22 enrolled first-episode drug-naïve MDD → 20 analyzed (correlations n=18); 0 HC	taVNS 20 Hz, 4–6 mA, 30 min; first stimulation session + 4-wk home treatment (bid, ≥5 d/wk); MRI only at first session	Single-arm, uncontrolled (no HC, no sham); 4-wk clinical follow-up only (no wk-4 MRI)	Tier 2: acute single-session rs-fMRI (pre/post first 30-min stimulation)	ReHo whole-brain (paired t); WB voxelwise	Paired t + cluster-level FWE p<.05
Guo et al., 2024 (43), Brain Sci 14:945	C8	No registration reported (Beijing Hospital of Traditional Chinese Medicine, Capital Medical University)	19 MDD; 0 HC	taVNS × 4 weeks	Single-arm, uncontrolled (no HC, no sham)	Tier 1: longitudinal rs-fMRI (0/4 wk)	160-node FC matrix + graph theory (GRETNA, AUC over thresholds) + NBS; WB connectome-level	Global/node paired t with covariates age/sex/mean FD, FDR p<.05; NBS: 5,000 permutations, FWE p<.05
Garcia et al., 2021 (44), J Psychiatr Res	C9	NCT04467164	18 MDD analyzed (20 enrolled; paired sessions); 0 HC	RAVANS 30 Hz, 300 µs, 0.8-s bursts, ~30 min; e-RAVANS vs i-RAVANS, randomized order, two sessions within one week	Within-subject cross-over; i-RAVANS active parameter comparator; no sham, no HC	Tier 5: acute task-based fMRI (IAPS visual stress task; pre- and post-stimulation)	WB task activation (GLM) + seed-based FC (NTS/DRN/HYPO *a priori* seed ROIs); *a priori* ROI SVC	Task: voxel p<.001 + cluster FWE p<.05 (SVC); connectivity: cluster-corrected

1. Reports are ordered by cohort (C1–C9). Reports sharing a parent trial analyze overlapping participants with different imaging metrics or stimulation timepoints and are counted once; participant denominators are counted only once at the cohort level (321 enrolled MDD, 293 with analyzed imaging, 144 healthy controls).

2. N columns report participants included in the imaging analysis; enrolled/recruited numbers are given in parentheses where they differ (e.g., Fang et al., 2016 (28): 34 analyzed from 49 enrolled; Zhang et al., 2022 (41): 15 analyzed from 16 enrolled; Chen et al., 2022 (39): 22 analyzed from 30 enrolled).

3. Three interventions are distinguished: taVNS = transcutaneous auricular vagus nerve stimulation (auricular concha only); TECAS, transcutaneous electrical cranial–auricular acupoint stimulation, combining auricular taVNS with transcranial electrical stimulation at GV20 (Baihui) and EX-HN3 (Yintang), in trigeminal as well as vagal territories; TECAS was used by only one cohort (C6). RAVANS, respiratory-gated auricular vagal afferent nerve stimulation; e-RAVANS, expiratory-gated; i-RAVANS, inspiratory-gated.

4. Five non-interchangeable imaging paradigms: Tier 1 = longitudinal resting-state fMRI (pre/post 4–8 weeks); Tier 2 = acute single-session resting-state fMRI (immediate pre/post one 30-min stimulation); Tier 3 = block-design stimulation fMRI during the first session (stimulation-on blocks vs implicit baseline; Fang et al., 2017 (30) only); Tier 4 = concurrent stimulation-state connectivity with on/off blocks during scanning (Tu et al., 2018 (31); Wang et al., 2018 (32)); Tier 5 = acute single-session task-based fMRI with stimulation delivered around a stress task (Garcia et al., 2021 (44); C9 only; cross-over of two active RAVANS parameter settings, not a treatment trial).

5. Analysis scope: WB, whole-brain voxelwise exploratory analysis; ROI, *a priori* seed/region of interest with small-volume or cluster correction; ICA, independent component analysis network-level; graph/NBS, graph-theory/network-based connectome-level analysis.

6. GRF, Gaussian random field cluster-level correction; FWE, family-wise error correction; FDR, false discovery rate; SVC, small-volume correction. Correction details are as reported in the primary papers. Detailed findings including null results are in **Table 3** (Parts A and B); item-level JBI ratings are in **Supplementary Table S4**. For Garcia et al., 2021 (44) (C9), the methods text states cluster-level FDR P <.05 whereas the results tables are labeled FWE-corrected; we report as published.

Three stimulation approaches were distinguished: conventional taVNS (auricular concha only); TECAS, which combines auricular taVNS with transcranial electrical stimulation at cranial acupoints GV20 (Baihui) and EX-HN3 (Yintang), in trigeminal as well as vagal territories (only Cohort C6); and respiratory-gated auricular vagal afferent nerve stimulation (RAVANS), a respiratory-gated taVNS variant delivered to the cymba concha, in which expiratory- versus inspiratory-gated timing serves as an active parameter comparator (only Cohort C9; Garcia et al., 2021 (44); a cross-over experimental study, not a treatment trial). The imaging experiments fell into five non-interchangeable paradigms (tiers): (1) longitudinal resting-state fMRI before and after 4–8 weeks of treatment (seven cohorts); (2) acute single-session resting-state fMRI immediately before and after one 30-min stimulation (C2, C7); (3) block-design stimulation fMRI during the first session, with stimulation-on blocks modeled against implicit baseline (Fang et al., 2017 (30) only); (4) concurrent stimulation-state connectivity with on/off blocks during scanning (Tu et al., 2018 (31); Wang et al., 2018 (32)); and (5) acute single-session task-based fMRI with stimulation delivered around a stress task (Garcia et al., 2021 (44), C9 only; a cross-over comparison of expiratory- versus inspiratory-gated RAVANS, both active parameter settings; the authors explicitly state that the study was not designed as a clinical trial). Tiers 3, 4 and 5 are not pre/post-treatment resting-state studies and are synthesized separately; tier-5 evidence is acute and state-dependent. A given cohort could contribute reports to more than one tier, but its participants were counted once in all denominators. The strongest evidence came from group × time or between-group contrasts surviving FWE or GRF cluster-level correction; the between-network FNC analyses of Liao et al. (2023) (36) survived only uncorrected p <.05 (none survived FDR) and were treated as exploratory, as were most brain–symptom correlations. Methodological quality (JBI critical appraisal) is summarized in **Table 2**; grades indicate risk of bias within this non-randomized evidence base and do not constitute a formal evidence-certainty hierarchy.

**Table 2:** Methodological quality (risk of bias) of the 17 included reports.

Report	Cohort	Study design	Items met (yes/applicable)	Overall grade
Fang et al., 2016 (28)	C1	Single-blind non-randomized sequential cohort, sham-controlled (longitudinal rs-fMRI)	7/9	Moderate
Liu et al., 2016 (29)	C1	Single-blind non-randomized sequential cohort, sham-controlled (longitudinal rs-fMRI)	7/9	Moderate
Fang et al., 2017 (30)	C1	Single-blind non-randomized sequential cohort, sham-controlled (block-design fMRI, first session)	7/9	Moderate
Tu et al., 2018 (31)	C1	Single-blind non-randomized sequential cohort, sham-controlled (on/off stimulation-state FC)	7/9	Moderate
Wang et al., 2018 (32)	C1	Single-blind non-randomized sequential cohort, sham-controlled (on/off stimulation-state FC, frequency-resolved)	7/9	Moderate
Ma et al., 2022 (33)	C2	Acute controlled pre–post with healthy controls receiving identical pre/post scans	9/9	High
Sun et al., 2023 (34)	C2	Acute single-arm pre–post with baseline-only healthy controls	6/8	Moderate
Sun et al., 2024 (35)	C2	Single-arm 8-week longitudinal predictive-model study	7/8	Moderate
Chen et al., 2022 (39)	C3	Single-arm prospective pre–post study (no control)	7/8	Moderate
He et al., 2023 (40)	C4	Single-arm longitudinal study with healthy test–retest controls	8/9	Moderate
Zhang et al., 2022 (41)	C5	Prospective longitudinal study with healthy test–retest controls (NCT03607331)	9/9	High
Liao et al., 2023 (36)	C6	Prospective single-blind non-randomized active-comparator study (ICA; uncorrected between-network contrasts)	6/9	Low
Ma et al., 2023 (37)	C6	Non-randomized active-comparator quasi-experimental study	8/9	Moderate
Ma et al., 2024 (38)	C6	Single-arm longitudinal TECAS study with cross-sectional healthy controls	7/8	Moderate
Yi et al., 2022 (42)	C7	Single-arm acute pre–post study, drug-naïve first-episode MDD (no control)	7/8	Moderate
Guo et al., 2024 (43)	C8	Single-arm 4-week longitudinal graph-theory/NBS study (no control)	7/8	Moderate
Garcia et al., 2021 (44)	C9	Within-subject cross-over experimental study, randomized stimulation order (not a treatment RCT); i-RAVANS active parameter comparator, no sham, no HC (acute task-fMRI)	7/9	Moderate

1. Item-by-item ratings (yes/no/unclear/not applicable) with study-level justifications are provided in **Supplementary Table S4**. Grades follow the *a priori* rule defined in Methods (§2.5): high = ≥70% of applicable items met with no “no” or “unclear” judgment on the five critical items (control group, baseline comparability, reliable outcome measurement, complete follow-up, appropriate statistical analysis); moderate = 50–69% of items met, or ≥70% with a deficient critical item—single-group and within-subject studies without an independent control group are capped at moderate; low = <50% of items met, a “no” on statistical analysis, or “no” on two or more critical items. Denominators are the number of applicable items: 9 for studies with an independent control group (sham arm, active comparator, or healthy controls measured pre- and post-treatment) or a cross-over design, and 8 for single-group pre–post studies, for which the control-group item is not applicable.

2. No included study used random allocation of participants to study groups: all reports were non-randomized intervention studies (sequential cohort enrollment, non-randomized assignment to TECAS versus escitalopram, single-arm designs, or within-subject parameter cross-over). The single low-grade report also reported its between-network functional connectivity contrasts at uncorrected p <.05 only; those contrasts are treated as exploratory throughout the synthesis.

### 3.2 Methodological quality

Risk of bias was appraised for all 17 included reports (arising from 9 independent participant cohorts) with the revised JBI critical appraisal tool for quasi-experimental studies (**Supplementary Table S4**). Two reports (11.8%) were rated high quality (low risk of bias)—Ma et al., 2022 (33) (9/9 applicable items met) and Zhang et al., 2022 (41) (9/9)—both of which combined an independent comparison group measured at both time points, complete follow-up with explicit accounting of exclusions, and whole-brain inferences under Gaussian random field correction with prespecified covariates. Fourteen reports (82.4%) were rated moderate quality, and one report (5.9%)—Liao et al., 2023 (36)—was rated low quality (high risk of bias). At the cohort level, one cohort (C5) was high quality, six cohorts (C1, C3, C4, C7–C9) were moderate, C2—although it contained the high-rated Ma et al., 2022 (33) report—was classified moderate because its other two linked reports (Sun et al., 2023 (34); Sun et al., 2024 (35)) were moderate, and one cohort (C6) spanned moderate to low because one of its three linked reports (Liao et al., 2023 (36)) received a low rating; the two other reports from that cohort (Ma et al., 2023 (37); Ma et al., 2024 (38)) were moderate.

Several weaknesses recurred across the evidence base and temper the strength of causal inference. First, no included study used random allocation of participants to study groups: reports were non-randomized (sequential cohort enrollment, non-randomized assignment to TECAS versus escitalopram, or one within-subject cross-over in which only the order of two active stimulation conditions was randomized, or single-arm designs), so selection and allocation bias cannot be excluded; baseline between-group comparability could not be confirmed from the published texts for the five C1 reports and for the two active-comparator C6 reports (Liao et al., 2023 (36); Ma et al., 2023 (37)). Second, seven reports (Chen et al., 2022 (39); Sun et al., 2023 (34); Sun et al., 2024 (35); Ma et al., 2024 (38); Yi et al., 2022 (42); Guo et al., 2024 (43) and Garcia et al., 2021 (44)) lacked any independent control group for the pre–post treatment contrast (healthy controls, where present, were measured at baseline only), leaving natural fluctuation, regression to the mean, and expectancy effects uncontrolled; notably, the five C1 reports did include a sham-controlled arm, and Ma et al., 2022 (33) He et al., 2023 (40), and Zhang et al., 2022 (41) included healthy controls undergoing identical pre- and post-intervention measurements. Third, reporting of attrition was incomplete or unclear in eight reports (the five C1 reports, He et al., 2023 (40), Sun et al., 2023 (34), and Liao et al., 2023 (36)), where participant flow was described in aggregate but group-wise losses and their potential impact were not analyzed. Fourth, statistical rigor varied: whereas the primary imaging contrasts in 16 of the 17 reports were protected by cluster-level correction (GRF/Gaussian random field, FWE, or FDR; e.g., voxel P<.005–.01 with cluster P<.05, or cluster FWE/FDR), the headline between-network functional connectivity treatment effects in Liao et al., 2023 (36) were reported at an uncorrected threshold of P<.05, and the corresponding FDR-corrected between-network analyses were entirely null; that report was graded low quality accordingly. In addition, most brain–clinical correlation analyses across studies were not multiplicity-corrected and should be regarded as exploratory. Fifth, samples were small (MDD participants analyzed per report ranged from 15 to 77; Sun et al., 2024 (35) enrolled 86, of whom 77 were modeled after motion exclusions and 57 had paired pre–post scans); in total, 293 MDD and 144 healthy controls were analyzed across the 9 independent cohorts, precluding formal assessment of publication bias and limiting precision. Against these limitations, temporal precedence of intervention over outcome was unambiguous in every report, outcomes were measured with identical protocols and standardized processing pipelines within each study (all 17 reports met the “same way” and “reliable measurement” items), and null findings were increasingly reported in the more recent literature. Overall, the neuroimaging evidence is methodologically coherent in measurement but uniformly observational/quasi-experimental in design; network-level findings should therefore be interpreted as evidence of treatment-associated brain changes warranting confirmation in randomized, adequately powered, sham-controlled trials with pre-registered analysis plans.

### 3.3 Longitudinal resting-state fMRI (4–8 weeks)

Sham-controlled taVNS (C1). Fang et al. (2016) (28), using ICA dual-regression of the DMN (Z > 2.6, cluster p <.05), found group × time interactions in both directions: DMN coupling with regions including right anterior insula and parahippocampal gyrus decreased more after real than sham, whereas DMN coupling with left precuneus and lateral orbitofrontal cortex increased more after real; additional opercular, occipital, and medial-frontal clusters showed the reverse pattern; cluster-level DMN-connectivity correlations with HAMD change ran in both directions (uncorrected, exploratory). Liu et al. (2016) (29), using amygdala-seed FC (voxel p <.005, cluster FWE), found right amygdala–left DLPFC connectivity to increase after real (p = .031), decrease after sham (p = .012), and differ between groups (p = .011); the left amygdala seed yielded no significant cluster. Larger connectivity increases correlated with greater reductions in HAMD (r = −0.42), anxiety (r = −0.55), and retardation (r = −0.46) (exploratory).

Single-arm and control-comparator taVNS cohorts. Chen et al. (2022) (39) (C3; 22 patients with recurrent MDD, no controls) examined eight basal-ganglia seeds under GRF correction: bilateral nucleus accumbens seeds showed no significant clusters (P = .07) and bilateral putamen seeds were null (P >.05); globus pallidus seeds showed decreased FC with sensorimotor, inferior-parietal/precuneus, and visual regions, and the right caudate seed decreased FC with cerebellar/visual regions, with larger decreases correlating with greater symptom improvement (r up to −0.60; exploratory); normalization toward controls could not be tested. He et al. (2023) (40) (C4; 22 first-episode MDD, 23 twice-scanned untreated controls) found baseline lower cortical ALFF (dlPFC, OFC) and higher subcortical ALFF (ACC, dmPFC, ventral striatum, anterior thalamus, hippocampus, amygdala). Group × time interactions (GRF voxel p <.001) showed right ventral-striatal ALFF, ReHo, and FCD—hyperactive at baseline—decreasing toward control levels after treatment (F = 21.1/18.8/22.3), whereas precuneus/PCC clusters showed the opposite pattern, ALFF and ReHo increasing; controls showed no significant retest changes. Ventral-striatum–precuneus FC decreased (p = .048; exploratory) whereas an analogous control ventral-striatum–medial-OFC connection was unchanged (p = .934); larger ventral-striatal decreases correlated with higher remission rates (r = −0.763; r = −0.869; exploratory). Zhang et al. (2022) (41) (C5; 15 MDD, 16 controls), using six bilateral striatal subregion seeds with GRF-corrected interactions, found bidirectional normalization: hypoconnected striatal–prefrontal links (ventral caudate–ventrolateral PFC, NAc–dmPFC/vlPFC) increased after treatment, whereas hyperconnected striatal–visual/temporal links (to superior occipital, cuneus, middle temporal cortex) decreased; intra-striatal dorsal-caudate–putamen connectivity increased; correlations with HAMD change were exploratory (r = −0.586, −0.584, 0.629).

Guo et al. (2024) (43) (C8; 19 MDD, no controls) applied graph theory and NBS (FDR; 5,000-permutation FWE). Global efficiency increased and characteristic path length decreased (both p <.001), indicating greater global integration; gamma, lambda, sigma, and local efficiency showed no significant changes. Left angular gyrus degree centrality and nodal efficiency increased (FDR); all other nodes were null. An NBS component of 16 nodes and 10 edges—between-network connections the authors assigned to DMN–FPN, DMN–CON, and FPN–CON—decreased after treatment; topological changes correlated with HAMD-24 reduction (r = 0.616; r = −0.703; exploratory). Sun et al. (2024) (35) (C2; 86 enrolled, 77 modeled after motion exclusions, 57 with paired scans) used baseline whole-brain FC in an elastic-net model with nested leave-one-out cross-validation: internal cross-validated prediction of symptom reduction gave R = 0.66 (R² = 0.44) and responder classification AUC = 0.856 (accuracy 76.6%), with no external validation, sham arm, or controls; features concentrated in cortico-striato-pallido-thalamic, hippocampal/parahippocampal, and cerebellar connections; two vermis–hippocampal features were nonsignificant.

Active-controlled TECAS cohort (C6). Liao et al. (2023) (36) (50 MDD [29 TECAS, 21 escitalopram], 49 controls), using group-ICA, found lower DMN within-network connectivity in MDD at baseline (FWE-corrected). An insula-network group × time interaction and several time/group main effects reached FWE, but FDR-corrected *post-hoc* pre/post comparisons were null for all these networks, and between-network FNC showed no significant differences after FDR; at uncorrected p <.05 (presented as exploratory), differences involved DMN–DAN, DMN–RFPN, and visual–RFPN pairs, with post-TECAS and post-escitalopram directions largely opposite. Baseline medial-frontal/ACC connectivity correlated with baseline symptom severity (exploratory), while network changes showed no association with clinical change in either group. Ma et al. (2023) (37) (51 MDD [34 TECAS, 17 escitalopram], 51 controls) found lower baseline ALFF and ReHo in right medial superior frontal cortex (t = −5.07/−4.76; anatomically consistent with the DMN); medial-SFG-seed FC with occipital/temporal regions was elevated at baseline and decreased after treatment in both treatment groups (interactions P = .011–.017, GRF), whereas ALFF/ReHo group × time interactions yielded no significant clusters and no imaging–clinical correlations were reported. Ma et al. (2024) (38) (the 34 TECAS patients, 34 matched controls) found lower baseline left-insula ALFF (t = −4.30) and lower insula–prefrontal/ACC FC (discussed by the authors within an insula–DMN framework; the insula is anatomically consistent with a salience-network region); insula–ACC/orbitofrontal and insula–SFG/SMA connectivity increased after 8 weeks, and patients no longer differed significantly from controls post-treatment, though full normalization was not demonstrated (p = .075; p = .187; null); insula–ACC FC increase correlated with HAMD reduction (r = −0.34; exploratory). Clinically, response and remission rates did not differ significantly between TECAS and escitalopram in this small sample (HAMD response 62.9% vs 57.1%, P = .55; remission 29.4% vs 35.3%, P = .67); these null differences do not support equivalence or non-inferiority.

### 3.4 Acute single-session resting-state fMRI (immediate pre/post)

Ma et al. (2022) (33) (C2; 40 patients with treatment-resistant depression, 40 controls; one 30-min 4/20-Hz session) found a group × time interaction for ReHo in right medial orbitofrontal cortex (F = 18.06, GRF-corrected)—a region that the authors interpreted as part of the reward network: baseline ReHo was higher in patients and decreased toward control levels after stimulation, with no significant control change. Across-group time effects comprised ReHo decreases in cerebellar, inferior-temporal, orbitofrontal, and superior-frontal clusters and a precuneus increase; group main effects were null for both ReHo and seed FC. Right-medial-OFC seed FC with left inferior parietal cortex increased in patients after stimulation (F = 11.66), whereas a second OFC–supramarginal cluster showed a null patient *post-hoc* change (P = .113) despite a significant interaction; imaging changes showed no association with HAMD or HAMA change. Sun et al. (2023) (34) (C2; 55 MDD, 54 controls; one 20-Hz session) found baseline lower right-precuneus and higher right-angular-gyrus ALFF in MDD (DMN-anatomical regions); immediately after stimulation, right-precuneus ALFF decreased (per the abstract/results text; the sign printed in the results table is internally inconsistent) and precuneus-seed FC with left middle frontal cortex, PCC, and angular gyrus decreased (all GRF-corrected); baseline precuneus–PCC FC correlated with baseline HAMD (r = −0.288; exploratory). Yi et al. (2022) (42) (C7; 20 first-episode drug-naïve MDD, no controls) found ReHo to decrease immediately after the first session in seven clusters—bilateral mid-cingulate, left precentral/postcentral, right calcarine/lingual, left SMA, and paracentral lobule (cluster FWE)—with no cluster showing increased ReHo; MCC and SMA ReHo changes correlated with 4-week HAMD improvement (r = 0.62; r = 0.74; uncorrected, exploratory).

### 3.5 Block-design stimulation fMRI during the first session

Only Fang et al. (2017) (30)(C1; N = 38, real 17/sham 21) used this design (stimulation-on blocks vs implicit baseline; FWE). Real stimulation activated bilateral cerebellar crus I and left opercular/insular/precentral cortex and deactivated right medial frontal cortex; sham activated right middle/inferior frontal cortex, and sham-specific deactivations encompassed PCC, precuneus, hippocampus, and medial frontal cortex—DMN-anatomical deactivations observed under sham and not specific to taVNS. The real > sham contrast yielded a single surviving region, the left anterior insula (FWE small-volume correction); the reverse contrast produced no significant cluster and no brainstem/nucleus-of-the-solitary-tract activation was observed. Left-anterior-insula activation correlated with 4-week HAMD improvement in real (r = −0.65, p = .01) but not in sham (p = .6; exploratory).

### 3.6 Stimulation-state connectivity during scanning (on/off blocks)

Tu et al. (2018) (31) (C1; voxel p <.001, cluster FDR), using hypothalamic seeds during on/off blocks, found medial-hypothalamus connectivity with left rACC and right MFG to be lower, and with cerebellum higher, in real than sham during stimulation; the medial-hypothalamus–rACC on-vs-off change was significant in real (p = .004) but not sham (p = .18). This connection correlated with HAMD improvement in real during stimulation (r = 0.58, p = .02) but not in sham (p = .15), and showed no significant correlation in the resting (off) state; hypothalamus–MFG/cerebellar connections showing changes did not correlate clinically. Lateral-hypothalamus connectivity with MCC and putamen was lower in real than sham but did not correlate with outcome, and four preset non-hypothalamic control seeds yielded no significant clusters. Wang et al. (2018) (32) (C1; N = 37), using NAc seeds with frequency stratification (voxel p <.005, cluster FWE), found frequency-specific effects: in slow-5 (0.01–0.027 Hz), left-NAc connectivity with bilateral MPFC/rACC increased during stimulation in real (on vs off) and was higher in real than sham (Z = 5.07); slow-4/typical-band on/off effects were null in real, sham showed no change in any band, and slow-4 between-group effects were null. In the typical band, right-NAc connectivity with occipital, lingual/fusiform, and insular regions was higher in real than sham, while the reverse contrast was null; slow-5 NAc–prefrontal connectivity correlated with HAMD improvement in real only. The authors framed NAc-seed connections within a reward-circuit model; the MPFC/rACC targets are anatomically consistent with DMN/limbic regions.

### 3.7 Acute single-session task-based fMRI (stimulation around a stress task)

Garcia et al. (2021) (44) reported a cross-over experimental study in 20 enrolled premenopausal women (18 with complete paired sessions; NCT04467164) in which respiratory-gated transcutaneous auricular vagus nerve stimulation (RAVANS; cymba concha) was delivered in two active conditions in randomized order during block-design task fMRI. Unlike the resting-state paradigms synthesized in Sections 3.3–3.6, this study modeled stimulation-on blocks against an implicit baseline within the same scanning session, so all findings were state-dependent rather than reflecting pre–post treatment changes. Whole-brain analyses revealed connectivity changes that varied significantly across stimulation conditions, with the most consistent effects observed in regions receiving vagal afferent projections. Seed-based functional connectivity used *a priori* seed regions in the nucleus tractus solitarius (NTS) and dorsal raphe nucleus (DRN), the key vagal-afferent relay stations, with an additional hypothalamus (HYPO) seed; all were defined from the authors’ prior RAVANS studies rather than from an atlas. The reported patterns were anatomically consistent with the expected vagal afferent pathway, although direct activation of these brainstem nuclei was not measurable at the available imaging resolution. The methods text states cluster-level P <.05 corrected for false discovery rate, whereas the results tables are labeled family-wise error corrected; we report the findings as published.

### 3.8 Cross-paradigm synthesis and evidence grading

Vote-counting across independent cohorts (a qualitative tally, not a meta-analysis; **Table 3**) yielded four patterns. First, longitudinal striatal/corticostriatal changes—examined with ventral-striatal, NAc, caudate, globus-pallidus, and putamen seeds—appeared in four cohorts (C2 at feature level; C3; C4; C5), predominantly as bidirectional normalization (baseline hyperactivity/hyperconnectivity decreasing and baseline hypoconnectivity increasing toward control levels), although the null NAc/putamen seeds in C3 (P = .07) indicate seed/subregion dependence. Second, DMN-anatomical regions (precuneus, PCC, medial frontal cortex, angular gyrus, hippocampus) were implicated in seven cohorts, but direction was heterogeneous: local-activity metrics (ALFF, ReHo, degree centrality) mostly increased or normalized after treatment whereas connectivity metrics (seed FC, FNC, NBS) mostly decreased—no unidirectional DMN enhancement or suppression is supported. Third, the strongest between-group insula/salience evidence came from the first-session block design (left anterior insula, FWE-SVC), with supporting longitudinal seed-FC normalization; acute resting-state studies did not consistently detect insula effects. Fourth, acute resting-state changes were predominantly local-activity decreases, whereas stimulation-state effects were state-dependent and frequency-specific (slow-5 only) and cannot be pooled with longitudinal findings. Null results were pervasive and are reported in parallel throughout—null seeds and control connections, null group main effects, null FDR-corrected *post-hoc* and between-network ICA findings, null ALFF/ReHo interactions, and null imaging–clinical correlations and global graph metrics. **Table 3** summarizes the paradigm-stratified evidence, and **Supplementary Figure S1** presents the corresponding paradigm-stratified evidence map; mechanistic interpretation is developed as hypothesis in the Discussion.

**Table 3:** Synthesis of neuroimaging evidence for auricular vagus neuromodulation in major depressive disorder.

Part A. Paradigm-level synthesis.						
#	Evidence theme	Imaging paradigm (tier)	Cohorts reporting (cohort ID, reports)	Direction & pattern	Correction level of supporting effects	Consistency note (incl. null cohorts)
1	Striatal/corticostriatal “reward circuit” bidirectional normalization	Tier 1 — longitudinal resting-state (4–8 wk)	C4 (He 2023 (40), VSi ALFF/ReHo/FCD); C5 (Zhang 2022 (41), six striatal subregion seeds); C3 (Chen 2022 (39), basal-ganglia seeds); C2 (Sun 2024 (35), ML feature level)	Baseline hyperactivity/hyperconnectivity decreased and baseline hypoconnectivity increased toward HC levels after treatment (e.g., VSi hyperactivity ↓; vCa–vPFC & NAc–dmPFC/vlPFC ↑; vCa–SOG, GP–SOG, NAc–MTG, dCa–cuneus ↓)	He 2023 (40) & Zhang 2022 (41): GRF voxel p<.001/p<.005 + cluster p<.05 group×time interaction (strong); Chen 2022 (39): GRF voxel p<.01 + cluster p<.05 within-group; Sun 2024 (35): ML feature-level, internal CV	4 independent cohorts (C2 feature-level; C3; C4; C5), consistent normalization framing. Null: C3 bilateral NAc seeds P = .07 and bilateral putamen seeds P>.05 → strong seed/subregion dependence; C2 & C3 lack HC (normalization inferred for single-arm).
2	DMN local activity (ALFF/ReHo/degree) — predominantly increased/normalization after treatment	Tier 1 — longitudinal resting-state	C4 (He 2023 (40), precuneus/PCC ALFF & ReHo ↑ after tx); C8 (Guo 2024 (43), left angular gyrus degree centrality & nodal efficiency ↑); C6 (Ma 2023 (37), right mSFG ALFF/ReHo baseline ↓; Ma 2024 (38), insula-centered)	Local-activity metrics mostly increased or normalized after treatment (precuneus/PCC; angular gyrus node); medial-frontal baseline deficit documented cross-sectionally	He 2023 (40): GRF cluster-corrected interaction; Guo 2024 (43): FDR p<.05 (node metrics); Ma 2023 (37): GRF voxel p<.005 + cluster p<.05 (cross-sectional)	Local-activity direction contrasts with connectivity direction (see row 3). Null: Ma 2023 (37) ALFF/ReHo group×time interactions yielded no significant cluster (baseline cross-sectional differences only).
3	DMN/DMN-anchored connectivity — predominantly decreased after treatment or baseline-deficit normalization	Tier 1 — longitudinal resting-state	C1 (Fang 2016 (28), ICA-DMN dual regression); C3 (Chen 2022 (39), GP–precuneus/IPL ↓); C4 (He 2023 (40), VSi–precuneus ↓); C5 (Zhang 2022 (41), NAc–MTG & striatal–visual ↓); C8 (Guo 2024 (43), NBS DMN–FPN/DMN–CON/FPN–CON 10-edge component ↓); C6 (Liao 2023 (36) ICA; Ma 2023 (37) mSFG seed FC ↓; Ma 2024 (38) insula seed FC ↑ toward HC)	FC/FNC/NBS connectivity mostly decreased toward control levels after treatment or showed abnormal baseline connectivity normalizing bidirectionally; Fang 2016 (28) DMN interactions were bidirectional (some ↓, some ↑)	Fang 2016 (28): Z>2.6 + cluster p<.05; Chen/He/Zhang: GRF cluster-corrected; Guo 2024 (43): NBS 5,000-permutation FWE p<.05 (strong); Ma 2023 (37): GRF cluster-corrected interactions; Ma 2024 (38): GRF cluster; Liao 2023 (36) within-network: FWE p<.05	7 cohorts implicated DMN regions (C9 added as Tier 5 acute task-fMRI), but direction heterogeneous (local activity ↑ vs connectivity ↓). Null: Liao 2023 (36) FDR-corrected *post-hoc* pre/post and all between-network FNC were null (see row 9); Ma 2024 (38) post-treatment normalization tests p=.075/.187 (null).
4	Salience/insula-centered connectivity (longitudinal seed-based)	Tier 1 — longitudinal resting-state	C6 (Ma 2024 (38), left-insula seed FC: insula–ACC/OFC & insula–SFG/SMA ↑ after tx); C4 (He 2023 (40), ACC/dmPFC ALFF ↓); C1 (Liu 2016 (29), amygdala–DLPFC ↑; edge-anatomy limbic)	Insula-centered FC that was low at baseline increased toward HC levels after TECAS; ACC/dmPFC hyperactivity decreased	Ma 2024 (38): GRF voxel p<.005 + cluster p<.05; He 2023 (40): GRF cluster-corrected; Liu 2016 (29): voxel p<.005 + cluster FWE	Authors framed Ma 2024 (38) within an insula–DMN model; the insula is anatomically consistent with the salience network (network attribution dual-flagged). Correlations exploratory/uncorrected (insula–ACC r=-.338, P = .025).
5	Amygdala–prefrontal limbic FC (sham-controlled)	Tier 1 — longitudinal resting-state	C1 (Liu 2016 (29), bilateral amygdala seeds)	Right amygdala–left DLPFC FC ↑ after real taVNS (p=.031), ↓ after sham (p=.012), real > sham (p=.011); increase correlated with symptom reduction	voxel p<.005 (uncorrected) + cluster-level FWE p<.05; group contrast cluster-level corrected	Single cohort, single seed family. Null: left amygdala seed yielded no significant cluster; correlations (r=-.42/-.55/-.46) uncorrected/exploratory.
6	Sensorimotor & visual-network coupling with basal ganglia/striatum	Tier 1 — longitudinal resting-state	C3 (Chen 2022 (39), GP/caudate–postcentral, SMG, SPL, calcarine, lingual, cerebellum ↓); C5 (Zhang 2022 (41), striatal–SOG/cuneus ↓); C1 (Fang 2016 (28), occipital/opercular clusters, reverse-direction)	Decreased FC after treatment, consistent direction (↓) across cohorts	Chen 2022 (39): GRF voxel p<.01 + cluster p<.05; Zhang 2022 (41): GRF voxel p<.001 + cluster p<.05; Fang 2016 (28): Z>2.6 + cluster p<.05	3 cohorts (C1/C3/C5), direction consistently ↓; regions anatomically consistent with sensorimotor & visual networks (not author-labeled as such).
7	Whole-brain topology/connectome integration	Tier 1 — longitudinal resting-state	C8 (Guo 2024 (43), graph theory + NBS only)	Global efficiency ↑ & characteristic path length ↓ (greater global integration); left angular gyrus DC/NE ↑; NBS between-network component ↓	Global/node metrics: FDR p<.05; NBS: 5,000-permutation FWE p<.05 (strong within its design)	Single cohort (only graph/NBS study); no control group (single-arm, no sham). Null: gamma, lambda, sigma, local efficiency unchanged; all nodes except left angular gyrus null.
8	Machine-learning prediction of treatment response	Tier 1 — longitudinal resting-state (baseline features)	C2 (Sun 2024 (35), elastic-net + nested LOOCV, AAL116 → 6,670 FC)	Baseline FC predicted symptom reduction (R = .66, R²=.44) and responder classification (internal cross-validated AUC = 0.856, accuracy 76.6%, sensitivity 81.0%, specificity 71.4%); features in cortico-striato-pallido-thalamic, hippocampal/parahippocampal, cerebellar connections	Feature selection P<.01; permutation testing 5,000 times; nested internal LOOCV	Single cohort, single-arm, no external validation, no sham, no HC — an internal cross-validated predictive signature, not a clinical marker. Null: 2 vermis–hippocampus features non-significant (pallidum–hippocampus r=-.252 P = .059).
9	ICA within-/between-network FNC (TECAS vs escitalopram)	Tier 1 — longitudinal resting-state	C6 (Liao 2023 (36), group-ICA)	Baseline DMN within-network connectivity ↓ in MDD (FWE); insula-network group×time interaction & main effects FWE; between-network FNC differences (DMN–DAN, DMN–RFPN, PVN–RFPN) only at uncorrected p<.05, with TECAS vs escitalopram post-treatment directions largely opposite	Within-network: FWE p<.05 (mask one-sample FDR); between-network: uncorrected p<.05 only	Downgraded/exploratory: between-network FNC entirely null after FDR correction and not entered in the primary synthesis; Δ-network vs Δ-clinical correlations null in both arms.
10	Acute DMN/precuneus local activity & connectivity	Tier 2 — acute single-session resting-state	C2 (Sun 2023 (34), right precuneus ALFF & precuneus seed FC); C2 (Ma 2022 (33), precuneus ReHo time-effect ↑)	Right precuneus ALFF ↓ immediately post-session (abstract/results direction; results-table sign internally inconsistent); precuneus–MFG/PCC/angular-gyrus FC ↓ (GRF); Ma 2022 (33) precuneus ReHo ↑ time effect (heterogeneous)	Sun 2023 (34) & Ma 2022 (33): GRF voxel p<.005/p<.01 + cluster p<.05 (strong); correlations uncorrected	Single parent cohort (C2) for DMN acute effects; predominantly ↓ local/connectivity except Ma 2022 (33) precuneus ↑. C7 (Yi 2022 (42)) did not report DMN hubs.
11	Acute mOFC/reward-labeled region & frontoparietal coupling	Tier 2 — acute single-session resting-state	C2 (Ma 2022 (33), right mOFC ReHo + mOFC seed FC)	Right mOFC ReHo elevated at baseline in TRD and decreased toward HC levels after one session (interaction F = 18.06); mOFC–left inferior-parietal FC ↑ in patients (F = 11.66) — region the authors interpreted as part of the reward network	GRF voxel p<.01 + cluster p<.05 (bilateral); *post-hoc* Bonferroni P<.0125 (strong)	Single report (C2). Null: ReHo & FC group main effects null; second mOFC–supramarginal cluster patient *post-hoc* P = .113 (null); ReHo/FC changes showed no correlation with HAMD/HAMA.
12	Acute cingulate/sensorimotor local activity (ReHo)	Tier 2 — acute single-session resting-state	C7 (Yi 2022 (42), first-episode drug-naïve, ReHo)	ReHo ↓ immediately after first session in 7 clusters (bilateral MCC, precentral/postcentral, calcarine/lingual, SMA, paracentral lobule); no ↑ cluster	Paired t + cluster-level FWE p<.05 (strong)	Single cohort, single-arm (no HC, no sham). MCC/SMA anatomically consistent with salience/sensorimotor systems. Correlations (r=.62/.74) uncorrected/exploratory.
13	Left anterior insula activation (first-session block design) — strongest task-state between-group effect	Tier 3 — block-design stimulation fMRI (first session)	C1 (Fang 2017 (30) only)	Real stimulation activated bilateral cerebellar crus I & left opercular/insular/precentral cortex; real > sham: left anterior insula (FWE-SVC); activation magnitude correlated with 4-week HAMD improvement in real group only	One-sample voxel p<.001 + cluster FWE; between-group FWE small-volume correction (strongest task-state evidence)	Only block-design experiment. Null: reverse (sham > real) contrast no significant cluster; no brainstem/NTS activation observed; sham-group correlation p=.6 (null). PCC/precuneus/hippocampus deactivations were sham-specific (not taVNS effects).
14	Stimulation-state hypothalamic–limbic FC (state-dependent)	Tier 4 — concurrent stimulation-state FC (on/off blocks)	C1 (Tu 2018 (31), medial & lateral hypothalamus seeds)	During stimulation: medial-hypothalamus–rACC/MFG FC lower in real than sham (↓); medial-hypothalamus–cerebellum higher (↑); on-vs-off MH–rACC change real p=.004 vs sham p=.18; MH–rACC correlated with HAMD only during stimulation (r=.58, p=.02), not in resting (off) state	voxel p<.001 + cluster FDR p<.05 (strong)	CAN-related cohorts = 2 across paradigms (C1 Tier 4 + C9 Tier 5; paradigms not poolable). C1: state-dependent (effect absent off-stimulation). Null: lateral-hypothalamus–MCC/putamen & MH–MFG/cerebellum changes showed no clinical correlation; 4 preset non-hypothalamic control seeds yielded no significant clusters (specificity check).
15	Stimulation-state NAc–prefrontal FC, frequency-specific (slow-5)	Tier 4 — concurrent stimulation-state FC (on/off blocks)	C1 (Wang 2018 (32), left/right NAc seeds, frequency-stratified)	Slow-5 band (0.01–0.027 Hz): left NAc–bilateral MPFC/rACC FC ↑ during stimulation in real group and real > sham (Z = 5.07), correlated with HAMD improvement; typical-band right NAc–occipital/lingual/fusiform/insula FC real > sham	voxel p<.005 + cluster FWE p<.05, >50 voxels (strong)	Single cohort; frequency-specific (slow-5 only) — authors framed NAc seeds within a reward-circuit model; MPFC/rACC targets anatomically consistent with DMN/limbic regions. Null: slow-4 & typical-band on/off effects null in real; sham group null in all three bands; slow-4 between-group null; reverse contrast null.
16	Acute task-state sgACC/OFC/vmPFC activation during stress (RAVANS cross-over)	Tier 5 — acute task-based fMRI (IAPS visual stress task; within-subject cross-over)	C9 (Garcia 2021 (44), e-RAVANS vs i-RAVANS)	Task activation in sgACC/OFC/vmPFC increased after expiratory-gated relative to inspiratory-gated RAVANS (e-RAVANS post>pre vs i-RAVANS; Exh>Inh); 3 clusters: L sgACC 100 vox, L OFC 559 vox, L vmPFC 722 vox	SVC cluster FWE p<.05 (voxel p<.001 uncorrected). Peaks: L sgACC (−2,24,−8) T = 2.9 p=.032; L OFC (−14,30,−26) T = 4.1 p=.022; L vmPFC (−6,52,−14) T = 4.1 p=.027	Single cohort, within-subject cross-over (n=18 paired); i-RAVANS = active parameter comparator (no sham/HC); sgACC/OFC/vmPFC anatomically consistent with DMN/ventral limbic-autonomic regions. Second DMN-anatomical acute effect across the review.
17	Acute task-state central-autonomic-network connectivity: hypothalamus–DLPFC and NTS-seed FC (RAVANS)	Tier 5 — acute task-based fMRI (IAPS stress task; within-subject cross-over)	C9 (Garcia 2021 (44))	Post-stimulation stress-task period (Exh>Inh): hypothalamus seed → R dlPFC FC ↑ (878 vox); *a priori* NTS-seed ROI → LC FC ↑ (393 vox) and NTS → vmPFC FC ↑ (227 vox). During RAVANS administration runs: DRN seed → PCC FC, i-RAVANS > e-RAVANS (Inh>Exh, 418 vox); DRN → dlPFC FC, e-RAVANS > i-RAVANS (198 vox)	Cluster-corrected connectivity (Methods text states FDR-corrected p<.05; the original report’s results tables are labeled FWE-corrected — internal inconsistency reported as-is). Peaks: HYPO→R dlPFC (30,34,24) 878 vox T = 4.3 p<.001; NTS→LC L (−10,−32,−26) 393 vox T = 3.2 p=.007; NTS→vmPFC L (−10,54,0) 227 vox T = 3.9	CAN-related cohorts = 2 (C1 Tier 4; C9 Tier 5) — paradigms not poolable. Regions anatomically consistent with the central autonomic network. NTS/DRN/HYPO seeds: *a priori* functional ROIs from prior RAVANS fMRI studies (indirect localization). DLPFC counted as FC target within subcortical/brainstem finding. i-RAVANS session brain–behavior associations all null (see row 18).
18	Acute imaging–clinical state correlation during stress task (RAVANS)	Tier 5 — acute task-based fMRI (within-subject cross-over)	C9 (Garcia 2021 (44))	e-RAVANS session: greater task-state activation/FC change correlated with greater acute within-session BDI/STAI reduction (all β negative; GLM adjusted for baseline BDI and antidepressant use; df t(16), n=18); i-RAVANS session: no significant brain–behavior associations (all null)	GLM (df t(16)): sgACC vs ΔBDI β=−5.01 p=.007; OFC vs ΔBDI β=−6.64 p=.004; vmPFC vs ΔBDI β=−4.64 p=.007; NTS→LC vs ΔBDI β=−9.02 p=.001; NTS→vmPFC vs ΔBDI β=−8.48 p=.004. i-RAVANS session all null. HF-HRV results not coded (from linked abstract).	Single cohort (n=18 paired); consistent with the across-review pattern that acute imaging–clinical associations are state-dependent and largely exploratory; source imaging contrasts were cluster-corrected. No sham/HC reference; i-RAVANS (active comparator) yielded null.
19	Cross-paradigm pattern summary (vote-counting; not a meta-analysis)	Tiers 1–5	All cohorts (C1–C9) as detailed in rows 1–18	(i) Striatal/corticostriatal normalization in 4 cohorts (seed-dependent); (ii) DMN implicated in 7 cohorts but direction heterogeneous (local activity ↑ vs connectivity ↓); (iii) strongest insula/salience between-group evidence from Tier 3 (FWE-SVC); (iv) acute resting-state = local-activity ↓ vs stimulation-state = state-dependent/frequency-specific slow-5 effects — tiers not poolable	Strength stratification: FWE/GRF+cluster between-group interactions = strongest; Liao 2023 (36) between-network FNC = uncorrected (FDR-null) → exploratory; most brain–symptom correlations uncorrected → exploratory	Null results pervasive across all tiers and reported in parallel (null seeds: left amygdala, bilateral NAc/putamen, 4 non-hypothalamic control seeds, VSi–mOFC control connection; null group main effects; null FDR *post-hoc*/FNC; null ALFF/ReHo interactions; null normalization tests; null global graph metrics; absent imaging–clinical correlations in Ma 2022 (33)/Ma 2023 (37)).

Part B: Report-level principal findings, including explicitly null results						
Report (cohort)		N analyzed	Paradigm tier & comparator	Principal findings		JBI grade
Fang 2016 (28) (C1)		34 MDD (18 real/16 sham)	Tier 1; group×time (real vs sham), 0/4 wk	DMN group×time interactions bidirectional: DMN–right anterior insula/opercular FC ↑ in real, ↓ in sham (Z>2.6 cluster-corrected); precuneus/OFC clusters. Clinical: HAMD/SAS/SDS improved more after real than sham. Brain–symptom correlations uncorrected (exploratory).		Moderate
Liu 2016 (29) (C1)		34 MDD (18/16)	Tier 1; group×time (real vs sham)	Right amygdala–left DLPFC FC ↑ after real (p=.031), ↓ after sham (p=.012), real > sham (p=.011; z=3.72); ΔFC correlated with ΔHAMD r=-.42, anxiety r=-.55, retardation r=-.46 (exploratory). Null: left amygdala seed — no significant cluster.		Moderate
Fang 2017 (30) (C1)		38 MDD (17/21)	Tier 3; real vs sham block contrast, first session	Real: bilateral cerebellar crus I & left opercular/insular/precentral activation (one-sample FWE). Real > sham: left anterior insula (FWE-SVC); insula activation correlated with 4-wk HAMD improvement in real group. Null: sham > real contrast no cluster; no brainstem/NTS activation; sham-group correlation p=.6; PCC/precuneus/hippocampus deactivations sham-specific.		Moderate
Tu 2018 (31) (C1)		≈37 MDD (baseline 41)	Tier 4; on-vs-off stimulation, real vs sham	During stimulation: MH–left rACC & MH–right MFG FC lower in real than sham (p=.023/.002); MH–cerebellum higher. MH–rACC on-vs-off: real p=.004, sham p=.18 (null); MH–rACC correlated with HAMD only during stimulation (r=.58, p=.02). Null: MH–rACC resting-state correlation; 4 non-hypothalamic control seeds — no significant clusters; multiple secondary FC–clinical correlations absent.		Moderate
Wang 2018 (32) (C1)		37 MDD (17/20)	Tier 4; on-vs-off, frequency-stratified, real vs sham	Slow-5 (0.01–0.027 Hz): left NAc–bilateral MPFC/rACC FC ↑ during stimulation in real (on vs off z=4.07) and real > sham (Z = 5.07), correlated with symptom improvement; typical-band right NAc–occipital/lingual/fusiform/insula FC real > sham. Null: slow-4 and typical-band on/off effects in real; sham group null in all three bands; slow-4 between-group null; reverse contrast null.		Moderate
Ma 2022 (33) (C2)		40 TRD + 40 HC	Tier 2; group×time (TRD vs HC pre/post)	Right mOFC ReHo interaction (F = 18.06, P<.0001; region the authors interpreted as reward network): TRD baseline higher than HC (t=2.40), post-stimulation ↓ toward HC (t=-4.31), HC unchanged; mOFC–left inferior-parietal/angular FC interaction (F = 11.66). Null: ReHo & FC group main effects; second mOFC–supramarginal cluster patient *post-hoc* P = .113; ReHo/FC changes no correlation with HAMD/HAMA.		High
Sun 2023 (34) (C2)		55 MDD + 54 HC	Tier 2; within-group pre/post; baseline MDD vs HC	Baseline MDD vs HC: right precuneus ALFF ↓ (T=-2.881), right angular gyrus ALFF ↑ (T = 5.105). Post-session: right precuneus ALFF ↓ (abstract/results-text direction; results table prints T =+ 4.137 — internal sign inconsistency); precuneus seed FC with left MFG/PCC/angular gyrus ↓ (T=-4.94/-4.03/-4.97). Baseline precuneus–PCC FC vs baseline HAMD r=-.288, P = .032 (exploratory).		Moderate
Sun 2024 (35) (C2)		86 enrolled; 77 modeled; 57 paired	Tier 1; baseline FC prediction of 8-wk outcome (nested LOOCV)	Internal cross-validated prediction: symptom reduction R = .66, MAE = .15, R²=.44 (32-feature model R = .88); responder classification AUC = 0.856, accuracy 76.6%, sensitivity 81.0%, specificity 71.4%. Features concentrated in cortico-striato-pallido-thalamic, hippocampal/parahippocampal, cerebellar connections. No external validation; an exploratory predictive signature, not a clinical marker. Null: pallidum–hippocampus r=-.252 P = .059; two vermis–hippocampus features non-significant.		Moderate
Chen 2022 (39) (C3)		22 MDD (30 enrolled)	Tier 1; within-group 0/8 wk (no control)	Left globus pallidus FC ↓ with postcentral/inferior parietal; right globus pallidus FC ↓ with supramarginal/postcentral/superior parietal/inferior parietal/precuneus; right caudate FC ↓ with cerebellum/calcarine/lingual. Larger FC ↓ correlated with greater HAMD/SDS/SAS improvement (r=-.60/-.54/-.47/-.51; exploratory). Null: bilateral NAc seeds no significant cluster (P = .07); bilateral putamen seeds null (P>.05).		Moderate
He 2023 (40) (C4)		22 MDD + 23 HC	Tier 1; group×time ANCOVA with HC retest	Baseline MDD vs HC: cortical ALFF ↓ (dlPFC, OFC), subcortical ALFF ↑ (ACC, dmPFC, ventral striatum, anterior thalamus, hippocampus, amygdala). Group×time: right VSi (ventral caudate/NAc/anterior thalamus) ALFF/ReHo/FCD — hyperactive at baseline — ↓ toward HC (F = 21.08/18.81/22.34); precuneus/PCC ALFF & ReHo ↑ (F = 23.50/19.13); ACC/dmPFC/thalamic ALFF ↓ within patients. Null: HC retest no significant changes; control VSi–mOFC FC unchanged (t=-.057, p=.934). VSi–precuneus FC ↓ (t=2.10, p=.048; exploratory); larger VSi decreases correlated with higher remission (exploratory).		Moderate
Zhang 2022 (41) (C5)		15 MDD (16 enrolled) + 16 HC	Tier 1; group×time ANCOVA with HC retest	Bidirectional normalization (GRF interactions): hypoconnected links ↑ after treatment (vCa–ventrolateral PFC; NAc–dmPFC; NAc–vlPFC; intra-striatal dCa–dlPu); hyperconnected links ↓ (vCa–superior occipital; GP–SOG bilateral; NAc–middle temporal; dCa–cuneus). HC test-retest showed opposite/no treatment-like changes. ΔFC vs ΔHAMD: r=-.586 (vCa–SOG), r=-.584 (baseline NAc–dmPFC), r=.629 (dCa–cuneus) — exploratory.		High
Liao 2023 (36) (C6)		50 MDD (29 TECAS/21 drug) + 49 HC	Tier 1; within-network FWE; between-network uncorrected	Baseline MDD vs HC: DMN within-network connectivity ↓ (FWE; peak medial SFG). Insula-network group×time interaction & time/group main effects (insula, DAN, LFPN, visual, cerebellar) reached FWE — but FDR-corrected *post-hoc* pre/post comparisons null for all networks. Between-network FNC: no significant differences after FDR (null); uncorrected p<.05 exploratory patterns only (DMN–DAN, DMN–RFPN, PVN–RFPN), with TECAS vs escitalopram post-treatment directions largely opposite. Null: Δ-network vs Δ-clinical in both arms. Between-network findings treated as exploratory, not entered in the primary synthesis.		Low
Ma 2023 (37) (C6)		51 MDD (34 TECAS/17 drug) + 51 HC	Tier 1; 2×2 ANCOVA (TECAS vs escitalopram)	Baseline MDD vs HC: right mSFG (BA9) ALFF ↓ (t=-5.07) & ReHo ↓ (t=-4.76). mSFG-seed FC group×time interactions with right inferior occipital/bilateral middle temporal cortex (F interactions P = .011–.017): abnormally elevated FC decreased/normalized after treatment in both TECAS and escitalopram arms (time main effects P<.0001; group main effects ns); mSFG–left SFG time effect (peak t=5.28). Null: ALFF/ReHo group×time interactions — no significant cluster; no imaging–clinical correlations reported. Clinical: TECAS vs escitalopram response 62.9% vs 57.1% (P = .55), remission 29.4% vs 35.3% (P = .67) — null, no equivalence implication.		Moderate
Ma 2024 (38) (C6)		34 MDD (TECAS) + 34 HC	Tier 1; within-group 0/8 wk + cross-sectional HC	Baseline MDD vs HC: left insula (BA48) ALFF ↓ (t=-4.30); insula–right MFG/orbital SFG FC ↓ (t=-5.29) & insula–right ACC FC ↓ (t=-6.08) — authors discussed within insula–DMN framework; insula anatomically consistent with salience network. After 8 wk: insula–right ACC/orbital SFG FC ↑ (peak t=3.42) and insula–left SFG/SMA FC ↑ (peak t=4.34). Post-treatment patients vs HC no longer significantly different but normalization not demonstrated (p=.075; p=.187; null). Insula–ACC/SFG FC increase correlated with HAMD-17 reduction (r=-.338, P = .025; exploratory).		Moderate
Yi 2022 (42) (C7)		20 MDD (22 enrolled)	Tier 2; within-group pre/post first session	ReHo ↓ immediately post-stimulation in 7 clusters: bilateral MCC, left precentral/postcentral, right calcarine/lingual, left SMA, paracentral lobule (T -3.89 to -5.85); no cluster with increased ReHo. MCC/SMA anatomically consistent with salience/sensorimotor systems. ΔReHo (right MCC r=.62, p=.006; left SMA r=.74, p=.0005) correlated with 4-wk HAMD improvement (uncorrected — exploratory).		Moderate
Guo 2024 (43) (C8)		19 MDD	Tier 1; within-group 0/4 wk (no control)	Global: global efficiency ↑ (0.269→0.274, t=-4.54, p<.001) and characteristic path length ↓ (0.790→0.760, t=6.35, p<.001) — greater integration. Node: left angular gyrus degree centrality ↑ (18.4→25.3, t=-7.00) & nodal efficiency ↑ (t=-2.12, p=.048). NBS: 16-node/10-edge component of DMN–FPN, DMN–CON, FPN–CON between-network connections ↓ after treatment (FWE). ΔLp vs ΔHAMD-24 r=.616, p=.005; Δangular-gyrus DC vs ΔHAMD r=-.703, p=.001 (exploratory). Null: gamma, lambda, sigma, local efficiency unchanged; all other nodes null.		Moderate
Garcia 2021 (44) (C9)		18 MDD (paired)	Tier 5; within-subject cross-over (e-RAVANS vs i-RAVANS)	Task activation: sgACC/OFC/vmPFC ↑ after e-RAVANS vs i-RAVANS during stress task (SVC cluster FWE p<.05; voxel p<.001 uncorrected). Connectivity: hypothalamus→R dlPFC FC ↑; NTS-seed→LC and NTS→vmPFC FC ↑ (cluster-corrected). DRN→PCC i>e; DRN→dlPFC e>i during administration. Brain–behavior (e-RAVANS): sgACC/OFC/vmPFC and NTS-seed FC changes correlated with acute BDI/STAI reduction (β=−4.64 to −10.02, all p≤.012); i-RAVANS session all null. HF-HRV reported only in linked conference abstract, not coded here.		Moderate

1. The 17 included reports derive from 9 independent participant cohorts; reports sharing a parent trial (C1: ChiCTR-TRC-11001201, five linked analyses; C2: ChiCTR-1800014277, three linked analyses; C6: ChiCTR2000029109 [TECAS], three linked analyses) analyze overlapping participants with different imaging metrics or stimulation timepoints and are counted once; participant denominators are counted only once (321 enrolled MDD, 293 with analyzed imaging, 144 healthy controls).

2. Five non-interchangeable imaging paradigms are kept separate: Tier 1 longitudinal resting-state fMRI (pre/post 4–8 weeks); Tier 2 acute single-session resting-state fMRI (immediate pre/post one 30-min stimulation); Tier 3 block-design stimulation fMRI during the first session (stimulation-on blocks vs implicit baseline; Fang et al., 2017 (30) only); Tier 4 concurrent stimulation-state connectivity with on/off blocks during scanning (Tu et al., 2018 (31); Wang et al., 2018 (32)); Tier 5 = acute single-session task-based fMRI with stimulation delivered around a stress task (Garcia et al., 2021 (44)).

3. Network labels are attributed using two conventions: those explicitly stated by the original authors are reported verbatim; those inferred from peak-coordinate anatomy are described as “anatomically consistent with” the corresponding network. Regional activation is not equated with network-level mechanism.

4. Correction symbols: GRF, Gaussian random field cluster-level correction; FWE, family-wise error correction (SVC, small-volume correction over *a priori* regions); FDR, false discovery rate; cluster-level p values indicate corrected significance; CAN, central autonomic network; “uncorrected/exploratory” denotes effects reported only at uncorrected p<.05 (the Liao et al., 2023 (36) between-network FNC findings) or brain–symptom correlations without multiple-comparison correction.

5. null = the analysis was performed but no significant cluster/association was detected; null results are reported alongside positive effects and were not excluded from the synthesis.

6. Direction is reported relative to the stated comparator (group×time interaction, between-group, on-vs-off, or within-group pre/post); normalization denotes movement of baseline-deviant values toward healthy-control levels and is only asserted where a control comparison was made.

7. The AUC of 0.856 (Sun et al., 2024 (35); R² = 0.44) reflects internal nested leave-one-out cross-validation within a single-arm cohort without external validation and is an internal cross-validated exploratory predictive signature, not a clinical marker.

8. TECAS (combined transcranial cranial-acupoint + auricular stimulation, targeting trigeminal and vagal territories) is reported separately from auricular-only taVNS; response and remission rates did not differ significantly between TECAS and escitalopram (HAMD response 62.9% vs 57.1%, P = .55; remission 29.4% vs 35.3%, P = .67), and these null differences do not support equivalence or non-inferiority.

9. All coordinates are MNI; coordinates marked unverifiable in primary reports (e.g., Wang et al., 2018 (32) typical-band insular cluster) are flagged in **Supplementary Table S3**. Directional conflicts internal to individual primary reports (e.g., the Sun et al., 2023 (34) precuneus ALFF sign) are resolved toward the abstract/results-text direction and flagged for author clarification.

1. Full study characteristics (intervention parameters, design, imaging metrics, analysis scope, multiplicity correction) are in **Table 1**; JBI item-level ratings are in **Supplementary Table S4**.

2. Comparator abbreviations: group×time = treatment-group by time interaction; on-vs-off = stimulation-on versus stimulation-off blocks during scanning; within-group pre/post = single-arm pre-treatment versus post-treatment comparison; baseline MDD vs HC = cross-sectional patient–control comparison.

3. Direction is given relative to the stated comparator; ↑/↓ denote increase/decrease of the measured metric. “null” denotes analyses that were performed but yielded no significant cluster or association; the complete 126-row effect-level coding matrix (including 27 explicitly null rows) is provided in **Supplementary Table S3**.

4. Findings from Tiers 2–5 are not pooled with Tier 1 longitudinal findings in any vote count; vote-counting was across independent cohorts and cannot estimate pooled effect sizes (meta-analysis not feasible; see Methods).

## 4 Discussion

### 4.1 Principal findings

This review synthesized neuroimaging evidence from 17 reports arising from 9 independent participant cohorts (321 enrolled patients with MDD, of whom 293 entered imaging analyses, together with 144 healthy controls; participant denominators were counted once at the cohort level). Because stimulation timepoints, imaging tasks, and analysis levels differed markedly across reports, findings were organized by cohort and by five non-interchangeable imaging paradigms (Sections 3.1 and 3.3–3.8) rather than pooled; no coordinate-based meta-analysis was attempted, and synthesis relied on cohort-level vote-counting.

At the cohort level, the most reproducible pattern involved striatal–cortical regions. We use this term anatomically, to denote the nucleus accumbens (NAc), ventral and dorsal striatum (caudate, putamen, globus pallidus, ventral striatum [VSi]), and their projections to medial and orbitofrontal prefrontal cortices (mOFC; vmPFC/dmPFC; vlPFC), a distributed system anatomically consistent with the corticostriatal reward circuit. Across four independent cohorts (C2–C5), findings in these regions consistently showed bidirectional normalization: values that were abnormally high at baseline decreased after treatment, whereas values that were abnormally low increased, with post-treatment values moving toward those of healthy controls. In He et al. (2023) (40), VSi ALFF, ReHo, and functional connectivity density were elevated at baseline and decreased after 8 weeks of taVNS (group × time interaction, GRF-corrected), whereas healthy controls scanned twice over the same interval showed no significant change. In Zhang et al. (2022) (41), striatal–prefrontal connectivity that was low at baseline (ventral caudate–ventrolateral PFC; NAc–dmPFC/vlPFC) increased after treatment, whereas abnormally high striatal–visual connectivity (superior occipital gyrus; cuneus) decreased; within-striatum connectivity (dorsal caudate–dorsolateral putamen) increased. In Chen et al. (2022) (39), globus pallidus– and caudate-seeded connectivity to sensorimotor and visual cortices decreased after 8 weeks, whereas the bilateral NAc seeds yielded no significant clusters (P = .07) and the bilateral putamen seeds were likewise null (P >.05). Beyond these longitudinal effects, two additional analyses from the C2 cohort are noteworthy: an acute experiment examining normalization of elevated mOFC regional homogeneity within a single 30-min session in treatment-resistant depression (Ma et al., 2022) (33), and a longitudinal machine-learning analysis reporting that baseline connectivity features enriched in the corticostriatal–pallidal–thalamic circuit, hippocampus, and cerebellum predicted subsequent symptom change (Sun et al., 2024 (35); evidence-strength qualification in Section 4.4).

Regions of the default mode network (DMN), including the precuneus/PCC, medial prefrontal cortex, angular gyrus, middle temporal gyrus, and hippocampus, were involved in 7 of 9 cohorts, but with markedly heterogeneous directions. Local activity metrics (ALFF, ReHo, degree centrality) mostly increased or normalized (precuneus clusters in He et al., 2023 (40); left angular gyrus nodal degree and efficiency in Guo et al., 2024 (43)), whereas connectivity metrics (seed-based FC, ICA, NBS) mostly decreased (bidirectional DMN dual-regression changes in Fang et al., 2016 (28); VSi–precuneus FC in He et al., 2023 (40); an NBS component spanning DMN–frontoparietal and DMN–cingulo-opercular connections in Guo et al., 2024 (43); baseline DMN within-network connectivity in Liao et al., 2023 (36)). The direction of precuneus findings was inconsistent across reports: precuneus ALFF was reported as decreased in Sun et al. (2023) (34) whereas a time-effect increase in precuneus ReHo was reported in Ma et al. (2022) (33). A unidirectional account of DMN involvement is therefore not supported.

The anterior insula was the only regional target that survived between-group correction in a task/stimulation-state paradigm: in Fang et al. (2017) (30), left anterior insula activation during stimulation blocks was greater with real than with sham stimulation (FWE small-volume correction) and correlated with 4-week symptom improvement in the real-stimulation group only. Longitudinal insula-seed findings (Ma et al., 2024) (38) showed within-group increases in insula–ACC/orbitofrontal connectivity after 8 weeks of TECAS, but post-treatment values did not differ significantly from controls (P = .075 and P = .187), so formal normalization was not demonstrated. At the whole-brain network level, one graph-theory cohort (Guo et al., 2024 (43)) reported increased global efficiency, decreased characteristic path length, and increased left angular gyrus nodal degree and efficiency (FDR-corrected), with other global metrics null. Finally, in C1, amygdala–DLPFC connectivity increased after 4 weeks relative to sham (Liu et al., 2016 (29), cluster-level FWE), while the left-amygdala seed analysis was null.

### 4.2 Putative neurobiological mechanisms

All circuit-level interpretations in this section are hypotheses generated by the synthesis (**Figure 2**); none was tested causally by the included studies, and network labels were assigned *post hoc* rather than specified in advance.

**Figure 2:**
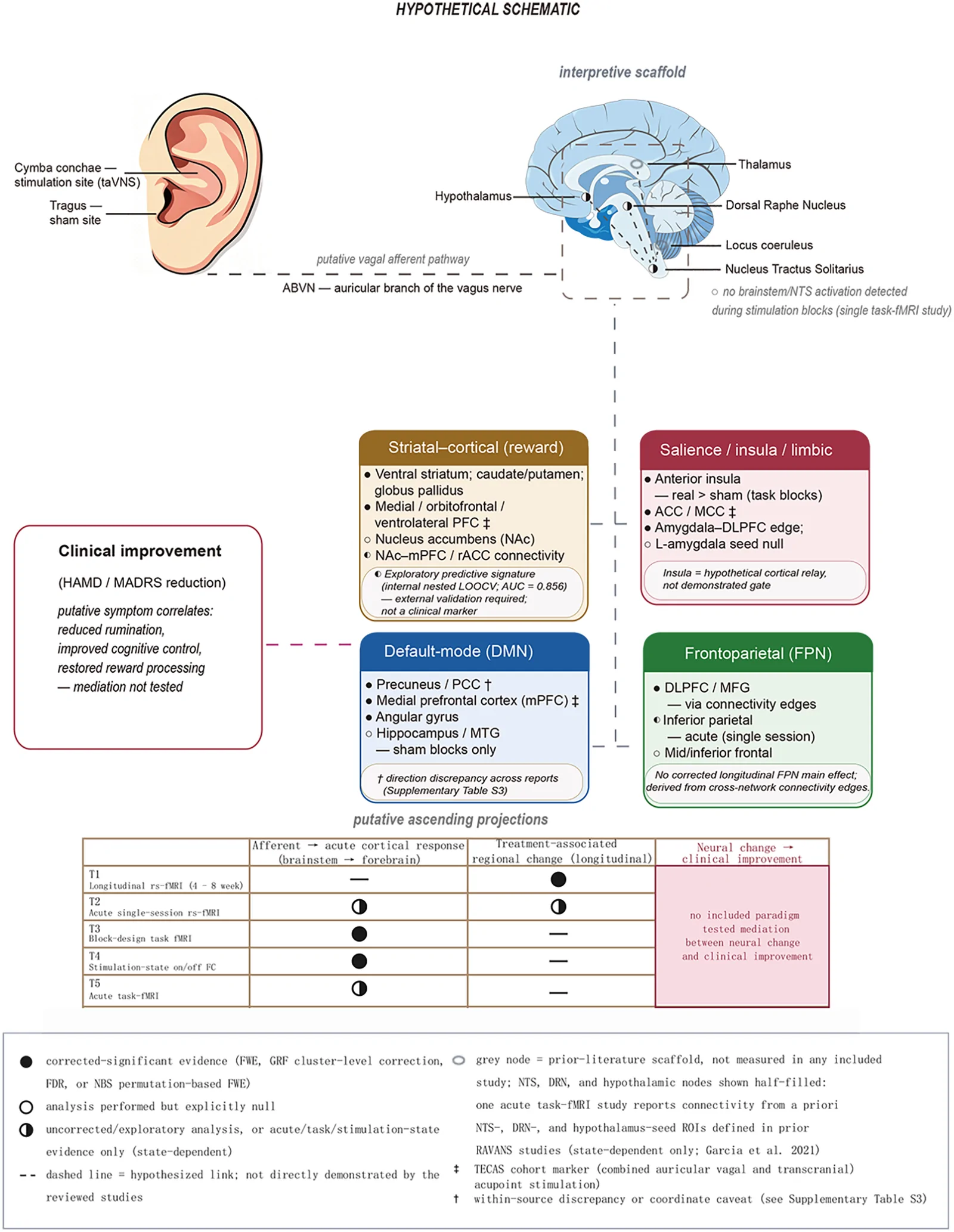
Proposed network-level neurobiological framework underlying the antidepressant effects of auricular vagus neuromodulation in major depressive disorder. This strictly hypothetical schematic summarizes the afferent-pathway and network-level hypotheses developed in Section 4.2; symbols are defined in the in-figure legend, and dashed links denote hypothesized rather than demonstrated pathways.

The afferent pathway most often invoked to explain central effects of auricular stimulation runs from auricular vagal afferents to the nucleus tractus solitarius (NTS), and thence to the locus coeruleus, raphe nuclei, thalamus, hypothalamus, and forebrain structures; this account derives from anatomical tracing, invasive vagus nerve stimulation, and transcutaneous stimulation imaging studies (45–49). No included study measured brainstem nuclei directly, and Fang et al. (2017) (30) explicitly reported that no NTS activation was detected during stimulation blocks. Garcia et al. (2021) (44) employed *a priori* seed regions in the nucleus tractus solitarius (NTS) and dorsal raphe nucleus (DRN), derived from the authors’ prior RAVANS studies and reported state-dependent connectivity changes anatomically consistent with the expected vagal afferent pathway, but direct NTS activation was likewise not measurable at the available resolution. The ascending pathway should therefore be read as an interpretive scaffold drawn from prior literature rather than as a pathway demonstrated in the reviewed studies. Furthermore, because TECAS combines auricular stimulation with transcranial electrical stimulation at cranial acupoints in trigeminal as well as vagal territories (Section 3.1), findings from the TECAS cohort cannot be attributed specifically to vagal afferent activation.

A first set of hypotheses concerns the default mode and salience systems. DMN-related changes could be hypothesized to reflect altered self-referential processing or rumination (14, 50, 51), but the opposite directions observed for local-activity versus connectivity metrics (Section 4.1) preclude any coherent directional claim. Acute engagement of the left anterior insula in the block-design experiment (30) is anatomically consistent with known cortical projection targets of vagal afferents and with the role of salience-network regions in interoception and affective-state switching (14); it supports a hypothetical early cortical relay, but pertains exclusively to stimulation-state and task paradigms and cannot be generalized to longitudinal treatment effects.

A second hypothesis concerns reward and motivation circuitry. The striatal–cortical normalization pattern is anatomically consistent with putative involvement of corticostriatal systems implicated in anhedonia and motivated behavior (52); several author teams interpreted their findings in these terms, and a link between striatal changes and anhedonia remains a plausible hypothesis. This interpretation is qualified by the null bilateral NAc-seed (P = .07) and putamen-seed (P >.05) results in Chen et al. (2022) (39), which indicate that conclusions depend on striatal subregion and seed choice; moreover, imaging changes were observed alongside symptomatic improvement but were not shown to mediate it.

A third hypothesis concerns cognitive-control circuitry. Frontoparietal findings generate the hypothesis that treatment-associated changes extend to systems supporting attention and executive function, domains that are broadly impaired in MDD (53, 54). This is the least substantiated of the regional hypotheses: frontoparietal involvement appeared in only three cohorts, and the between-network functional connectivity results of Liao et al. (2023) (36) were significant only at uncorrected thresholds and were entirely null after FDR correction.

Finally, the single-cohort graph-theory results (43) generate the hypothesis of enhanced global network integration with reduction of abnormally elevated between-network connectivity, but this requires independent replication. Across all four hypotheses, two cautions apply. First, describing a cluster as belonging to the “DMN” or the “reward circuit” is an anatomical attribution; reverse inference from regional signal change to psychological function is not licensed by these data (19). Second, the observations establish treatment-associated brain changes, not causal pathways; confirmation requires randomized, adequately powered, sham-controlled trials with preregistered network-level endpoints.

### 4.3 Clinical implications

From a clinical perspective, the reviewed evidence shows that vagus nerve stimulation (including TECAS) is accompanied by distributed, treatment-associated brain changes rather than effects confined to a single region, and that these changes occur in systems repeatedly implicated in the symptom dimensions of major depressive disorder. This pattern is consistent with the view of MDD as a disorder of large-scale network dysfunction rather than focal regional abnormality (12, 14), and provides a neurobiological rationale for continued clinical evaluation of auricular stimulation. The present data do not, however, establish that clinical benefit is produced by any particular network change; imaging changes were observed alongside symptomatic improvement, and mediation was not tested.

The distributed anatomical pattern may also help frame the heterogeneity of clinical response. Depressive symptoms span emotional regulation, reward processing, and cognitive control rather than dysfunction within a single domain (14, 53), and the regional findings reviewed above correspond to these domains only at the level of anatomical attribution. Whether changes in particular systems track improvement in particular symptom dimensions remains an open question: most imaging–clinical correlations were uncorrected and are regarded as exploratory, and no study performed a formal mediation analysis. Longitudinal studies with preregistered symptom-domain endpoints are needed before neural changes can be used to explain interindividual differences in response.

With respect to clinical translation, no imaging feature in the reviewed evidence is ready for clinical use. One study reported a baseline connectivity model that discriminated subsequent treatment responders with an AUC of 0.856 under internal nested cross-validation (35); because the model was developed within a single-arm design and evaluated only through internal cross-validation, it constitutes an exploratory predictive signature that requires external validation in independent, preferably sham-controlled, cohorts (35, 55, 56). Candidate imaging features may in future complement symptom-based assessment for patient selection and treatment monitoring, but such applications remain hypothetical and should not inform clinical decision-making until prospectively validated in multicenter studies.

### 4.4 Limitations

Several limitations qualify the interpretation of this synthesis and should be read alongside the paradigm-stratified Results (Sections 3.1 and 3.3–3.8) and the cohort map in **Table 1**. First, the evidence base is small and interdependent. The 17 reports arose from 9 independent cohorts; analyzed cohort sizes ranged from 15 to 86 MDD participants, and four cohorts (C1, C3, C7, C8) included no healthy controls, so baseline abnormality and treatment-associated normalization could not be tested in those cohorts. Participant denominators were therefore counted once at the cohort level: linked reports within the three registered parent trials (C1, C2, C6) applied different imaging metrics or stimulation timepoints to overlapping participants and do not constitute independent replications (6, 35, 36). Most cohorts were enrolled at one or a small number of collaborating centers, and all came from a small number of collaborating research groups, which further limits the independence and generalizability of the observed patterns.

Second, the five imaging paradigms are not interchangeable. Longitudinal resting-state scans before and after 4–8 weeks of treatment, acute resting-state scans immediately around a single 30-minute session, block-design task fMRI during the first stimulation session (30), and connectivity measured during continuous on/off stimulation in the scanner (31, 32) address different questions and cannot be pooled as one resting-state finding. Stimulation-state effects were also state- and frequency-specific: effects in the NAc-seed experiment were confined to the slow-5 band (0.01–0.027 Hz), with slow-4 and typical-band within-group analyses and all sham-group analyses null (32). Coordinates, seeds, stimulation protocols, acquisition parameters, and analysis pipelines likewise varied across reports. These differences precluded the prespecified quantitative meta-analysis described in the Methods; cohort-level vote-counting can rank reproducibility across cohorts but cannot estimate pooled effect sizes or quantify between-study heterogeneity. With only nine independent cohorts, formal tests for publication bias or small-study effects were not feasible, and selective reporting of positive regional findings cannot be excluded in a literature dominated by small, exploratory neuroimaging studies with substantial analytical flexibility (57, 58). Finally, although no language filter was applied during the five-database search, eligibility was restricted to English-language reports; non-English records entered the screening pool and were excluded at screening, so relevant evidence published in other languages may have been missed.

Third, explicitly null analyses were common and are reported in parallel in **Table 3** (Parts A and B), rather than treated as absences of evidence. The effect-level coding matrix compiled for this review contains 126 coded effect rows, including 27 explicitly null effect rows (**Supplementary Table S3**), concentrated in sham or control contrasts, particular seed choices, corrected network-level analyses, and absent imaging–clinical correlations. Examples include null findings for all frequency bands in the sham group of the NAc stimulation-state study (32); a null resting-state imaging–clinical correlation and four null non-hypothalamic control seeds in the hypothalamic stimulation-state study (31); no significant retest changes among healthy controls and an unchanged ventral-striatum–medial-OFC control connection (P = .934) (40); null bilateral NAc (P = .07) and putamen seeds (P >.05) (39); a null left-amygdala seed (29); null FDR-corrected *post-hoc* and between-network ICA comparisons (36); no significant changes in non-nodal global graph metrics (43); and absent or null imaging–clinical correlations in several cohorts, including the TECAS ICA report and the acute treatment-resistant-depression report (33, 36). In addition, insula–prefrontal/ACC connectivity values after 8 weeks of TECAS did not differ significantly from control values at conventional thresholds (P = .075 and P = .187), so formal normalization was not demonstrated (38). Most brain–symptom correlations were not protected by multiplicity correction and should be considered exploratory (57, 58); the uncorrected between-network FNC findings in the TECAS ICA report are likewise exploratory and were excluded from the main synthesis (36).

Fourth, the only machine-learning prediction model remains exploratory. Sun et al. (2024) (35) reported R² = 0.44 and responder-classification AUC = 0.856, but these estimates came solely from internal nested leave-one-out cross-validation in a single cohort; there was no independent test cohort, external validation, sham arm, or prospective clinical evaluation. The resulting connectivity pattern is therefore an exploratory predictive signature, not a clinical marker, and model performance may be optimistic in a field where small samples and analytical flexibility are known to inflate brain-wide associations (23, 57, 58). Independent replication in adequately powered, sham-controlled cohorts is required before any imaging feature can inform treatment selection (35, 56).

Fifth, causal attribution is constrained by design and by the intervention itself. No included study used randomized allocation: the C1 parent investigation was a single-blind, non-randomized sequential cohort study with a sham control (6), and the TECAS cohort used non-randomized assignment to TECAS versus escitalopram (36). Selection, allocation, and expectancy effects therefore cannot be excluded. The credibility of sham stimulation is also uncertain because auricular stimulation produces cutaneous tingling and somatosensory sensations that may reveal group assignment and influence both neural measurements and self-reported symptoms (6). No study tested mediation, so treatment-associated imaging changes were observed alongside clinical improvement but were not shown to mediate it. Moreover, TECAS combines auricular stimulation with cranial acupoints (GV20 Baihui; EX-HN3 Yintang) in trigeminal as well as vagal territories; findings from that single cohort therefore cannot be attributed to auricular vagal afferents alone (36, 38). Clinically, response and remission rates did not differ significantly between TECAS and escitalopram in this small sample (HAMD response 62.9% vs 57.1%, P = .55; remission 29.4% vs 35.3%, P = .67); these null differences do not support equivalence or non-inferiority (36, 37).

Sixth, measurement and participant-level confounds were controlled inconsistently. Although several protocols required medication washout or recruited drug-naïve participants, concurrent psychotropic medication was not excluded in every study; medication exposure, head motion, cardiac and respiratory noise, scanner and preprocessing differences, and individual variation in auricular vagal innervation and ear anatomy may each affect resting-state BOLD measures. Motion exclusions were explicitly reported in some cohorts but not uniformly, and physiological-noise regression varied across pipelines (57, 58). The evidence base was also restricted to magnetic resonance neuroimaging, so it does not cover electroencephalographic or other modalities. These factors do not invalidate the cohort-level patterns, but they limit precision, cross-study comparability, and the specificity of any anatomical or psychological interpretation.

These limitations define the appropriate agenda for future work. Confirmatory studies should use randomized, sham-controlled, multi-center designs with adequate sample sizes; preregistered imaging endpoints and analysis plans; harmonized stimulation reporting and acquisition parameters (10, 38); explicit reporting of null analyses; and independent, external validation of candidate predictive signatures (35, 56). Designs should also separate acute stimulation-state effects from longitudinal treatment effects, include healthy controls scanned at both timepoints where normalization claims are intended, and test mediation rather than assuming that concurrent clinical improvement explains regional signal change. Until such evidence is available, the reviewed findings should be read as hypothesis-generating evidence of treatment-associated brain changes, not as causal pathways or clinically actionable imaging markers.

## 5 Conclusion

This systematic review synthesized neuroimaging evidence from 17 reports arising from 9 independent cohorts of patients with MDD (321 enrolled patients, 293 included in imaging analyses, and 144 healthy controls, with participant denominators counted once at the cohort level). The most reproducible pattern was bidirectional normalization in striatal–cortical regions anatomically consistent with the corticostriatal reward circuit, observed across four independent cohorts (33, 39–41); this pattern is qualified by null nucleus-accumbens- and putamen-seed findings in one of these cohorts (39). Default-mode-network regions were involved in seven of nine cohorts but with markedly heterogeneous directions of change (28, 36, 40, 43), whereas anterior insula engagement that survived between-group correction was confined to acute stimulation-state and task paradigms (30), and stimulation-state connectivity effects were state- and frequency-band-specific (32). Explicitly null analyses were common throughout the literature, and the only machine-learning prediction model was evaluated by internal cross-validation within a single cohort (35).

Together, these observations support a strictly hypothesis-generating account: auricular vagus neuromodulation is accompanied by distributed changes in circuits implicated in mood, reward, and cognitive control, but the evidence establishes neither causal pathways nor clinically actionable imaging markers. No included study used randomized allocation (6, 36), the credibility of sham stimulation was limited by cutaneous tingling and somatosensory sensations (6), no study tested mediation, and findings from TECAS cannot be attributed to auricular vagal afferents alone (36). Reported brain–symptom correlations were largely uncorrected and exploratory, and candidate predictive signatures require independent external validation before any clinical use (6, 56).

Confirmatory progress will require randomized, sham-controlled, multi-center trials with adequate sample sizes and preregistered imaging endpoints; harmonized stimulation and acquisition parameters with standardized reporting (10); explicit reporting of null analyses; designs that separate acute stimulation-state from longitudinal treatment effects and include healthy controls scanned at both timepoints; formal mediation analyses; and independent external validation of candidate predictive signatures in adequately powered cohorts (6, 56). Until such evidence is available, neuroimaging findings in this field should guide mechanism-oriented research and trial design rather than clinical decision-making.

## Data Availability

The original contributions presented in the study are included in the article/**Supplementary Material**. Further inquiries can be directed to the corresponding author/s.
