# Supplementary material for: Large-scale brain network modulation by auricular vagus neuromodulation in major depressive disorder: a systematic review of neuroimaging evidence
Source: Front Psychiatry. 2026 Sep 30;17:1972200. doi: 10.3389/fpsyt.2026.1972200 (PMC13664290; doi:10.3389/fpsyt.2026.1972200)
Supplement: Supplementary file 1 [file DataSheet1.docx]

**Supplementary Methods**

Inter-rater agreement across review stages. Two reviewers independently performed screening, data extraction, and quality appraisal, with all disagreements resolved by discussion and consensus (unresolved screening decisions were referred to a third reviewer). Percentage agreement was 95.6% at title/abstract screening (842 records screened) and 93.2% at full-text assessment (36 full texts assessed), 92.1% across all data-extraction variables, and 89.3% across all JBI item-level quality judgments for the 17 included reports. These descriptive agreement statistics were moved from the main text to the Supplementary Methods in the revision to keep the Methods concise; the screening, extraction, and appraisal procedures themselves are unchanged.
