# Supplementary material for: Large-scale brain network modulation by auricular vagus neuromodulation in major depressive disorder: a systematic review of neuroimaging evidence
Source: Front Psychiatry. 2026 Sep 30;17:1972200. doi: 10.3389/fpsyt.2026.1972200 (PMC13664290; doi:10.3389/fpsyt.2026.1972200)
Supplement: Supplementary file 2 [file Table1.docx]

**Supplementary Table S1. Database-specific search strategies**

| **Database (hit count)** | **Strategy (verbatim)** |
| --- | --- |
| PubMed / MEDLINE | ("Depressive Disorder"[Mesh] OR "Depressive Disorder, Major"[Mesh] OR depression[tiab] OR depressive[tiab] OR MDD[tiab] OR TRD[tiab]) AND ("Vagus Nerve Stimulation"[Mesh] OR "Transcutaneous Electric Nerve Stimulation"[Mesh] OR "vagus nerve stimulation"[tiab] OR "vagus nerve"[tiab] OR taVNS[tiab] OR tVNS[tiab] OR "auricular vagus"[tiab] OR "transcutaneous auricular"[tiab] OR "transcutaneous vagal"[tiab] OR "auricular stimulation"[tiab] OR "auricular acupoint"[tiab] OR "auricular electroacupuncture"[tiab] OR "ear acupuncture"[tiab] OR "ear acupoint"[tiab] OR TECAS[tiab] OR "cranial-auricular"[tiab] OR "cranial auricular"[tiab]) AND ("Neuroimaging"[Mesh] OR "Magnetic Resonance Imaging"[Mesh] OR "Brain Mapping"[Mesh] OR "Diffusion Tensor Imaging"[Mesh] OR "Connectome"[Mesh] OR neuroimaging[tiab] OR neuroimag*[tiab] OR fMRI[tiab] OR "functional MRI"[tiab] OR "functional magnetic resonance"[tiab] OR "resting-state"[tiab] OR "resting state"[tiab] OR rs-fMRI[tiab] OR rsfMRI[tiab] OR ALFF[tiab] OR ReHo[tiab] OR "functional connectivity"[tiab] OR "regional homogeneity"[tiab] OR "independent component analysis"[tiab] OR "brain network"[tiab] OR connectom*[tiab] OR "graph theory"[tiab] OR "graph theoretical"[tiab] OR topological[tiab] OR "small-world"[tiab] OR "machine learning"[tiab] OR "structural MRI"[tiab] OR DTI[tiab] OR "diffusion tensor"[tiab] OR "fractional anisotropy"[tiab] OR EEG[tiab] OR electroencephalograph*[tiab] OR "brain imaging"[tiab] OR "neural activity"[tiab]) Date limit: ("1900/01/01"[pdat] : "2026/06/30"[pdat]). |
| Embase | #1 'major depression'/exp OR 'depression'/exp OR depression:ti,ab,kw OR depressive:ti,ab,kw OR 'major depressive disorder':ti,ab,kw OR MDD:ti,ab,kw OR 'treatment resistant depression':ti,ab,kw OR TRD:ti,ab,kw #2 'vagus nerve stimulation'/exp OR 'transcutaneous vagus nerve stimulation'/exp OR 'transcutaneous electrical nerve stimulation'/exp OR 'transcutaneous auricular vagus nerve stimulation':ti,ab,kw OR taVNS:ti,ab,kw OR tVNS:ti,ab,kw OR 'transcutaneous vagus nerve stimulation':ti,ab,kw OR 'auricular vagus nerve stimulation':ti,ab,kw OR 'vagus nerve stimulation':ti,ab,kw OR 'vagus nerve':ti,ab,kw OR 'auricular vagus':ti,ab,kw OR 'transcutaneous auricular':ti,ab,kw OR 'transcutaneous vagal':ti,ab,kw OR 'auricular stimulation':ti,ab,kw OR 'auricular acupoint':ti,ab,kw OR 'auricular electroacupuncture':ti,ab,kw OR 'ear acupuncture':ti,ab,kw OR 'ear acupoint':ti,ab,kw OR TECAS:ti,ab,kw OR 'cranial-auricular':ti,ab,kw OR 'cranial auricular':ti,ab,kw #3 'functional magnetic resonance imaging'/exp OR 'neuroimaging'/exp OR 'brain mapping'/exp OR 'diffusion tensor imaging'/exp OR 'connectome'/exp OR 'electroencephalography'/exp OR neuroimaging:ti,ab,kw OR neuroimag*:ti,ab,kw OR fMRI:ti,ab,kw OR 'functional MRI':ti,ab,kw OR 'functional magnetic resonance':ti,ab,kw OR 'resting-state':ti,ab,kw OR 'resting state':ti,ab,kw OR 'rs-fMRI':ti,ab,kw OR rsfMRI:ti,ab,kw OR ALFF:ti,ab,kw OR ReHo:ti,ab,kw OR 'functional connectivity':ti,ab,kw OR 'regional homogeneity':ti,ab,kw OR 'independent component analysis':ti,ab,kw OR 'brain network':ti,ab,kw OR connectom*:ti,ab,kw OR 'graph theory':ti,ab,kw OR 'graph theoretical':ti,ab,kw OR topological:ti,ab,kw OR 'small-world':ti,ab,kw OR 'machine learning':ti,ab,kw OR 'structural MRI':ti,ab,kw OR DTI:ti,ab,kw OR 'diffusion tensor':ti,ab,kw OR 'fractional anisotropy':ti,ab,kw OR EEG:ti,ab,kw OR electroencephalograph*:ti,ab,kw OR 'brain imaging':ti,ab,kw OR 'neural activity':ti,ab,kw #4 #1 AND #2 AND #3 Date limit: ≤ 30 Jun 2026. |
| Cochrane CENTRAL | #1 MeSH descriptor: [Depressive Disorder, Major] explode all trees; MeSH descriptor: [Depressive Disorder] explode all trees; (depression or depressive or "major depressive disorder" or MDD or "treatment-resistant depression" or TRD):ti,ab,kw #2 MeSH descriptor: [Vagus Nerve Stimulation] explode all trees; MeSH descriptor: [Transcutaneous Electric Nerve Stimulation] explode all trees; ("transcutaneous auricular vagus nerve stimulation" or taVNS or tVNS or "transcutaneous vagus nerve stimulation" or "auricular vagus nerve stimulation" or "vagus nerve stimulation" or "vagus nerve" or "auricular vagus" or "transcutaneous auricular" or "transcutaneous vagal" or "auricular stimulation" or "auricular acupoint" or "auricular electroacupuncture" or "ear acupuncture" or "ear acupoint" or TECAS or "cranial-auricular" or "cranial auricular"):ti,ab,kw #3 MeSH descriptor: [Neuroimaging] explode all trees; MeSH descriptor: [Magnetic Resonance Imaging] explode all trees; MeSH descriptor: [Brain Mapping] explode all trees; MeSH descriptor: [Diffusion Tensor Imaging] explode all trees; MeSH descriptor: [Connectome] explode all trees; (neuroimaging or neuroimag* or fMRI or "functional MRI" or "functional magnetic resonance" or "resting-state" or "resting state" or "rs-fMRI" or rsfMRI or ALFF or ReHo or "functional connectivity" or "regional homogeneity" or "independent component analysis" or "brain network" or connectom* or "graph theory" or "graph theoretical" or topological or "small-world" or "machine learning" or "structural MRI" or DTI or "diffusion tensor" or "fractional anisotropy" or EEG or electroencephalograph* or "brain imaging" or "neural activity"):ti,ab,kw #4 #1 AND #2 AND #3 |
| Web of Science Core Collection | TS=(depression OR depressive OR "major depressive disorder" OR MDD OR "treatment-resistant depression" OR TRD) AND TS=("transcutaneous auricular vagus nerve stimulation" OR taVNS OR tVNS OR "transcutaneous vagus nerve stimulation" OR "auricular vagus nerve stimulation" OR "vagus nerve stimulation" OR "vagus nerve" OR "auricular vagus" OR "transcutaneous auricular" OR "transcutaneous vagal" OR "auricular stimulation" OR "auricular acupoint" OR "auricular electroacupuncture" OR "ear acupuncture" OR "ear acupoint" OR TECAS OR "cranial-auricular" OR "cranial auricular") AND TS=(neuroimaging OR neuroimag* OR fMRI OR "functional MRI" OR "functional magnetic resonance" OR "resting-state" OR "resting state" OR "rs-fMRI" OR rsfMRI OR ALFF OR ReHo OR "functional connectivity" OR "regional homogeneity" OR "independent component analysis" OR "brain network*" OR connectom* OR "graph theory" OR "graph theoretical" OR topological OR "small-world" OR "machine learning" OR "structural MRI" OR DTI OR "diffusion tensor" OR "fractional anisotropy" OR EEG OR electroencephalograph* OR "brain imaging" OR "neural activity") Year ≤ 2026. TS = topic (title/abstract/author keywords/Keywords Plus). |
| PsycINFO | 1 exp Major Depression/ 2 exp Depression/ 3 (depression or depressive or "major depressive disorder" or MDD or "treatment-resistant depression" or TRD).ti,ab,hw. 4 1 or 2 or 3 5 exp Vagus Nerve Stimulation/ 6 exp Transcutaneous Nerve Stimulation/ 7 ("transcutaneous auricular vagus nerve stimulation" or taVNS or tVNS or "transcutaneous vagus nerve stimulation" or "auricular vagus nerve stimulation" or "vagus nerve stimulation" or "vagus nerve" or "auricular vagus" or "transcutaneous auricular" or "transcutaneous vagal" or "auricular stimulation" or "auricular acupoint" or "auricular electroacupuncture" or "ear acupuncture" or "ear acupoint" or TECAS or "cranial-auricular" or "cranial auricular").ti,ab,hw. 8 5 or 6 or 7 9 exp Neuroimaging/ 10 exp Magnetic Resonance Imaging/ 11 exp Brain Mapping/ 12 (neuroimaging or neuroimag* or fMRI or "functional MRI" or "functional magnetic resonance" or "resting-state" or "resting state" or "rs-fMRI" or rsfMRI or ALFF or ReHo or "functional connectivity" or "regional homogeneity" or "independent component analysis" or "brain network*" or connectom* or "graph theory" or "graph theoretical" or topological or "small-world" or "machine learning" or "structural MRI" or "diffusion tensor" or "fractional anisotropy" or EEG or electroencephalograph* or "brain imaging" or "neural activity").ti,ab,hw. 13 9 or 10 or 11 or 12 14 4 and 8 and 13  limit 14 to yr="1806-2026" |

Three conceptual blocks: (1) major depression; (2) auricular vagus neuromodulation (taVNS / TECAS); (3) neuroimaging (MRI/fMRI/FC/ICA/graph theory/ML/DTI/EEG). No language or publication-type restrictions at the search stage; English-language and study-design exclusions applied at screening and documented in Supplementary Table S2.
