# Supplementary material for: Large-scale brain network modulation by auricular vagus neuromodulation in major depressive disorder: a systematic review of neuroimaging evidence
Source: Front Psychiatry. 2026 Sep 30;17:1972200. doi: 10.3389/fpsyt.2026.1972200 (PMC13664290; doi:10.3389/fpsyt.2026.1972200)
Supplement: Supplementary file 3 [file Table2.docx]

Supplementary Table S2. Full-text reports excluded after eligibility assessment, with reasons (n = 22).

| **#** | **Excluded report** | **Reason for exclusion** |
| --- | --- | --- |
| 1 | Zhang H, et al. (2026) Behav Brain Res[1] | **(P) Population.** Healthy participants only — immediate multi-band resting-state fMRI effects of taVNS were examined in healthy males; no participants with MDD were enrolled. |
| 2 | Zhang Y, et al. (2024) IEEE J Biomed Health Inform[2] | **(P) Population.** Healthy participants only — insula–medial prefrontal connectivity modulation by taVNS was tested in healthy volunteers; no participants with MDD. |
| 3 | Lloyd B, et al. (2023) Brain Stimul[3] | (**O) Outcome.** No MRI neuroimaging endpoint — taVNS effects were assessed using pupillometry and EEG alpha power only in healthy volunteers, with no participants with MDD; studies without a magnetic-resonance neuroimaging outcome were not eligible. |
| 4 | von Wrede R, et al. (2022) Brain Sci[4] | (**O) Outcome.** No MRI neuroimaging endpoint — taVNS-induced network changes were derived from continuous EEG recordings only (eighteen subjects, morning vs. afternoon sessions); studies with EEG as the sole neuroimaging modality were excluded by the pre-specified criteria. |
| 5 | Mao Y, et al. (2022) Front Hum Neurosci[5] | **(P) Population.** Healthy participants only — sub-threshold taVNS effects on cingulate and insula resting-state connectivity were examined in healthy volunteers; no participants with MDD. |
| 6 | Yakunina N, et al. (2017) Neuromodulation[6] | **(P) Population.** Healthy participants only — fMRI-based optimization of transcutaneous VNS parameters was conducted in healthy volunteers; no participants with MDD. |
| 7 | Dietrich S, et al. (2008) Biomed Tech (Berl)[7] | **(P) Population.** Healthy participants only — brainstem and cerebral fMRI activation during transcutaneous VNS was measured in healthy volunteers; no participants with MDD. |
| 8 | Kraus T, et al. (2007) J Neural Transm (Vienna)[8] | **(P) Population.** Healthy participants only — BOLD deactivation and mood effects of transcutaneous VNS were examined in healthy volunteers; no participants with MDD. |
| 9 | Obst MA, et al. (2022) Brain Imaging Behav[9] | **(P) Population.** Primary diagnosis not MDD — five weeks of intermittent tVNS with fMRI/machine-learning network analysis was conducted in participants with overweight/obesity (a metabolic cohort). |
| 10 | Yakunina N, et al. (2018) PLoS One[10] | **(P) Population.** Primary diagnosis not MDD — BOLD fMRI effects of transcutaneous VNS were studied in patients with chronic tinnitus. |
| 11 | Feng B, et al. (2026) Neurogastroenterol Motil[11] | **(P) Population.** Animal study — antidepressant-like effects of taVNS on brainstem and entorhinal cortex activity were examined in a rodent model; animal studies were excluded by the pre-specified criteria. |
| 12 | Wang X, et al. (2024) Heliyon[12] | **(I) Intervention.** Not auricular vagus neuromodulation — electro-acupuncture was delivered at scalp and hand body acupoints (Baihui DU20, Sishencong EX-HN1, Taiyang EX-HN5, Yintang EX-HN3, Hegu LI4) in first-episode MDD, with no auricular stimulation; non-auricular body electro-acupuncture was not eligible. |
| 13 | Zhu L, et al. (2026) IEEE Trans Neural Syst Rehabil Eng[13] | **(O) Outcome.** No MRI neuroimaging endpoint — TECAS effects in mild-to-moderate depression were assessed using EEG functional networks only; studies with EEG as the sole neuroimaging modality were excluded by the pre-specified criteria. |
| 14 | Sun J, et al. (2023) Psychiatry Res Neuroimaging[14] | **(O) Outcome.** No treatment-related imaging endpoint — a cross-sectional case–control comparison of striatal resting-state connectivity between treatment-resistant and non-treatment-resistant depression, with no taVNS/TECAS intervention and no pre–post treatment imaging. |
| 15 | Rong P, et al. (2016) J Affect Disord[15] | **(O) Outcome.** Parent clinical report without neuroimaging outcomes — the nonrandomized controlled taVNS trial in MDD (parent trial ChiCTR-TRC-11001201, cohort C1) reports clinical outcomes only; its imaging findings are represented by the included linked reports [26–30]. |
| 16 | Borgmann D, et al. (2021) NeuroImage[16] | **(S) Study design.** Technical note (methods demonstration) of fMRI brainstem responses to tVNS, rather than an original clinical neuroimaging study; enrolled participants were healthy volunteers. |
| 17 | Kong J, et al. (2018) Front Psychiatry[17] | **(S) Study design.** Narrative review ("state of the art and future perspectives") rather than an original study; reviews and meta-analyses were excluded by the pre-specified criteria. |
| 18 | Li XJ, et al. (2019) Brain Stimul[18] | **(S) Study design.** Single case report of taVNS in treatment-resistant depression monitored by resting-state fMRI and MRS; case reports were excluded by the pre-specified criteria. |
| 19 | Yang S, et al. (2022) Front Psychiatry[19] | **(S) Study design.** Trial protocol without results — study-protocol paper for the TECAS versus escitalopram RCT (parent trial ChiCTR2000029109, cohort C6), with no neuroimaging findings; imaging results from this trial are represented by the included linked reports [34–36]. |
| 20 | Ma Y, et al. (2023) Zhongguo Zhen Jiu[20] | **(L) Language.** Full text published in Chinese; eligibility was restricted to English-language reports. |
| 21 | Chen LM, et al. (2021) Zhen Ci Yan Jiu[21] | **(L) Language.** Full text published in Chinese; eligibility was restricted to English-language reports. |
| 22 | Sun J, et al. (2022) Front Psychiatry[22] | **Linked report.** Companion analysis from the same parent trial (ChiCTR-1800014277, cohort C2), sharing participants and the same immediate post-stimulation ALFF endpoint with the included report Sun et al., 2023 [32]; it contributed no additional imaging metric or stimulation timepoint, and participants were counted once at the cohort level per the pre-specified linked-report rule. |

Reference

[1] H. Zhang, X. Xue, Z. Mu, C. Wang, J. Zheng, Z. Wang, Y. Han, S. Gao, W. Zhang, A. Ren, L. Chen, X. Jia, and X. Wang, Immediate modulatory effects of transcutaneous auricular vagus nerve stimulation in healthy males: A multi-bands resting-state fMRI study. Behav Brain Res 498 (2026) 115922.

[2] Y. Zhang, P. Lin, R. Wang, J. Zhou, X. Xu, W. Jiang, X. Pu, and S. Ge, Insula-Medial Prefrontal Cortex Functional Connectivity Modulated by Transcutaneous Auricular Vagus Nerve Stimulation: An fMRI Study. IEEE J Biomed Health Inform 28 (2024) 5962-5970.

[3] B. Lloyd, F. Wurm, R. de Kleijn, and S. Nieuwenhuis, Short-term transcutaneous vagus nerve stimulation increases pupil size but does not affect EEG alpha power: A replication of Sharon et al. (2021, Journal of Neuroscience). Brain Stimul 16 (2023) 1001-1008.

[4] R. von Wrede, T. Brohl, T. Rings, J. Pukropski, C. Helmstaedter, and K. Lehnertz, Modifications of Functional Human Brain Networks by Transcutaneous Auricular Vagus Nerve Stimulation: Impact of Time of Day. Brain sciences 12 (2022).

[5] Y. Mao, C. Chen, M. Falahpour, K.H. MacNiven, G. Heit, V. Sharma, K. Alataris, and T.T. Liu, Effects of Sub-threshold Transcutaneous Auricular Vagus Nerve Stimulation on Cingulate Cortex and Insula Resting-state Functional Connectivity. Front Hum Neurosci 16 (2022) 862443.

[6] N. Yakunina, S.S. Kim, and E.C. Nam, Optimization of Transcutaneous Vagus Nerve Stimulation Using Functional MRI. Neuromodulation 20 (2017) 290-300.

[7] S. Dietrich, J. Smith, C. Scherzinger, K. Hofmann-Preiss, T. Freitag, A. Eisenkolb, and R. Ringler, A novel transcutaneous vagus nerve stimulation leads to brainstem and cerebral activations measured by functional MRI. Biomedizinische Technik. Biomedical engineering 53 (2008) 104-11.

[8] T. Kraus, K. Hosl, O. Kiess, A. Schanze, J. Kornhuber, and C. Forster, BOLD fMRI deactivation of limbic and temporal brain structures and mood enhancing effect by transcutaneous vagus nerve stimulation. Journal of neural transmission (Vienna, Austria : 1996) 114 (2007) 1485-93.

[9] M.A. Obst, A. Al-Zubaidi, M. Heldmann, J.M. Nolde, N. Blumel, S. Kannenberg, and T.F. Munte, Five weeks of intermittent transcutaneous vagus nerve stimulation shape neural networks: a machine learning approach. Brain Imaging Behav 16 (2022) 1217-1233.

[10] N. Yakunina, S.S. Kim, and E.C. Nam, BOLD fMRI effects of transcutaneous vagus nerve stimulation in patients with chronic tinnitus. PLoS One 13 (2018) e0207281.

[11] B. Feng, S. Li, N. Zou, J. Han, Y. Wang, J.D.Z. Chen, J. Zhang, W. Wei, and P. Rong, Transcutaneous Auricular Vagus Nerve Stimulation Mediates Antidepressant Effects by Altering Right Brainstem and Right Entorhinal Cortex Activity in a Rodent Model Functional Dyspepsia. Neurogastroenterol Motil 38 (2026) e70234.

[12] X. Wang, P. Luo, L. Zhang, J. Sun, J. Cao, Z. Lei, H. Yang, X. Lv, J. Liu, X. Yao, S. Li, and J. Fang, Altered functional brain activity in first-episode major depressive disorder treated with electro-acupuncture: A resting-state functional magnetic resonance imaging study. Heliyon 10 (2024) e29613.

[13] L. Zhu, Y. Zhao, C. Zheng, X. Xiao, Y. Wang, J. Liu, P. Rong, and B. Hu, Treating Mild to Moderate Depression With Transcutaneous Electrical Cranial-Auricular Vagus Nerve Stimulation: A Study of Brain Functional Networks. IEEE Trans Neural Syst Rehabil Eng 34 (2026) 455-467.

[14] J. Sun, L. Xu, Y. Ma, C. Guo, Z. Du, S. Gao, Y. Luo, Q. Chen, Y. Hong, X. Yu, X. Xiao, and J. Fang, Different characteristics of striatal resting-state functional conectivity in treatment-resistant and non-treatment-resistant depression. Psychiatry Res Neuroimaging 328 (2023) 111567.

[15] P. Rong, J. Liu, L. Wang, R. Liu, J. Fang, J. Zhao, Y. Zhao, H. Wang, M. Vangel, S. Sun, H. Ben, J. Park, S. Li, H. Meng, B. Zhu, and J. Kong, Effect of transcutaneous auricular vagus nerve stimulation on major depressive disorder: A nonrandomized controlled pilot study. J Affect Disord 195 (2016) 172-9.

[16] D. Borgmann, L. Rigoux, B. Kuzmanovic, S. Edwin Thanarajah, T.F. Munte, H. Fenselau, and M. Tittgemeyer, Technical Note: Modulation of fMRI brainstem responses by transcutaneous vagus nerve stimulation. Neuroimage 244 (2021) 118566.

[17] J. Kong, J. Fang, J. Park, S. Li, and P. Rong, Treating Depression with Transcutaneous Auricular Vagus Nerve Stimulation: State of the Art and Future Perspectives. Front Psychiatry 9 (2018) 20.

[18] X.J. Li, L. Wang, H.X. Wang, L. Zhang, G.L. Zhang, P.J. Rong, and J.L. Fang, The effect of transcutaneous auricular vagus nerve stimulation on treatment-resistant depression monitored by resting-state fMRI and MRS: The first case report. Brain Stimul 12 (2019) 377-379.

[19] S. Yang, Z. Qin, X. Yang, M.Y. Chan, S. Zhang, P. Rong, X. Hou, G. Jin, F. Xu, Y. Liu, and Z.J. Zhang, Transcutaneous Electrical Cranial-Auricular Acupoint Stimulation vs. Escitalopram for Patients With Mild-to-Moderate Depression (TECAS): Study Design for a Randomized Controlled, Non-inferiority Trial. Front Psychiatry 13 (2022) 829932.

[20] Y. Ma, C.L. Guo, J.F. Sun, S.S. Gao, Y. Luo, Q.Y. Chen, Y. Hong, L. Zhang, J.D. Cao, X. Xiao, P.J. Rong, and J.L. Fang, [Effect of transcutaneous auricular vagus nerve stimulation on functional connectivity in the related brain regions of patients with depression based on the resting-state fMRI]. Zhongguo Zhen Jiu 43 (2023) 367-73.

[21] L.M. Chen, X.J. Li, K. Xu, C.L. Guo, G.L. Zhang, M. Han, Z. Wang, P.J. Rong, J.K. He, J.F. Sun, F.Q. Xu, H.X. Wang, and J.L. Fang, [Functional MRI-based study on neuromechanism of trans-auricular vagus nerve stimulation for treatment-resistant depression]. Zhen ci yan jiu = Acupuncture research 46 (2021) 869-74.

[22] J. Sun, Y. Ma, Z. Du, Z. Wang, C. Guo, Y. Luo, L. Chen, D. Gao, X. Li, K. Xu, Y. Hong, F. Xu, X. Yu, X. Xiao, J. Fang, and X. Hou, Immediate Modulation of Transcutaneous Auricular Vagus Nerve Stimulation in Patients With Treatment-Resistant Depression: A Resting-State Functional Magnetic Resonance Imaging Study. Front Psychiatry 13 (2022) 923783.
