# Supplementary material for: Large-scale brain network modulation by auricular vagus neuromodulation in major depressive disorder: a systematic review of neuroimaging evidence
Source: Front Psychiatry. 2026 Sep 30;17:1972200. doi: 10.3389/fpsyt.2026.1972200 (PMC13664290; doi:10.3389/fpsyt.2026.1972200)
Supplement: Supplementary file 4 [file Table3.docx]

**bSupplementary Table S3. Effect-level coding matrix (126 rows; 27 explicitly null rows).**

| **Report (tag)** | **Outcome metric** | **Network (author-reported)** | **Network [inferred]** | **Direction (vs reference)** | **Comparator** | **Analysis scope** | **Multiplicity correction** | **Statistic** | **Source** |
| --- | --- | --- | --- | --- | --- | --- | --- | --- | --- |
| Fang 2016 F16-1 | ICA-DMN FC | DMN (ICA) | anterior insula[SN], fusiform[visual], thalamus[subcort] | ↓ real>sham (group×time) | group×time interaction | ICA network + WB dual regression | Z>2.6 + cluster p<.05 (>20 voxels, 1.5T) | NR | Fang 2016 Results+Fig2 |
| Fang 2016 F16-2 | ICA-DMN FC | DMN | precuneus/OPFC/TPJ[DMN] | ↑ real>sham (group×time) | group×time | ICA + WB | same as F16-1 | NR | Fang 2016 Fig3 |
| Fang 2016 F16-3 | ICA-DMN FC (opposite direction) | DMN | operculum/insula[SN], occipital[visual], mPFC/IPL/STG[DMN] | ↓ sham>real | group×time (opposite) | ICA + WB | same as F16-1 | NR | Fang 2016 Results |
| Fang 2016 F16-4 | DMN-FC change vs HAMD | DMN | DLPFC[FPN], rest[DMN/SN/visual] | negative correlation (ΔFC↑ = HAMD↓) | correlation (ΔFC vs ΔHAMD) | cluster-level correlation | uncorrected | NR | Fang 2016 Results |
| Fang 2016 F16-5 | DMN-FC change vs HAMD | DMN | DLPFC[FPN], rest[DMN/SN/visual] | positive correlation (ΔFC↑ = HAMD↑) | correlation | cluster-level correlation | uncorrected | NR | Fang 2016 Results |
| Liu 2016 L16-1 | R amygdala seed FC | limbic-emotion regulation (amygdala seed) | DLPFC[FPN/CEN] | ↑ within-real (post>baseline, p=.031) | within-group pre/post (real) | seed-based WB | voxel p<.005 + cluster FWE (>50 voxels) | z=4.04, cluster p=.031 | Liu 2016 Results+Fig |
| Liu 2016 L16-2 | R amygdala seed FC | limbic | DLPFC[FPN] | ↓ within-sham (post<baseline, p=.012) | within-group pre/post (sham) | seed-based WB | same as L16-1 | z=3.85, p=.012 | Liu 2016 Results |
| Liu 2016 L16-3 | R amygdala seed FC | limbic | DLPFC[FPN] | ↑ real>sham (group comparison) | group×time between | seed-based WB | same as L16-1 | z=3.72, p=.011 | Liu 2016 Fig2 |
| Liu 2016 L16-4 | L amygdala seed FC | limbic | — | null (no significant cluster) | between-group | seed-based WB | same as L16-1 | — | Liu 2016 Results |
| Liu 2016 L16-5 | ΔFC vs HAMD | limbic | [FPN] | negative correlation (FC↑ = HAMD↓) | correlation (adjusted age/sex) | cluster-level correlation | uncorrected | r=−0.42,p=.02; r=−0.55,p=.001; r=−0.46,p=.024 | Liu 2016 Results |
| Fang 2017 F17-1 | Task-block BOLD activation | authors: vagal afferent→brainstem/insula/cerebellum | insula/operculum[SN], cerebellar Crus1[cerebellar] | ↑ on-block activation (real, one-sample) | on vs implicit baseline (real) | WB GLM (block) | one-sample voxel p<.001 + cluster FWE | T9.11/T7.97/T6.40 | Fang 2017 Results |
| Fang 2017 F17-2 | Task-block BOLD deactivation | — | mPFC/OFC[DMN] | ↓ on-block deactivation (real) | on vs baseline (real) | WB GLM | same as F17-1 | T6.33, p=.008 | Fang 2017 Results |
| Fang 2017 F17-3 | Sham block activation | — | mid/inf frontal[FPN/ventral attention] | ↑ on-block (sham only) | on vs baseline (sham) | WB GLM | same as F17-1 | T7.98; T5.25,p=.023 | Fang 2017 Results |
| Fang 2017 F17-4 | Sham block deactivation | — | PCC/precuneus/hippocampus/mPFC[DMN]; calcarine[visual] | ↓ on-block (sham only; sham-specific, not a taVNS effect) | on vs baseline (sham) | WB GLM | same as F17-1 | T7.16/T6.12/T5.03 | Fang 2017 Results |
| Fang 2017 F17-5 | Between-group activation (ROI-SVC) | authors: insula as cortical target of vagal afferents | anterior insula[SN] | ↑ real>sham (FWE-SVC p<.05) | real vs sham (between) | WB + ROI SVC | group FWE-SVC p<.05 | FWE-SVC p<.05 | Fang 2017 Fig4 |
| Fang 2017 F17-6 | Between-group (opposite direction) | — | — | null (sham>real direction ns) | real vs sham (opposite) | WB GLM | FWE | — | Fang 2017 Results |
| Fang 2017 F17-7 | Brainstem/NTS activation | — | — | null (authors explicitly report no NTS activation observed) | on vs baseline (NTS negative) | WB GLM | FWE | — | Fang 2017 Results/Discussion |
| Fang 2017 F17-8 | L anterior insula activation vs 4-wk HAMD | insula target | [SN] | negative correlation (activation↑ = HAMD↓); sham null | correlation (vs 4-wk HAMD) | cluster-level correlation | uncorrected | real r(17)=−0.65,p=.01; sham p=.6 (null) | Fang 2017 Results |
| Tu 2018 T18-1 | MH seed FC | hypothalamic–limbic/DMN regulatory framework | rACC[limbic/SN], MFG[FPN] | ↓ real<sham (stimulation state) | stimulation-state real vs sham | seed-based WB | voxel p<.001 + cluster FDR p<.05 | T4.27,p=.023; T4.73,p=.002 | Tu 2018 Fig2 |
| Tu 2018 T18-2 | MH seed FC | — | cerebellar[cerebellar] | ↑ real>sham (stimulation state) | stimulation-state real vs sham | seed-based WB | same as T18-1 | T4.09,p=.020; T3.95,p=.035 | Tu 2018 Results |
| Tu 2018 T18-3 | MH–rACC FC | [limbic/SN] | — | ↓ real paired p=.004; sham p=.18 (null) | on-vs-off paired | seed-based WB | same as T18-1 | paired p=.004 vs p=.18 | Tu 2018 Results |
| Tu 2018 T18-4 | Stimulation-state MH–rACC vs HAMD | [limbic/SN] | — | positive correlation (real r=0.58,p=.02); sham r=0.37,p=.15 (null) | correlation (stimulation state) | cluster-level correlation | uncorrected | r=0.58,p=.02 | Tu 2018 Results |
| Tu 2018 T18-5 | Resting-state MH–rACC vs HAMD | — | — | null (resting-state: no correlation with HAMD improvement; stimulation-state only) | correlation (resting-state) | cluster-level correlation | — | — | Tu 2018 Results |
| Tu 2018 T18-6 | MH–MFG/cerebellum modulation vs HAMD | — | — | null (connection modulated but no correlation with HAMD) | correlation (negative) | cluster-level correlation | — | — | Tu 2018 Results |
| Tu 2018 T18-7 | LH seed FC | — | MCC[SN], putamen[striatal/reward] | ↓ real<sham (stimulation state) | stimulation-state real vs sham | seed-based WB | voxel p<.001 + cluster FDR | NR | Tu 2018 Fig3 |
| Tu 2018 T18-8 | LH–MCC/putamen vs HAMD | — | — | null (no correlation) | correlation | cluster-level correlation | — | — | Tu 2018 Results |
| Tu 2018 T18-9 | 4 preset non-hypothalamic control seeds | — | — | null (all 4 control seeds: no significant cluster) | stimulation-state real vs sham (null controls) | seed-based WB | cluster FDR | — | Tu 2018 Results |
| Wang 2018 W18-1 | NAc seed FC (slow-5) | reward circuit (NAc seed, authors) | MPFC/rACC[DMN/limbic] | ↑ real on vs off (slow-5) | on-vs-off paired (real) | seed-based WB | voxel p<.005 + cluster FWE (>50 voxels) | z=4.07 | Wang 2018 Table |
| Wang 2018 W18-2 | NAc seed FC (slow-4/typical) | — | — | null (real slow-4/typical on vs off ns) | on-vs-off paired (real null) | seed-based WB | same as W18-1 | — | Wang 2018 Results |
| Wang 2018 W18-3 | NAc seed FC sham group (3 bands) | — | — | null (sham on vs off all 3 bands ns) | on-vs-off paired (sham null) | seed-based WB | same as W18-1 | — | Wang 2018 Results |
| Wang 2018 W18-4 | NAc seed FC (slow-5) between-group | reward circuit | [DMN/limbic] | ↑ real>sham | stimulation-state real vs sham | seed-based WB | same as W18-1 | Z=5.07 | Wang 2018 Table3 |
| Wang 2018 W18-5 | NAc seed FC (slow-4) between-group | — | — | null (slow-4 between-group ns) | stimulation-state real vs sham (null) | seed-based WB | same as W18-1 | — | Wang 2018 Results |
| Wang 2018 W18-6 | NAc seed FC (typical) right seed between-group | reward circuit | occipital/fusiform[visual], insula[SN] | ↑ typical between-group real>sham | stimulation-state real vs sham | seed-based WB | same as W18-1 | Z5.15/Z3.80/Z4.37. Coordinates NV (typical-band insular cluster; flagged in main text) | Wang 2018 Table3 |
| Wang 2018 W18-7 | NAc seed FC (opposite direction) | — | — | null (real<sham direction ns) | stimulation-state real vs sham (opposite null) | seed-based WB | same as W18-1 | — | Wang 2018 Results |
| Wang 2018 W18-8 | ΔNAc–PFC FC vs HAMD | reward circuit | OFC/MPFC/sgACC[DMN/reward], inf frontal[FPN] | negative correlation (FC↑ = HAMD↓) | correlation (real) | cluster-level correlation | uncorrected | z4.30/k2759; z4.56/k256 | Wang 2018 Results |
| Wang 2018 W18-9 | Same correlation (sham) | — | — | null (sham group, no correlation) | correlation (sham null) | cluster-level correlation | — | — | Wang 2018 Results |
| Ma 2022 M22-1 | ReHo | reward (mOFC, authors) | [reward/OFC] | interaction: TRD baseline>HC; post-TRD ↓ (normalized); HC ns | group×time + post-hoc | WB voxelwise | GRF voxel P<.01 + cluster P<.05 (two-sided); post-hoc Bonferroni P<.0125; covariates sex/age/education/FD | F=18.06, P<.0001 | Ma 2022 Table3+Fig |
| Ma 2022 M22-2 | ReHo (time main effect) | reward (mOFC); cerebellar/temporal/precuneus[DMN] | cerebellar[cerebellar], ITG[ventral attention], precuneus[DMN] | ↓ 4 clusters ↑ 1 cluster (post vs pre, time effect) | time main effect | WB voxelwise | same GRF | NR | Ma 2022 Table4 |
| Ma 2022 M22-3 | ReHo (group main effect) | — | — | null (ReHo group main effect: no significant cluster) | group main effect (null) | WB voxelwise | same as M22-1 | — | Ma 2022 Results |
| Ma 2022 M22-4 | Right mOFC seed FC | FPCN (authors) | [FPN/DMN boundary] | interaction: TRD baseline low, post ↑ | group×time | seed-based WB | same as M22-1 | F=11.66,P=.001 | Ma 2022 Table5 |
| Ma 2022 M22-5 | Right mOFC seed FC | FPCN | [FPN/somatosensory] | interaction significant but MDD post-↑ not reaching corrected threshold (null: t=1.623,P=.113); HC ↓ (t=8.704,P<.001) | group×time (post-hoc within null) | seed-based WB | same as M22-1 | F=16.75,P<.0001 (interaction); MDD post-hoc ns | Ma 2022 Table5 |
| Ma 2022 M22-6 | mOFC seed FC (time main effect) | — | MCC[SN], IPL[DMN/FPN], MFG[FPN] | ↓ (post vs pre, time effect) | time main effect | seed-based WB | same as M22-1 | NR | Ma 2022 Table7 |
| Ma 2022 M22-7 | mOFC seed FC (group main effect) | — | — | null (FC group main effect: no significant cluster) | group main effect (null) | seed-based WB | same as M22-1 | — | Ma 2022 Results |
| Ma 2022 M22-8 | ReHo/FC change vs clinical | — | — | null (HAMD/HAMA change uncorrelated with ReHo, mOFC-FC change; Fig5) | correlation (null) | cluster-level correlation | — | — | Ma 2022 Fig5 |
| Sun 2023 S23-1 | ALFF (baseline cross-sectional) | DMN (precuneus, authors) | [DMN] | ↓ MDD<HC (T=−2.881) | between-group (cross-sectional) | WB voxelwise | GRF voxel P<.005 + cluster P<.05; <60 voxels discarded; covariates per team convention | T=−2.881 | Sun 2023 Table1 |
| Sun 2023 S23-2 | ALFF (baseline cross-sectional) | DMN (angular gyrus) | [DMN/FPN boundary] | ↑ MDD>HC (T=5.105) | between-group (cross-sectional) | WB voxelwise | same as S23-1 | T=5.105 | Sun 2023 Table1 |
| Sun 2023 S23-3 | ALFF (pre/post; right precuneus) | DMN | [DMN] | ↓ (abstract/results text: "ALFF in the right precuneus was decreased"; however Table2 prints T=+4.137 — sign inconsistency between abstract/Discussion and Table; per abstract + team confirmation set as ↓; flagged) | within-group pre/post | WB voxelwise | same as S23-1 | T=4.137 (sign flagged) | Sun 2023 abstract + Table2 |
| Sun 2023 S23-4 | Precuneus seed FC (pre/post) | DMN (PCC/angular) + CCN (MFG/dlPFC, authors' dual-network interpretation) | [DMN internal + FPN] | ↓ (3 clusters FC all decreased post-treatment) | within-group pre/post | seed-based WB | same as S23-1 | T=−4.935/−4.029/−4.967 | Sun 2023 Table2 |
| Sun 2023 S23-5 | Baseline FC vs baseline HAMD-17 | DMN | [DMN] | negative correlation (FC↑ = HAMD↓) | correlation (baseline) | cluster-level correlation | uncorrected | r=−0.288,P=.032 | Sun 2023 Results |
| Sun 2024 S24-1 | ML prediction (baseline FC → outcome) | authors: CSPT cortico-striato-pallido-thalamic + hippocampus(DMN) + cerebellum | — | predictive (HAMD-drop regression model) | ML prediction (regression) | ML feature-level | feature selection P<.01; permutation 5,000; internal nested LOOCV | R=0.66, MAE=0.15, R²=0.44 (32-feature model R=0.88) | Sun 2024 Results+Table2/3 |
| Sun 2024 S24-2 | ML responder classification | CSPT/hippocampus/cerebellum | — | predictive (responder vs non-responder classification) | ML prediction (classification) | ML feature-level | nested LOOCV + 5,000 permutation | AUC=0.856; accuracy=76.6%; sensitivity=81.0%; specificity=71.4% | Sun 2024 Results |
| Sun 2024 S24-3 | 11 key FC features (Table3) | CSPT (thalamus/putamen/globus pallidus), hippocampus/parahippocampus[DMN], cerebellar network; angular[DMN], inf frontal三角[FPN] | — | mostly negative correlations (baseline striatal-hippocampal/cerebellar connectivity↑ = improvement↓); 2 vermis-hippocampal ns (null features) | correlation feature weights | ML feature-level | P<.01 feature selection | NR | Sun 2024 Table3 |
| Chen 2022 C22-1 | Bilateral NAc seed FC | striatal/reward | — | null (P=.07) | within-group pre/post (null) | seed-based WB | GRF voxel p<.01 + cluster p<.05; <15 voxels discarded | P=.07 | Chen 2022 §3.3 |
| Chen 2022 C22-2 | Bilateral putamen seed FC | striatal/reward | — | null (P>.05) | within-group pre/post (null) | seed-based WB | same as C22-1 | P>.05 | Chen 2022 §3.3 |
| Chen 2022 C22-3 | L globus pallidus seed FC | sensorimotor + DMN discussion | postcentral[SM], IPL[DMN/FPN] | ↓ FC decreased (post vs pre) | within-group pre/post | seed-based WB | same as C22-1 | t=−4.00/k142; t=−4.86/k26 | Chen 2022 Table2+Fig1A |
| Chen 2022 C22-4 | R globus pallidus seed FC | sensorimotor + DMN | SMG/postcentral/superior parietal[SM]; IPL/precuneus[DMN] | ↓ 6+ clusters FC decreased | within-group pre/post | seed-based WB | same as C22-1 | NR | Chen 2022 Table2+Fig1B |
| Chen 2022 C22-5 | R posterior caudate seed FC | — | cerebellar[cerebellar], calcarine/lingual[visual] | ↓ FC decreased | within-group pre/post | seed-based WB | same as C22-1 | NR; cluster coordinates/t/k NV | Chen 2022 §3.3+Fig1C |
| Chen 2022 C22-6 | ΔFC vs HAMD-17 | [SM-DMN] | — | negative correlation (FC↓ = HAMD↓) | correlation | cluster-level correlation | uncorrected | r=−0.6/−0.54/−0.474/−0.513 | Chen 2022 §3.4 |
| He 2023 H23-1 | ALFF (baseline cross-sectional) | reward (VSi, authors) | dlPFC/OFC[FPN/reward], ACC/dmPFC[DMN/SN], hippocampus/amygdala[limbic] | baseline MDD vs HC: cortical ↓, subcortical ↑ | between-group (cross-sectional) | WB voxelwise | two-way repeated ANCOVA + GRF voxel p<.001 + cluster p<.05 | NR | He 2023 Fig S1 |
| He 2023 H23-2 | ALFF (group×time interaction) | reward (VSi, authors' core target) | [reward/subcortical] | interaction: VSi baseline MDD>HC, post-↓ (normalized) | group×time | seed-based WB | same (HC test–retest mask excludes time effect) | F=21.0849 | He 2023 Table2+Fig S3 |
| He 2023 H23-3 | ReHo (group×time interaction) | reward | [reward/subcortical] | interaction: post-↓ (same direction as ALFF normalization) | group×time | seed-based WB | same as H23-1 | F=18.8081 | He 2023 Table2 |
| He 2023 H23-4 | FCD (group×time interaction) | reward | [reward/subcortical] | interaction: post-↓ (normalized) | group×time | seed-based WB | same as H23-1 | F=22.3406 | He 2023 Table2 |
| He 2023 H23-5 | ALFF group×time (precuneus cluster) | — | precuneus/PCC[DMN] | interaction: post-↑ (opposite direction to VSi) | group×time | seed-based WB | same as H23-1 | F=23.4968 | He 2023 Table2 |
| He 2023 H23-6 | ReHo group×time (precuneus cluster) | — | [DMN] | interaction: post-↑ | group×time | seed-based WB | same as H23-1 | F=19.1346 | He 2023 Table2 |
| He 2023 H23-7 | ALFF (8-wk within taVNS) | — | ACC/dmPFC[DMN/SN], thalamus[subcortical] | ↓ (post vs pre) | within-group pre/post | WB voxelwise | same as H23-1 | NR | He 2023 Fig S2 |
| He 2023 H23-8 | HC 8-wk test–retest | — | — | null (HC two scans no significant change after mask; supports treatment effect) | within-group pre/post (HC control null) | WB voxelwise | same + HC test–retest mask (unc p<.05) | — | He 2023 Methods/Results |
| He 2023 H23-9 | VSi–precuneus-cluster FC (post vs pre) | reward-DMN regulation (authors) | [reward↔DMN] | ↓ FC decreased post-treatment (t=2.095, p=.048) | within-group pre/post (ROI-cluster FC) | seed(ROI)-cluster FC | not reported (exploratory) | t=2.095, p=.048 | He 2023 Results+Fig S4 |
| He 2023 H23-10 | Control VSi–mOFC connection (Brainnetome mask) | [reward internal] | — | null (t=−0.057, p=.934) | within-group pre/post (control FC null) | ROI-ROI FC | — | t=−0.057, p=.934 | He 2023 Results |
| He 2023 H23-11 | Imaging change vs HAM-D remission | reward | [reward-DMN] | negative correlation (VSi ALFF/FC drop↑ = remission↑); however main text phrases as "positive correlations" — wording inconsistent with r sign; reported as written with flag | correlation | cluster-level correlation | uncorrected | r=−0.763; r=−0.869 (both p<.001) | He 2023 Results |
| Zhang 2022 Z22-1 | L vCa seed FC | cortico-striatal | vPFC[FPN/reward] | interaction (baseline low → post-↑ normalized): baseline t=−3.42; MDD post t=3.17; HC t=−4.38 | group×time + post-hoc | seed-based WB | mixed-ANCOVA + GRF voxel p<.001 + cluster p<.05 | NR | Zhang 2022 §3.3.1 |
| Zhang 2022 Z22-2 | L vCa seed FC | cortico-striatal | SOG[visual] | interaction (baseline high → post-↓ normalized); ΔFC vs ΔHAMD r=−0.586,p=.045 | group×time + correlation | seed-based WB | same as Z22-1 | NR | Zhang 2022 §3.3.1 |
| Zhang 2022 Z22-3 | R GP seed FC | cortico-striatal | SOG[visual] | interaction (abnormally high → post-↓ normalized) | group×time + post-hoc | seed-based WB | same as Z22-1 | NR | Zhang 2022 §3.3.2 |
| Zhang 2022 Z22-4 | L NAc seed FC | cortico-striatal/reward | MTG[DMN/auditory] | interaction (post-↓) | group×time + post-hoc | seed-based WB | same as Z22-1 | NR | Zhang 2022 §3.3.3 |
| Zhang 2022 Z22-5 | R NAc seed FC | cortico-striatal/reward | dmPFC[DMN] | interaction (baseline low → post-↑ normalized); baseline FC vs HAMD r=−0.584,p=.046 (negative) | group×time + correlation | seed-based WB | same as Z22-1 | r=−0.584; other statistics NR | Zhang 2022 §3.3.4 |
| Zhang 2022 Z22-6 | R NAc seed FC | cortico-striatal | vlPFC[FPN/reward] | interaction (baseline low → post-↑ normalized) | group×time + post-hoc | seed-based WB | same as Z22-1 | NR | Zhang 2022 §3.3.4 |
| Zhang 2022 Z22-7 | L dCa seed FC | cortico-striatal | cuneus[visual/DMN] | interaction (abnormally high → post-↓ normalized); ΔFC vs ΔHAMD r=0.629,p=.029 | group×time + correlation | seed-based WB | same as Z22-1 | r=0.629; other statistics NR | Zhang 2022 §3.3.1 |
| Zhang 2022 Z22-8 | R dCa seed FC (intra-striatal) | cortico-striatal (striatal internal) | [reward/motor] | interaction (intra-striatal connectivity post-↑) | group×time + post-hoc | seed-based WB | same as Z22-1 | NR | Zhang 2022 §3.3.x |
| Liao 2023 L23-1 | ICA within-network FC (MDD vs HC baseline) | DMN (ICA network, authors) | — | ↓ baseline MDD<HC | between-group (cross-sectional) | ICA network-level (FNC) | one-sample FDR p<.05 >50-voxel mask; MDD vs HC two-sample FWE p<.05 | FWE p<.05 | Liao 2023 §3.1+Fig3 |
| Liao 2023 L23-2 | ICA within-network (group×time interaction) | insula network (~SN) | — | interaction significant (FWE p<.05; coordinates NV) | group×time | ICA network-level (FNC) | FWE p<.05 | FWE p<.05 | Liao 2023 §3.2 |
| Liao 2023 L23-3 | ICA within-network (time/main group effects) | time: insula, DAN, LFPN, PVN (visual), cerebellar; group: ASN, VAN, AN (auditory) | — | time/main group effects significant | time/main group | ICA network-level (FNC) | FWE p<.05 | NR | Liao 2023 §3.2 |
| Liao 2023 L23-4 | ICA within-network (post-hoc treatment pre/post) | — | — | null (post-hoc FDR p<.05: all pairwise treatment comparisons ns across the significant networks) | within-group pre/post post-hoc (null) | ICA network-level (FNC) | post-hoc FDR p<.05 | — | Liao 2023 §3.2 |
| Liao 2023 L23-5 | Between-network FNC (FDR-corrected) | ICA between-network | — | null (FDR-corrected between-network FNC: no significant difference) | between-group | ICA network-level (FNC) | FDR correction | — | Liao 2023 §3.3 |
| Liao 2023 L23-6 | Between-network FNC (uncorrected p<.05; authors downgraded) | DMN/DAN/FPN/visual | — | baseline between-group (uncorrected, low evidence strength) | between-group (exploratory uncorrected) | ICA network-level (FNC) | uncorrected p<.05 (figure caption explicit) | p<.05 uncorr | Liao 2023 §3.3+Fig4 |
| Liao 2023 L23-7 | Between-network FNC TECAS (uncorrected) | DMN/DAN/FPN/visual | — | post-TECAS: DMN–DAN↑, DMN–RFPN↑, PVN–RFPN↓ | within-group pre/post (exploratory) | ICA network-level (FNC) | uncorrected p<.05 | p<.05 uncorr | Liao 2023 §3.3+Fig5 |
| Liao 2023 L23-8 | Between-network FNC escitalopram (uncorrected) | DMN/DAN/FPN/VAN/visual | — | post-escitalopram: mostly opposite directions to TECAS | within-group pre/post (exploratory) | ICA network-level (FNC) | uncorrected p<.05 | p<.05 uncorr | Liao 2023 §3.3+Fig6 |
| Liao 2023 L23-9 | Baseline within-network vs baseline clinical | DMN (SFG medial/ACC ∈ DMN) | — | positive correlation (connectivity↑ = symptom↑) | correlation (cross-sectional) | ICA network-level (FNC) | not reported | NR | Liao 2023 §3.4 |
| Liao 2023 L23-10 | Δnetwork vs Δclinical | — | — | null (both treatment groups: within-network change uncorrelated with clinical change) | correlation (null) | ICA network-level (FNC) | — | — | Liao 2023 §3.4 |
| Ma 2023 M23-1 | ALFF (baseline cross-sectional) | authors: no label | mSFG/BA9[DMN] | ↓ baseline MDD<HC (peak t=−5.0714) — Results/Table3: MDD lower than HC; Discussion "increased" is original-text typo; per Results set as ↓ | between-group (cross-sectional) | WB voxelwise | 2×2 repeated ANCOVA + GRF voxel P<.005 + cluster P<.05; covariates sex/age/education/FD | t=−5.0714 | Ma 2023 Table3+Fig4 |
| Ma 2023 M23-2 | ReHo (baseline cross-sectional) | — | mSFG/BA9[DMN] | ↓ baseline MDD<HC (peak t=−4.762) | between-group (cross-sectional) | WB voxelwise | same as M23-1 | t=−4.762 | Ma 2023 Table3+Fig4 |
| Ma 2023 M23-3 | R mSFG seed FC (group×time) | — | IOG[visual], MTG[DMN/auditory] | interaction: abnormally-high FC post-↓/normalized (TECAS and escitalopram same trend); time main P<.0001; group main ns | group×time | seed-based WB | same GRF | NR | Ma 2023 Table4/5+Fig5 |
| Ma 2023 M23-4 | mSFG seed FC (time main effect) | — | SFG/BA32[DMN/ACC] | time main effect significant | time main effect | seed-based WB | same as M23-1 | peak t=5.2784 | Ma 2023 Table6+Fig6 |
| Ma 2023 M23-5 | ALFF/ReHo group×time | — | — | null (ALFF/ReHo reported only as baseline cross-sectional differences; no group×time interaction cluster) | group×time (null) | seed-based WB | same as M23-1 | — | Ma 2023 Results |
| Ma 2023 M23-6 | FC change vs clinical | — | — | null (no FC–clinical correlation reported) | correlation (not done/null) | cluster-level correlation | — | — | Ma 2023 (no correlation section) |
| Ma 2024 M24-1 | ALFF (baseline cross-sectional) | authors: DMN (insula-DMN framework) | insula[SN] (differs from authors' label) | ↓ baseline MDD<HC (t=−4.298) | between-group (cross-sectional) | WB voxelwise | GRF voxel P<.005 + cluster P<.05 | t=−4.298 | Ma 2024 Table2+Fig2 |
| Ma 2024 M24-2 | L insula seed FC (baseline cross-sectional) | DMN (insula-DMN) | MFG[FPN], ACC[SN/DMN] | ↓ baseline MDD<HC (two clusters) | between-group (cross-sectional) | seed-based WB | same as M24-1 | t=−5.29; t=−6.08 | Ma 2024 Table3+Fig3 |
| Ma 2024 M24-3 | L insula seed FC (post ↑) | DMN | ACC/OFC[SN/reward] | ↑ post-FC increased (normalized direction) | within-group pre/post | seed-based WB | same as M24-1 | peak t=3.42 | Ma 2024 Table4+Fig4 |
| Ma 2024 M24-4 | L insula seed FC (post ↑) | DMN | SFG[DMN/FPN], SMA[sensorimotor] | ↑ post-FC increased; coordinates/voxel count flagged (original-text internal inconsistency) | within-group pre/post | seed-based WB | same as M24-1 | peak t=4.34 (abstract 3.34) | Ma 2024 Table4+Fig4 |
| Ma 2024 M24-5 | Normalization test (post-MDD vs HC) | DMN | same clusters as M24-3 and M24-4 | null (post-treatment difference vs HC no longer significant, but p does not reach "full normalization"; both clusters ns) | normalization test (between-group; post-MDD vs HC null) | seed-based WB | two-sample t | p=.0746; p=.1872 | Ma 2024 §3.4 |
| Ma 2024 M24-6 | Insula-FC change vs HAMD-17 change | DMN | [SN/reward] | negative correlation (FC↑ = HAMD↓) | correlation | cluster-level correlation | uncorrected | r=−0.3375, P=.025 | Ma 2024 §3.5+Fig5 |
| Yi 2022 Y22-1 | ReHo (first-session post vs pre; no ↑ cluster) | — | MCC/SMA/central pre-post[SN+SM], calcarine/lingual[visual] | ↓ 7 clusters ReHo decreased immediately post-stimulation; no ↑ cluster | within-group pre/post (paired) | WB voxelwise | paired t + cluster FWE p<.05 | T=−3.886 to −5.845 | Yi 2022 Table3 |
| Yi 2022 Y22-2 | ReHo change vs 4-wk HAMD improvement | — | MCC/SMA[SN/sensorimotor] | positive correlation (immediate ReHo change pattern corresponds to 4-wk improvement) | correlation | cluster-level correlation | figure caption uncorrected | r=0.62; r=0.74 | Yi 2022 Results+Fig |
| Guo 2024 G24-1 | Global topology Eglob (global efficiency) | global integration (authors' topology framework) | — | ↑ (0.269→0.274, t=−4.537, p<.001, FDR) | within-group pre/post (paired) | graph theory — global | paired t + FDR p<.05 (covariates age/sex/FD) | t=−4.537, p<.001 | Guo 2024 §3.2+Table3 |
| Guo 2024 G24-2 | Global topology Lp (characteristic path length) | global integration | — | ↓ (0.790→0.760, t=6.353, p<.001, FDR) | within-group pre/post | graph theory — global | same as G24-1 | t=6.353, p<.001 | Guo 2024 §3.2+Table3 |
| Guo 2024 G24-3 | Other global properties (gamma/lambda/sigma/Eloc) | — | — | null ("No significant difference was observed in other global properties") | within-group pre/post (null) | graph theory — global | FDR p<.05 | — | Guo 2024 §3.2 |
| Guo 2024 G24-4 | Node attributes (L angular gyrus) | authors: DMN/FPN boundary (angular gyrus) | [DMN] | ↑ L angular gyrus DC, NE post-↑ (other nodes ns) | within-group pre/post | graph theory — node | FDR p<.05 | t=−7.003; t=−2.123 | Guo 2024 §3.3+Table |
| Guo 2024 G24-5 | NBS component (edge-level FC) | authors: DMN–FPN, DMN–CON, FPN–CON between-network connections | — | ↓ (component connections post-↓ overall); 16 nodes/10 edges (NBS FWE p<.05) | within-group pre/post (NBS) | NBS (network edges) | NBS 5,000 permutation FWE p<.05 (body text also marks FDR corrected) | 16 nodes 10 edges; p<.05 | Guo 2024 §3.4+Fig5 |
| Guo 2024 G24-6 | Topology change vs HAMD change | DMN/global topology | — | correlation (Lp drop correlates with HAMD drop; angular-DC rise correlates with HAMD drop — both indicating larger topology change with larger improvement) | correlation | cluster-level correlation | p<.05 | r=0.616; r=−0.703 | Guo 2024 §3.5 |
| Garcia 2021 G21-1 | sgACC task activation | Not assigned (task activation) | sgACC[DMN] | ↑ e-RAVANS > i-RAVANS (Exh > Inh) | within-subject cross-over (e- vs i-RAVANS) | WB task activation + ROI SVC | SVC cluster FWE p<.05 (voxel p<.001 uncorrected) | 100 vox; MNI (−2,24,−8); T=2.9; p=.032 | Fig 2A; Table 2 |
| Garcia 2021 G21-2 | OFC task activation | Not assigned (task activation) | OFC[limbic] | ↑ e-RAVANS > i-RAVANS (Exh > Inh) | within-subject cross-over (e- vs i-RAVANS) | WB task activation + ROI SVC | SVC cluster FWE p<.05 (voxel p<.001 uncorrected) | 559 vox; MNI (−14,30,−26); T=4.1; p=.022 | Fig 2A; Table 2 |
| Garcia 2021 G21-3 | vmPFC task activation | Not assigned (task activation) | vmPFC[DMN] | ↑ e-RAVANS > i-RAVANS (Exh > Inh) | within-subject cross-over (e- vs i-RAVANS) | WB task activation + ROI SVC | SVC cluster FWE p<.05 (voxel p<.001 uncorrected) | 722 vox; MNI (−6,52,−14); T=4.1; p=.027 | Fig 2A; Table 2 |
| Garcia 2021 G21-4 | Hypothalamus → R dlPFC FC (stress-task run) | CAN | hypothalamus[CAN]; dlPFC[FPN] | ↑ e-RAVANS > i-RAVANS (Exh > Inh) | within-subject cross-over (e- vs i-RAVANS) | Seed-based FC + WB cluster correction; seeds are a priori functional ROIs from prior RAVANS fMRI studies (not atlas-defined) | cluster-corrected* | 878 vox; MNI (30,34,24); T=4.3; p<.001 | Table 2 |
| Garcia 2021 G21-5 | NTS → LC FC (L; stress-task run) | CAN | NTS[CAN/brainstem]; LC[CAN/brainstem] | ↑ e-RAVANS > i-RAVANS (Exh > Inh) | within-subject cross-over (e- vs i-RAVANS) | same as G21-4 | cluster-corrected* | 393 vox; MNI (−10,−32,−26); T=3.2; p=.007 | Table 2 |
| Garcia 2021 G21-6 | NTS → vmPFC FC (L; stress-task run) | CAN | NTS[CAN/brainstem]; vmPFC[DMN] | ↑ e-RAVANS > i-RAVANS (Exh > Inh) | within-subject cross-over (e- vs i-RAVANS) | same as G21-4 | cluster-corrected* | 227 vox; MNI (−10,54,0); T=3.9; cluster-corrected | Table 2 |
| Garcia 2021 G21-7 | DRN → PCC FC (administration run) | CAN | DRN[brainstem]; PCC[DMN] | ↑ i-RAVANS > e-RAVANS (Inh > Exh) | within-subject cross-over (i- vs e-RAVANS) | same as G21-4 | cluster-corrected* | 418 vox; MNI (−12,−54,44); T=4.93; p<.001 | Table 2 |
| Garcia 2021 G21-8 | DRN → dlPFC FC (L; administration run) | CAN | DRN[brainstem]; dlPFC[FPN] | ↑ e-RAVANS > i-RAVANS (Exh > Inh) | within-subject cross-over (e- vs i-RAVANS) | same as G21-4 | cluster-corrected* | 198 vox; MNI (−40,44,28); T=4.67; p=.035 | Table 2 |
| Garcia 2021 G21-9 | sgACC activation × ΔBDI (e-RAVANS) | Not assigned (task activation) | sgACC[DMN] | negative β (greater activation → greater BDI reduction) | within-session e-RAVANS brain–behaviour correlation | GLM correlation; t(16); adjusted for baseline BDI & antidepressant use | uncorrected (exploratory) | β=−5.01; p=.007; Adj R²=.38 | Table 3 |
| Garcia 2021 G21-10 | OFC activation × ΔBDI (e-RAVANS) | Not assigned (task activation) | OFC[limbic] | negative β (greater activation → greater BDI reduction) | within-session e-RAVANS brain–behaviour correlation | same as G21-9 | uncorrected (exploratory) | β=−6.64; p=.004; R²=.43 | Table 3 |
| Garcia 2021 G21-11 | vmPFC activation × ΔBDI (e-RAVANS) | Not assigned (task activation) | vmPFC[DMN] | negative β (greater activation → greater BDI reduction) | within-session e-RAVANS brain–behaviour correlation | same as G21-9 | uncorrected (exploratory) | β=−4.64; p=.007; R²=.39 | Table 3 |
| Garcia 2021 G21-12 | NTS → LC FC × ΔBDI (e-RAVANS) | CAN | NTS[CAN/brainstem]; LC[CAN/brainstem] | negative β (greater FC change → greater BDI reduction) | within-session e-RAVANS brain–behaviour correlation | same as G21-9 | uncorrected (exploratory) | β=−9.02; p=.001; R²=.55 | Table 3 |
| Garcia 2021 G21-13 | NTS → vmPFC FC × ΔBDI (e-RAVANS) | CAN | NTS[CAN/brainstem]; vmPFC[DMN] | negative β (greater FC change → greater BDI reduction) | within-session e-RAVANS brain–behaviour correlation | same as G21-9 | uncorrected (exploratory) | β=−8.48; p=.004; R²=.43 | Table 3 |
| Garcia 2021 G21-14 | sgACC activation × ΔSTAI (e-RAVANS) | Not assigned (task activation) | sgACC[DMN] | negative β (greater activation → greater STAI reduction) | within-session e-RAVANS brain–behaviour correlation | same as G21-9 | uncorrected (exploratory) | β=−5.50; p=.004; R²=.72 | Table 3 |
| Garcia 2021 G21-16 | OFC activation × ΔSTAI (e-RAVANS) | Not assigned (task activation) | OFC[limbic] | negative β (greater activation → greater STAI reduction) | within-session e-RAVANS brain–behaviour correlation | same as G21-9 | uncorrected (exploratory) | β=−6.74; p=.005; R²=.71 | Table 3 |
| Garcia 2021 G21-17 | vmPFC activation × ΔSTAI (e-RAVANS) | Not assigned (task activation) | vmPFC[DMN] | negative β (greater activation → greater STAI reduction) | within-session e-RAVANS brain–behaviour correlation | same as G21-9 | uncorrected (exploratory) | β=−4.83; p=.004; R²=.71 | Table 3 |
| Garcia 2021 G21-18 | NTS → LC FC × ΔSTAI (e-RAVANS) | CAN | NTS[CAN/brainstem]; LC[CAN/brainstem] | negative β (greater FC change → greater STAI reduction) | within-session e-RAVANS brain–behaviour correlation | same as G21-9 | uncorrected (exploratory) | β=−10.02; p=.003; R²=.73 | Table 3 |
| Garcia 2021 G21-19 | NTS → vmPFC FC × ΔSTAI (e-RAVANS) | CAN | NTS[CAN/brainstem]; vmPFC[DMN] | negative β (greater FC change → greater STAI reduction) | within-session e-RAVANS brain–behaviour correlation | same as G21-9 | uncorrected (exploratory) | β=−8.19; p=.012; R²=.67 | Table 3 |
| Garcia 2021 G21-20 | i-RAVANS session: all brain–behaviour associations null (activation & FC vs ΔBDI and ΔSTAI, across all ROIs) | Mixed (task activation + CAN) | all parent ROIs [DMN/limbic/CAN] | null (all p>.05; β not tabulated) | within-session i-RAVANS brain–behaviour correlation | same as G21-9 | uncorrected (exploratory) | — | Table 3; p.194 |

1. Row tags identify each report by first author and publication year (e.g., F16 = Fang et al., 2016; G21 = Garcia et al., 2021), followed by a sequential number for each effect coded within that report.

2. Networks are coded twice for every effect. Author-reported labels are reproduced verbatim from the primary report. Where a report names an affected region but gives no network label, the review team assigned the region to a network by established anatomical correspondence (e.g., anterior insula–salience network; medial prefrontal cortex/precuneus–default mode network; dorsolateral prefrontal cortex–frontoparietal network); borderline or multi-network regions are shown as boundary or dual assignments (e.g., [DMN/FPN]). These attributions are descriptive labels for synthesis only and are not mechanistic claims; regional signal change is not interpreted as network-level psychological function.

3. Direction is signed relative to the comparator stated in each row; opposing directions are never merged in the vote counts, and conflicts within or across reports are flagged (see note 5). In the Multiplicity correction column, the method is transcribed as used in the primary report; “not reported” or “—” indicates that no correction was specified. In the Statistic column, values are transcribed as reported; “NR” indicates that the primary report asserts the effect but tabulates no extractable test statistic in its text or result tables (values shown only in figures, or reported narratively), and “—” indicates a null analysis for which no statistic was reported.

4. Twenty-seven rows report explicitly null findings (Direction = null); five further rows contain embedded null sub-comparisons within significant findings (F17-8, T18-3, T18-4, M22-5, S24-3). Null and positive findings are coded in parallel. Rows are effect-level: reports from the same parent trial (C1–C9; 321 enrolled and 293 analyzed MDD, 144 healthy controls) contribute separate rows without double-counting participants.

5. Cells marked with an asterisk, NV, or flagged indicate original-text inconsistencies, unverified coordinates, or author-flagged gaps (e.g., S23-3 sign mismatch; M24-4 peak t-value mismatch (4.34 vs abstract 3.34); H23-11 sign/wording mismatch; W18-6 typical-band insular cluster coordinates NV; L23-2 and C22-5 coordinates NV; G21-4–G21-8 FDR in Methods vs FWE in the Table 2 header). Flagged rows are retained for transparency; they are treated with the correction level reported as primary and are summarized in the flagged-items list in the main text.

**Abbreviations (as used in this table). Stimulation and clinical terms:** taVNS, transcutaneous auricular vagus nerve stimulation; TECAS, transcutaneous electrical cranial–auricular acupoint stimulation; RAVANS, respiratory-gated auricular vagal afferent nerve stimulation; e-RAVANS/i-RAVANS, exhalatory-gated/inhalatory-gated RAVANS; MDD, major depressive disorder; TRD, treatment-resistant depression; HC, healthy controls; BDI, Beck Depression Inventory; HAMD (HAM-D), Hamilton Depression Rating Scale; HAMA (HAM-A), Hamilton Anxiety Rating Scale; STAI, State–Trait Anxiety Inventory.

**MRI and analysis:** fMRI, functional magnetic resonance imaging; BOLD, blood oxygenation level-dependent; ICA, independent component analysis; FC, functional connectivity; FNC, functional network connectivity; ALFF, amplitude of low-frequency fluctuations; ReHo, regional homogeneity; FCD, functional connectivity density; WB, whole-brain; ROI, region of interest; GRF, Gaussian random field; FWE, family-wise error; FDR, false discovery rate; SVC, small-volume correction; NBS, network-based statistics; GLM, general linear model; ANCOVA, analysis of covariance; ML, machine learning; LOOCV, leave-one-out cross-validation; AUC, area under the curve; MAE, mean absolute error; MNI, Montreal Neurological Institute; BA, Brodmann area; FD, framewise displacement; NR, not reported; NV, not verified (see notes 3 and 5); “—”, no statistic reported for a null analysis.

**Networks:** CAN, central autonomic network; DMN, default mode network; SN, salience network; FPN, frontoparietal network; CCN, cognitive control network; CEN, central executive network; FPCN, frontoparietal control network; CON, cingulo-opercular network; DAN/VAN, dorsal/ventral attention network; LFPN/RFPN, left/right frontoparietal network; ASN, anterior salience network; PVN, primary visual network (Liao et al., 2023 nomenclature); AN, auditory network; SM, sensorimotor network; CSPT, cortico-striato-pallido-thalamic.

**Regions and seeds:** NTS, nucleus tractus solitarius; DRN, dorsal raphe nucleus; LC, locus coeruleus; MH/LH, medial/lateral hypothalamus; NAc, nucleus accumbens; VSi, inferior ventral striatum; vCa/dCa, ventral/dorsal caudate; GP, globus pallidus; OFC, orbitofrontal cortex; mOFC, medial orbitofrontal cortex; OPFC, orbitoprefrontal cortex; PFC, prefrontal cortex; mPFC (MPFC), medial PFC; vmPFC, ventromedial PFC; dmPFC, dorsomedial PFC; vPFC (vlPFC), ventrolateral PFC; DLPFC (dlPFC), dorsolateral PFC; ACC, anterior cingulate cortex; sgACC, subgenual ACC; rACC, rostral ACC; MCC, mid-cingulate cortex; PCC, posterior cingulate cortex; SMA, supplementary motor area; SFG, superior frontal gyrus; mSFG, medial superior frontal gyrus; MFG, middle frontal gyrus (labelled medial frontal gyrus in Tu et al., 2018); IPL, inferior parietal lobule; SMG, supramarginal gyrus; IOG/SOG, inferior/superior occipital gyrus; MTG/ITG/STG, middle/inferior/superior temporal gyrus; TPJ, temporoparietal junction.
