# Supplementary material for: Large-scale brain network modulation by auricular vagus neuromodulation in major depressive disorder: a systematic review of neuroimaging evidence
Source: Front Psychiatry. 2026 Sep 30;17:1972200. doi: 10.3389/fpsyt.2026.1972200 (PMC13664290; doi:10.3389/fpsyt.2026.1972200)
Supplement: Supplementary file 5 [file Table4.docx]

**Supplementary Table S4. Methodological quality (risk of bias) of the 17 included neuroimaging reports, appraised with the revised JBI Critical Appraisal Tool for quasi-experimental studies (Barker et al., 2024).**

**Part A. Item legend — official wording (Barker et al., JBI Evidence Synthesis 2024;22(3):378–388) and 2020 to 2024 crosswalk**

| **Bias domain** | **Item (2024)** | **Official item wording (Barker et al., 2024)** | **Equivalent item in the 2020 tool (Tufanaru et al.)** |
| --- | --- | --- | --- |
| Internal validity — temporal precedence | Q1 | Is it clear in the study what is the 'cause' and what is the 'effect' (no confusion about which variable comes first)? | Q1 |
| Internal validity — selection and allocation | Q2 | Was there a control group? (Note: an independent, separate control group, not the pre-test group in a single-group pre–post design.) | Q4 |
| Internal validity — confounding | Q3 | Were participants included in any comparisons similar? (Official note: in a single-group pre–post design the same people are compared, so answer Yes.) | Q2 |
| Internal validity — administration of intervention | Q4 | Were the participants included in any comparisons receiving similar treatment/care, other than the exposure or intervention of interest? (N/A when no between-group comparison of co-interventions exists, e.g. single-group pre–post.) | Q3 |
| Internal validity — assessment, detection and measurement | Q5 | Were there multiple measurements of the outcome, both pre and post the intervention/exposure? | Q5 |
| Internal validity — assessment, detection and measurement | Q6 | Were the outcomes of participants included in any comparisons measured in the same way? | Q7 |
| Internal validity — assessment, detection and measurement | Q7 | Were outcomes measured in a reliable way? (Applies to every design, including single-arm; standardized acquisition and processing pipelines qualify.) | Q8 |
| Internal validity — participant retention | Q8 | Was follow-up complete and, if not, were differences between groups in terms of their follow-up adequately described and analyzed? | Q6 |
| Statistical conclusion validity | Q9 | Was appropriate statistical analysis used? (Includes multiplicity control, power, effect sizes, and test-assumption checks.) | Q9 |

Note. The substantive content of the nine items is unchanged between the 2020 version (Tufanaru et al., JBI Manual for Evidence Synthesis, chapter 3) and the revised 2024 version (Barker et al., JBI Evidence Synthesis 2024;22:378–388); the 2024 revision reorders the items and assigns bias-domain labels. The 2020 numbering is retained in the last column for reference, as the earlier version remains in wide use; all judgments in Parts B and C follow the 2024 order and official definitions.

**Part B. Item-level judgments for the 17 included neuroimaging reports**

| **Report (cohort)** | **Q1 temporal** | **Q2 control** | **Q3 similar** | **Q4 co-interv.** | **Q5 pre/post** | **Q6 same way** | **Q7 reliable** | **Q8 follow-up** | **Q9 statistics** | **Score (Y/applicable)** | **Grade** |
| --- | --- | --- | --- | --- | --- | --- | --- | --- | --- | --- | --- |
| Fang 2016 (C1) | Y | Y | U | Y | Y | Y | Y | U | Y | 7/9 (77.8%) | Moderate |
| Liu 2016 (C1) | Y | Y | U | Y | Y | Y | Y | U | Y | 7/9 (77.8%) | Moderate |
| Fang 2017 (C1) | Y | Y | U | Y | Y | Y | Y | U | Y | 7/9 (77.8%) | Moderate |
| Tu 2018 (C1) | Y | Y | U | Y | Y | Y | Y | U | Y | 7/9 (77.8%) | Moderate |
| Wang 2018 (C1) | Y | Y | U | Y | Y | Y | Y | U | Y | 7/9 (77.8%) | Moderate |
| Ma 2022 (C2) | Y | Y | Y | Y | Y | Y | Y | Y | Y | 9/9 (100%) | High |
| Sun 2023 (C2) | Y | N | Y | N/A | Y | Y | Y | U | Y | 6/8 (75.0%) | Moderate |
| Sun 2024 (C2) | Y | N | Y | N/A | Y | Y | Y | Y | Y | 7/8 (87.5%) | Moderate |
| Chen 2022 (C3) | Y | N | Y | N/A | Y | Y | Y | Y | Y | 7/8 (87.5%) | Moderate |
| He 2023 (C4) | Y | Y | Y | Y | Y | Y | Y | U | Y | 8/9 (88.9%) | Moderate |
| Zhang 2022 (C5) | Y | Y | Y | Y | Y | Y | Y | Y | Y | 9/9 (100%) | High |
| Liao 2023 (C6) | Y | Y | U | Y | Y | Y | Y | U | N | 6/9 (66.7%) | Low |
| Ma 2023 (C6) | Y | Y | U | Y | Y | Y | Y | Y | Y | 8/9 (88.9%) | Moderate |
| Ma 2024 (C6) | Y | N | Y | N/A | Y | Y | Y | Y | Y | 7/8 (87.5%) | Moderate |
| Yi 2022 (C7) | Y | N | Y | N/A | Y | Y | Y | Y | Y | 7/8 (87.5%) | Moderate |
| Guo 2024 (C8) | Y | N | Y | N/A | Y | Y | Y | Y | Y | 7/8 (87.5%) | Moderate |
| Garcia 2021 (C9) | Y | N | Y | Y | Y | Y | Y | U | Y | 7/9 (77.8%) | Moderate |

**Note.** Y = Yes; N = No; U = Unclear (not reported in the published full text); N/A = not applicable (logically inapplicable, e.g. item Q4 in single-group designs). Judgments were made at the study level for each report’s primary neuroimaging outcome(s).
**Summary:** 2 reports High (Ma 2022, 9/9; Zhang 2022, 9/9), 14 reports Moderate, 1 report Low (Liao 2023, 6/9). At the cohort level: C5 High; C1/C2/C3/C4/C7/C8/C9 Moderate; C6 Moderate-to-Low.

**Grading rule (pre-specified; Methods §2.5).** The JBI prescribes no numerical cut-off scores for its critical appraisal tools; grades were therefore defined a priori. The proportion of "yes" judgments among applicable items (excluding N/A) was calculated (≥70% / 50–69% / <50%) and interpreted alongside five items judged critical for causal inference in this evidence base — control group (Q2), participant comparability (Q3), reliable outcome measurement (Q7), complete follow-up/retention (Q8), and appropriate statistical analysis including multiplicity control (Q9): reports with ≥70% "yes" and no "no"/"unclear" on any critical item were graded High; reports with 50–69% "yes", or ≥70% "yes" with any "no"/"unclear" judgment, were graded Moderate, with single-arm/within-subject studies lacking an independent control group (Q2 = No by design) capped at Moderate; reports with <50% "yes", a "no" on Q9, or "no" judgments on two or more critical items were graded Low.
Abbreviations of neuroimaging metrics and brain regions are defined in Supplementary Table S3.

**Part C. Per-item justifications organized by bias domain**

| **Report (cohort)** | **Bias domain** | **Item** | **Judgment** | **Justification (one factual sentence per item)** |
| --- | --- | --- | --- | --- |
| Fang 2016 / Liu 2016 / Fang 2017 / Tu 2018 / Wang 2018 (C1, 5 reports) | Temporal precedence | Q1 | Y | taVNS intervention (4-wk course, single session, or 6-min stimulation) clearly precedes the post-treatment or post-stimulation imaging endpoint. |
| same C1 set | Selection/allocation | Q2 | Y | Independent sham-control arm (non-vagal ear-margin site), with real and sham allocated in parallel (non-randomized sequential enrollment). |
| same C1 set | Confounding | Q3 | U | Non-randomized sequential enrollment; no between-group baseline demographic or clinical comparability table is reported in the published reports. |
| same C1 set | Intervention administration | Q4 | Y | Identical visit/scan schedule across groups; the only systematic difference is stimulation site (auricular concha vs ear margin). |
| same C1 set | Assessment/detection/measurement | Q5 | Y | 0-wk and 4-wk (or within-session on/off blocks) paired measurements; 4-wk clinical follow-up available for correlation analyses. |
| same C1 set | Assessment/detection/measurement | Q6 | Y | Same scanner (1.5 T for Fang/Liu, 3 T for the later reports) and an identical processing pipeline used for both arms. |
| same C1 set | Assessment/detection/measurement | Q7 | Y | Standardized rs-fMRI processing; primary contrasts at Z>2.6 with cluster-level correction (GRF/FWE/FDR); head-motion exclusions reported explicitly (e.g., 49→35→34 for Fang 2016). |
| same C1 set | Participant retention | Q8 | U | Of 49 recruited, 34/34/38/37/37 were analyzed across the five reports; group-wise attrition counts and reasons are not described. |
| same C1 set | Statistical conclusion validity | Q9 | Y | Group × time interaction models with cluster-level correction; correlation analyses performed at cluster level or adjusted for age/sex; null results reported (Liu 2016 left-amygdala seed; Tu 2018 four control seeds; Wang 2018 slow-4/typical/sham bands). |
| Ma 2022 (C2) | Temporal precedence | Q1 | Y | Single 30-min taVNS session followed by immediate rs-fMRI; temporal order clear. |
| Ma 2022 | Selection/allocation | Q2 | Y | 40 healthy controls received identical pre/post scans (HC ReHo pre vs post t=1.155, p=.25), constituting a valid pre/post control arm. |
| Ma 2022 | Confounding | Q3 | Y | TRD and HC groups comparable; analyses covaried sex, age, education, and FD. |
| Ma 2022 | Intervention administration | Q4 | Y | Both groups scanned in the same session with the same protocol (controls also received taVNS as a within-design control exposure); no differential co-interventions. |
| Ma 2022 | Assessment/detection/measurement | Q5 | Y | Pre/post rs-fMRI for both groups. |
| Ma 2022 | Assessment/detection/measurement | Q6 | Y | Same scanner, same pipeline. |
| Ma 2022 | Assessment/detection/measurement | Q7 | Y | GRF voxel P<.01 + cluster P<.05 (two-sided); Bonferroni-corrected post-hoc tests. |
| Ma 2022 | Participant retention | Q8 | Y | 40 MDD + 40 HC all entered analysis; zero attrition. |
| Ma 2022 | Statistical conclusion validity | Q9 | Y | 2×2 repeated-measures ANCOVA with interaction + Bonferroni post-hoc (P<.0125); null findings (ReHo/FC group main effects; clinical correlation null) reported in parallel. |
| Sun 2023 (C2) | Temporal precedence | Q1 | Y | Single 30-min taVNS with scan 3 min post-stimulation; temporal order clear. |
| Sun 2023 | Selection/allocation | Q2 | N | 54 healthy controls measured at baseline only (cross-sectional); no post-stimulation control measurement, so they cannot serve as a control for the pre/post treatment effect. |
| Sun 2023 | Confounding | Q3 | Y | MDD pre/post comparison within the same people (per JBI manual, single-group = Yes); covariates per team convention. |
| Sun 2023 | Intervention administration | Q4 | N/A | No post-intervention control group; no between-group co-intervention comparison. |
| Sun 2023 | Assessment/detection/measurement | Q5 | Y | Pre/post rs-fMRI. |
| Sun 2023 | Assessment/detection/measurement | Q6 | Y | Same scanner, same pipeline. |
| Sun 2023 | Assessment/detection/measurement | Q7 | Y | GRF voxel P<.005 + cluster P<.05; clusters <60 voxels discarded. |
| Sun 2023 | Participant retention | Q8 | U | 99 screened → 4 withdrew (claustrophobia/mental issues) and 5 excluded for motion (4 TRD, 1 HC) → 44 TRD + 46 HC analyzed; retention reported, but no differential-attrition analysis. |
| Sun 2023 | Statistical conclusion validity | Q9 | Y | GRF cluster-corrected pre/post contrasts; the baseline precuneus–PCC FC vs HAMD-17 association (r=−0.288, P=.032) is an uncorrected exploratory analysis and is labeled as such. |
| Sun 2024 (C2) | Temporal precedence | Q1 | Y | 8-wk taVNS precedes endpoint assessment; baseline FC predicts outcome — temporal order clear. |
| Sun 2024 | Selection/allocation | Q2 | N | Single-arm 86 MDD; no healthy-control arm and no sham. |
| Sun 2024 | Confounding | Q3 | Y | Same cohort pre/post and in the prediction modeling (single group). |
| Sun 2024 | Intervention administration | Q4 | N/A | Single-arm; no between-group co-intervention comparison. |
| Sun 2024 | Assessment/detection/measurement | Q5 | Y | 0/8-wk rs-fMRI (57 participants with paired scans). |
| Sun 2024 | Assessment/detection/measurement | Q6 | Y | Same scanner, same pipeline. |
| Sun 2024 | Assessment/detection/measurement | Q7 | Y | Standardized FC feature extraction. |
| Sun 2024 | Participant retention | Q8 | Y | 86 enrolled → 9 motion exclusions → 77 modeled; 57 with longitudinal data (42 responders / 15 non-responders); numbers and destinations explicit. |
| Sun 2024 | Statistical conclusion validity | Q9 | Y | Two-stage feature selection (P<.01) + nested leave-one-out cross-validation + 5,000-permutation testing; the ML framework is appropriate; R²=0.44, AUC=0.856 (internally validated, exploratory). |
| Chen 2022 (C3) | Temporal precedence | Q1 | Y | 8-wk taVNS precedes the post-treatment imaging. |
| Chen 2022 | Selection/allocation | Q2 | N | Single-arm; no healthy-control arm and no sham. |
| Chen 2022 | Confounding | Q3 | Y | Same 22 participants pre/post (per JBI manual, single-group = Yes). |
| Chen 2022 | Intervention administration | Q4 | N/A | Single-arm. |
| Chen 2022 | Assessment/detection/measurement | Q5 | Y | 0/8-wk rs-fMRI. |
| Chen 2022 | Assessment/detection/measurement | Q6 | Y | Same scanner, same pipeline. |
| Chen 2022 | Assessment/detection/measurement | Q7 | Y | GRF voxel p<.01 + cluster p<.05; clusters <15 voxels discarded. |
| Chen 2022 | Participant retention | Q8 | Y | 30 enrolled → "5 excluded by inclusion criteria, 3 dropped out" → 22 analyzed; numbers and reasons categorized. |
| Chen 2022 | Statistical conclusion validity | Q9 | Y | Paired pre/post analysis with GRF cluster correction; bilateral NAc (P=.07) and putamen seed null results reported in parallel. |
| He 2023 (C4) | Temporal precedence | Q1 | Y | 8-wk taVNS precedes the endpoint scan. |
| He 2023 | Selection/allocation | Q2 | Y | 23 healthy controls independently recruited and scanned twice over the same 8-wk interval without taVNS (test–retest control), constituting a valid pre/post control arm. |
| He 2023 | Confounding | Q3 | Y | Age/sex/education/FD covaried in the ANCOVA. |
| He 2023 | Intervention administration | Q4 | Y | Groups differ only by taVNS exposure; scan and follow-up schedules identical. |
| He 2023 | Assessment/detection/measurement | Q5 | Y | 0/8-wk rs-fMRI for both groups. |
| He 2023 | Assessment/detection/measurement | Q6 | Y | Same scanner, same pipeline. |
| He 2023 | Assessment/detection/measurement | Q7 | Y | GRF voxel p<.001 + cluster p<.05 two-tailed; a healthy-control test–retest mask (unc p<.05) excludes time effects. |
| He 2023 | Participant retention | Q8 | U | 28 enrolled → 22 completed both scans; reasons for the six losses and their distribution are not reported. |
| He 2023 | Statistical conclusion validity | Q9 | Y | Two-way repeated-measures ANCOVA + GRF; the VSi–mOFC control-connection null result (p=.934) reported in parallel. |
| Zhang 2022 (C5) | Temporal precedence | Q1 | Y | 8-wk taVNS precedes the post-treatment scan; prospectively registered (NCT03607331, 2018-07-31). |
| Zhang 2022 | Selection/allocation | Q2 | Y | 16 healthy controls scanned twice over 8 wk as a test–retest control arm. |
| Zhang 2022 | Confounding | Q3 | Y | Sex/age/FD covaried; explicit inclusion/exclusion criteria. |
| Zhang 2022 | Intervention administration | Q4 | Y | Same scan schedule; only taVNS exposure differs. |
| Zhang 2022 | Assessment/detection/measurement | Q5 | Y | 0/8-wk rs-fMRI. |
| Zhang 2022 | Assessment/detection/measurement | Q6 | Y | Same scanner, same pipeline. |
| Zhang 2022 | Assessment/detection/measurement | Q7 | Y | ANCOVA interaction: voxel p<.001 + cluster p<.05 (two-sided). |
| Zhang 2022 | Participant retention | Q8 | Y | 16 enrolled → 1 excluded for comorbid severe anxiety → 15 analyzed; reason explicit. |
| Zhang 2022 | Statistical conclusion validity | Q9 | Y | Repeated-measures ANCOVA interaction + paired/independent t post-hoc with covariates; bidirectional (normalization in both directions) and null striatal-seed results all reported. |
| Liao 2023 (C6) | Temporal precedence | Q1 | Y | 8-wk TECAS/escitalopram precedes the endpoint scan. |
| Liao 2023 | Selection/allocation | Q2 | Y | Independent escitalopram comparator arm (21 cases) plus 49 healthy controls as an independent group. |
| Liao 2023 | Confounding | Q3 | U | Non-randomized assignment to TECAS/escitalopram arms; no three-group baseline equivalence table is reported in the published text. |
| Liao 2023 | Intervention administration | Q4 | Y | Same 8-wk assessment schedule; escitalopram is a within-design control intervention (not a co-intervention); other care consistent. |
| Liao 2023 | Assessment/detection/measurement | Q5 | Y | 0/8-wk rs-fMRI (3 T Skyra). |
| Liao 2023 | Assessment/detection/measurement | Q6 | Y | Same scanner, same group-level ICA pipeline. |
| Liao 2023 | Assessment/detection/measurement | Q7 | Y | Within-network MDD vs HC with FWE p<.05; one-sample FDR p<.05 used to construct masks; measurement pipeline standardized. |
| Liao 2023 | Participant retention | Q8 | U | Of 50 MDD, 5 dropped out (2 TECAS / 3 escitalopram); per-group imaging-analysis n and the handling of dropouts in the pre/post comparison are not reported. |
| Liao 2023 | Statistical conclusion validity | Q9 | N | Headline between-network FNC treatment contrasts (DMN–DAN, DMN–RFPN, PVN–RFPN) are reported at uncorrected p<.05 (figure caption explicit) and are entirely null after FDR correction; the primary inferential contrasts lack multiplicity protection for whole-brain/network-level analysis, and post-hoc within-network FDR pairwise contrasts are likewise null. |
| Ma 2023 (C6) | Temporal precedence | Q1 | Y | 8-wk TECAS/escitalopram precedes the endpoint scan. |
| Ma 2023 | Selection/allocation | Q2 | Y | Active-comparator arm plus 51 healthy controls as an independent group. |
| Ma 2023 | Confounding | Q3 | U | Non-randomized assignment to TECAS/escitalopram; baseline equivalence across arms is not reported in the published text. |
| Ma 2023 | Intervention administration | Q4 | Y | Same 8-wk schedule; no differential co-interventions. |
| Ma 2023 | Assessment/detection/measurement | Q5 | Y | 0/8-wk rs-fMRI. |
| Ma 2023 | Assessment/detection/measurement | Q6 | Y | Same scanner, same pipeline. |
| Ma 2023 | Assessment/detection/measurement | Q7 | Y | GRF voxel P<.005 + cluster P<.05; covariates sex/age/education/FD. |
| Ma 2023 | Participant retention | Q8 | Y | 51 MDD / 51 HC analysis flow reported; no unexplained attrition. |
| Ma 2023 | Statistical conclusion validity | Q9 | Y | 2×2 repeated-measures ANCOVA + GRF with a clear interaction/main-effect/post-hoc hierarchy; ALFF/ReHo group×time nulls and absent FC–clinical correlations reported in parallel. |
| Ma 2024 (C6) | Temporal precedence | Q1 | Y | 8-wk TECAS precedes the endpoint scan. |
| Ma 2024 | Selection/allocation | Q2 | N | 34 healthy controls measured at baseline only (cross-sectional); no post-intervention control measurement, so they cannot serve as a control for the 8-wk pre/post effect. |
| Ma 2024 | Confounding | Q3 | Y | Same 34 MDD pre/post (single-group = Yes). |
| Ma 2024 | Intervention administration | Q4 | N/A | No post-intervention control group. |
| Ma 2024 | Assessment/detection/measurement | Q5 | Y | 0/8-wk rs-fMRI. |
| Ma 2024 | Assessment/detection/measurement | Q6 | Y | Same scanner, same pipeline. |
| Ma 2024 | Assessment/detection/measurement | Q7 | Y | GRF voxel P<.005 + cluster P<.05. |
| Ma 2024 | Participant retention | Q8 | Y | All 34 TECAS-arm cases analyzed (same arm as Ma 2023; flow reported in the parent trial). |
| Ma 2024 | Statistical conclusion validity | Q9 | Y | Paired pre/post plus post-treatment vs HC normalization tests (two-sample t, p=.0746/.1872, reported honestly as non-significant); insula–ACC FC change vs HAMD-17 correlation (r=−0.338, P=.025) is cluster-level exploratory. |
| Yi 2022 (C7) | Temporal precedence | Q1 | Y | First 30-min taVNS precedes the immediate post-stimulation scan; 4-wk clinical follow-up. |
| Yi 2022 | Selection/allocation | Q2 | N | Single-arm; no healthy-control arm and no sham. |
| Yi 2022 | Confounding | Q3 | Y | Same participants pre/post (single-group = Yes). |
| Yi 2022 | Intervention administration | Q4 | N/A | Single-arm. |
| Yi 2022 | Assessment/detection/measurement | Q5 | Y | Pre/post rs-fMRI. |
| Yi 2022 | Assessment/detection/measurement | Q6 | Y | Same scanner, same pipeline (DPARSF). |
| Yi 2022 | Assessment/detection/measurement | Q7 | Y | Paired t + cluster-level FWE p<.05. |
| Yi 2022 | Participant retention | Q8 | Y | 22 enrolled → 2 motion exclusions → imaging N=20; 4-wk clinical correlation n=18; numbers and reasons explicit. |
| Yi 2022 | Statistical conclusion validity | Q9 | Y | Paired t with cluster FWE; ReHo–HAMD correlations (r=0.62/0.74) are figure-caption uncorrected and exploratory; main conclusions do not rest on them. |
| Guo 2024 (C8) | Temporal precedence | Q1 | Y | 4-wk taVNS precedes the endpoint scan. |
| Guo 2024 | Selection/allocation | Q2 | N | Single-arm 19 MDD; no healthy-control arm and no sham. |
| Guo 2024 | Confounding | Q3 | Y | Same 19 participants pre/post (single-group = Yes). |
| Guo 2024 | Intervention administration | Q4 | N/A | Single-arm. |
| Guo 2024 | Assessment/detection/measurement | Q5 | Y | 0/4-wk rs-fMRI. |
| Guo 2024 | Assessment/detection/measurement | Q6 | Y | Same scanner, same pipeline (GRETNA). |
| Guo 2024 | Assessment/detection/measurement | Q7 | Y | 160-node FC + graph theory with a standardized pipeline; covariates age/sex/mean FD. |
| Guo 2024 | Participant retention | Q8 | Y | All 19 analyzed; no attrition reported. |
| Guo 2024 | Statistical conclusion validity | Q9 | Y | Global/node paired t with FDR p<.05; NBS component edges with 5,000-permutation FWE p<.05; null global properties reported; topology–HAMD correlations secondary. |
| Garcia 2021 (C9) | Temporal precedence | Q1 | Y | In a within-subject cross-over with two fMRI sessions one week apart, respiratory-gated stimulation (e-RAVANS vs i-RAVANS) was delivered within each session and stress-task fMRI plus BDI/STAI were measured before and after it; temporal order is unambiguous. |
| Garcia 2021 | Selection/allocation | Q2 | N | No independent, separate control group: both conditions were active auricular stimulation compared within the same participants, who served as their own comparators; the authors state the study "was not designed as a clinical trial." |
| Garcia 2021 | Confounding | Q3 | Y | Comparisons are within the same 18 participants completing both sessions, so the comparison groups are identical (within-subject comparison = Yes per the JBI manual logic); condition order was computer-randomized and order effects were tested and non-significant. |
| Garcia 2021 | Intervention administration | Q4 | Y | Both sessions took place within one week in the follicular phase with identical IAPS task and stimulation duration and comparable current intensity (4.89 vs 5.01 mA, p=.75); stable SSRI/SNRI medication (3/20, ≥8 weeks) was included as a model covariate. |
| Garcia 2021 | Assessment/detection/measurement | Q5 | Y | Pre- and post-stimulation stress-task fMRI runs and BDI/STAI ratings acquired in every session. |
| Garcia 2021 | Assessment/detection/measurement | Q6 | Y | Identical scanner, acquisition, and AFNI/CONN preprocessing (RETROICOR physiological-noise correction) across sessions. |
| Garcia 2021 | Assessment/detection/measurement | Q7 | Y | Standardized 3T pipeline; primary activation contrasts used small-volume correction with cluster-level FWE p<.05 (voxel height p<.001); seed-based FC used predefined ROIs from the group’s prior RAVANS work. |
| Garcia 2021 | Participant retention | Q8 | U | 20 participants attended the e-RAVANS session and 18 the i-RAVANS session; analyses were restricted to the 18 complete paired datasets, but the full text does not report why two participants did not complete the second session. |
| Garcia 2021 | Statistical conclusion validity | Q9 | Y | Factorial session × time models with SVC cluster-level FWE for the primary activation/FC contrasts; brain–behavior GLMs (adjusted for baseline BDI and medication) are uncorrected and appropriately exploratory; the multiplicity-correction label discrepancy (FDR in Methods vs FWE in the Table 2 header) is flagged as an inconsistency in the effect-level coding table (Supplementary Table S3) and the affected rows are excluded from the vote counts. |
